# Sierra platinum: a fast and robust peak-caller for replicated ChIP-seq experiments with visual quality-control and -steering

**DOI:** 10.1186/s12859-016-1248-6

**Published:** 2016-09-15

**Authors:** Lydia Müller, Daniel Gerighausen, Mariam Farman, Dirk Zeckzer

**Affiliations:** 1 Bioinformatics Group, Department of Computer Science, University of Leipzig, Härtelstraße 16–18, Leipzig, 04107 Germany; 2 Image and Signal Processing Group, Department of Computer Science, University of Leipzig, Augustusplatz 10, Leipzig, 04109 Germany

**Keywords:** ChIP-seq, Peak-caller, Histone modifications, Replicate analysis

## Abstract

**Background:**

Histone modifications play an important role in gene regulation. Their genomic locations are of great interest. Usually, the location is measured by ChIP-seq and analyzed with a peak-caller. Replicated ChIP-seq experiments become more and more available. However, their analysis is based on single-experiment peak-calling or on tools like PePr which allows peak-calling of replicates but whose underlying model might not be suitable for the conditions under which the experiments are performed.

**Results:**

We propose a new peak-caller called ‘Sierra Platinum’ that allows peak-calling of replicated ChIP-seq experiments. Moreover, it provides a variety of quality measures together with integrated visualizations supporting the assessment of the replicates and the resulting peaks, as well as steering the peak-calling process.

**Conclusion:**

We show that Sierra Platinum outperforms currently available methods using a newly generated benchmark data set and using real data from the NIH Roadmap Epigenomics Project. It is robust against noisy replicates.

**Electronic supplementary material:**

The online version of this article (doi:10.1186/s12859-016-1248-6) contains supplementary material, which is available to authorized users.

## Background

The genomic localization of DNA bound proteins, such as transcription factors or (modified) histone proteins, is frequently used to identify regulatory relationships between regulators and the regulated genes in different cell types. The state-of-the-art method for this purpose is ChIP-seq (Chromatin immunoprecipitation sequencing). First, DNA is fragmented. Fragments bound to the protein of interest are extracted and subsequently sequenced. The resulting sequencing reads can then be mapped onto the genome. As neither the experimental procedure nor the mapping of the reads to the genome are perfect, the existence of a read at a specific genomic location does not always imply that the measured protein was bound. Additional measurements with unspecific or without antibodies serve as background measurements and allow to cope with the noisy signal. Peak-calling compares the signals of the ChIP-seq experiment with background measurements to find genomic regions where the experiment is significantly enriched over the background. Thus, the genomic location of the protein measured is determined.

Several peak-callers are available for single experiments. The largest difference between the different methods is the statistical framework used to model the background. Peaks are annotated at positions where the observed number of reads is significantly higher than the one expected by chance given the background model. Koohy et al. [1] and Wilbanks et al. [2] give a good overview and comparison of state-of-the-art peak-callers used in several published studies.

**Fig. 1:**
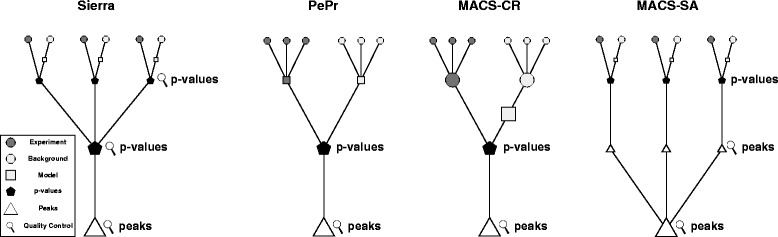
Overview of the multiple-replicate peak-calling process showing the basic steps of multiple-replicate peak-calling for Sierra Platinum, PePr, MACS-CR (combine replicates approach using MACS as single-experiment peak-caller), and MACS-SA (combine peaks approach using MACS as single-experiment peak-caller). All peak-caller extract the parameters of the underlying model (*squares*) from the background data (*dark circles*) and use it to calculate *p*-values (*pentagons*) indicating how significantly enriched the experiment (*light circles*) is. Based on the *p*-values, peaks (*triangles*) are calculated. Quality control (*magnifier*) is provided usually alongside with the peaks. *Only Sierra* allows to examine the *quality during* the peak-calling process, while all other methods only allow to examine the quality of the peaks obtained

Peak-calling for replicated ChIP-seq experiments, however, is not well supported. In the following, we refer to the replicated ChIP-seq experiments as ‘replicates’ and to their combined analysis as ‘multiple-replicate peak-calling’. Only two approaches exist that use a single experiment peak-caller and either combine the replicates before peak-calling or combine the peaks obtained from the single experiments (see Fig. 1: MACS-CR and MACS-SA, respectively). Combining the replicates before peak-calling requires equal library sizes (total number of mapped reads) across all replicates or down-sampling to a common library size. For example, the NIH Roadmap Epigenomics Project uses the down-sampling approach to avoid artificial differences in the signal strength with uniform depth of at most 30 million reads before merging the replicates [3]. Similarly, MACS scales the two libraries which are compared to the same amount of reads to make experiment and background library comparable [4]. Down-sampling, however, may lead to an overestimation of the noise level. It is not possible to incorporate weights for the replicates based on their quality or to backtrack the source of a specific signal to the supporting replicates.

Combining the peaks of the single experiments includes all peaks in any of the replicates. Thus, replicates with poor peak-call quality can have a large effect on the final result. Furthermore, very long peaks can occur in the final peak set by merging neighboring narrow peaks from different replicates. While none of the replicates predicts those broad peaks, the final result contains them.

Further, only PePr [5] explicitly supports replicates of the same condition during peak-calling (see Fig. 1: PePr). It uses a binomial model that expects the same dispersion for experiment and background. However, for, e.g., experiments performed at different sequencing centers, it is unknown if this condition holds and consequently it is not guaranteed that the results obtained using this model are reliable. Also, PePr down-samples all libraries to the same size and thus might overestimate the noise level.

Besides the so far mentioned peak-caller, there are several peak-callers for differential peak-calling, i.e., finding peaks which occur in only one of two groups of samples. The underlying statistical model assumes that there are basically three types of peaks: peaks occurring in both groups of samples and peaks occurring in only one of the two groups. In particular, none of the groups is treated as background for which no peak should be found. Similar to PePr, those peak-callers apply methods from differential gene expression which fit two negative binomial distributions to the two groups for each locus and compare the data based on these distributions. For example, csaw [6] uses the edgeR package [7] to find differential peaks, while diffBind [8] uses peaks predicted on each sample and compares the peaks based on read counts within the peaks using the edgeR package [7]. We do not aim at finding differential peaks but peaks with respect to a background measurements which is a very different task from a statistical point of view [6]. We will therefore not further evaluate those peak-callers in detail.

The currently available approaches for replicate peak-calling are neither designed to assess the replicates’ quality nor to handle replicates of different quality. Moreover, the replicates’ quality can not be incorporated during peak-calling. In the case of combining the peak-calls of the single experiments, it is in principle possible to introduce weights to account for different qualities of the replicates. However, doing this in a statistically sound way is hardly possible since non-significant positions are not provided by the peak-caller.

To close this gap, we propose Sierra Platinum, a new, fast and robust peak-caller for replicated ChIP-seq experiments with visual quality-control and -steering. Sierra Platinum uses a model based on the Poisson distribution together with the inverse normal method to combine the replicates. Multiple quality measures are computed and visualized. This allows to judge the replicate’s quality as well as the quality of the resulting peaks. A subset of replicates can be selected and weighted to obtain the combined peaks. Figure 1 shows the principal differences and commonalities between the current methods for multiple-replicates and Sierra Platinum (see also Chapter “Method” in the Additional file 1).

We optimized the computation of the combined peaks to keep time and memory requirements minimal (see also Chapter “Optimization” in the Additional file 1).

Currently, no benchmark data set for assessing the quality of peak-calling methods is available. Peak-calling methods are evaluated against real data without known ‘ground truth’. To be able to assess the quality of peak-calling methods based on ‘ground truth’, we created a benchmarking data set (see also Chapter “Benchmark Data Set and Quality Measures” in the Additional file 1).

Based on this benchmarking data set we assessed and compared the quality of Sierra Platinum, PePr, and existing approaches using single-experiment peak-callers. It shows that our approach allows an optimized combination of the replicates yielding less false positive peaks and stable true positive ones, while avoiding also false negative peaks, that is, avoiding missing peaks (see also Chapter “Evaluation” in the Additional file 1).

Finally, we applied Sierra Platinum to H1 chromatin methylation and acetylation data. We found that our predictions match the results known from literature better than previously published ones (see also Chapter “Results” in the Additional file 1).

## Methods

The multiple-replicate peak-calling process of Sierra Platinum is depicted in Fig. 1. Sierra Platinum computes the multiple-replicate peak-calls in three phases: (I) construct *p*-values per replicate, (II) combine these *p*-values, (III) compute the final peaks and additional information based on the combined *p*-values. The individual steps of each phase are described next (see also Chapter “Method” in the Additional file 1).

**Phase I** The steps of this phase are computed for each data set—experiment and background of each replicate—or each replicate separately. First, the windows are constructed. Each window has a start position and a size. All tags overlapping this window are counted. Empty windows are discarded.

Afterwards, the Poisson distribution of the tag counts of the windows is computed. Similar to the approach by Zhang et al. [4], this serves as a model for computing the single experiment *p*-values. As in general the amount of used and mapped material differs between experiment and background, the experiments are scaled. This allows the comparison between experiment and background.

Next, the 1, 5, and 10 k neighborhoods of each window are determined. Now, single experiment *p*-values are calculated for each window and each replicate based on the global *λ* of the normalized Poisson distribution and the *λ* values computed for each neighborhood.

1 $$ \lambda = max\{\lambda_{global}, \lambda_{1k}, \lambda_{5k}, \lambda_{10k}\}  $$

Strong but very local changes in the tag distribution along the genome might lead to significant changes in both background and experiment compared to the *λ* computed. Therefore, we check whether the background is significant given the chosen *λ*. If this is the case, then we use the tag count of the corresponding window as *λ* estimate instead and recalculate the corresponding *p*-value. These *p*-values determine the peaks of each replicate. To reduce the effect from the correlation between the *p*-values computed, they are transformed into so-called *q*-values. As the *q*-values are corrected *p*-values, they are subsequently called *p*-values.

**Phase II** During this phase, the information computed for the replicates is combined. Sierra calculates for each window and replicate one *p*-value. To obtain a single *p*-value for each window, the inverse normal method [9] is used to combine *p*-values of the different replicates of the same window. First, the correlation between the replicates is determined. This information is used for adapting the combination of the replicates performed next. On the one hand, the replicates need to be correlated to compute justified, combined peaks. On the other hand, correlation is problematic for applying the combination method proposed. Therefore, the combination has to be corrected for the correlation found.

The replicates are filtered and weighted based on their quality assessment. To compute the combined *p*-values for each window, the inverse normal method is applied. During this step, replicates, which are filtered out, are discarded, and the correlation coefficients and weights established previously are applied.

**Phase III** During this phase, the combined *p*-values are used for computing additional quality information as well as narrow and broad peaks. The combined *p*-values are again correlated and therefore converted into *q*-values. As the *q*-values are corrected *p*-values, they are subsequently called *p*-values again. Finally, the narrow peaks and the broad peaks of the final combined result are determined.

**Quality parameters** Sierra assesses several quality parameters for the single experiments, the combined *p*-values, and the final peaks (see Fig. 1). This allows the user to assess and control the quality of the peak-calls. For each replicate, Sierra provides the following quality measurements. We provide the theoretical and empirical read distributions allowing the user to judge whether the Poisson distribution is a good fit for the data. Since the background model has to be comparable to the experiment’s read count distribution, experiment and background are scaled to the same library size (same total number of reads). Sierra allows to inspect the normalized distribution to judge the comparability of both models. To evaluate the quality of the reads used for peak-calling, the base quality distribution of the mapped reads is depicted. The *p*-value distribution indicates if the significance level allows peak-calls of high quality. The median *p*-value distribution calculated across all replicates allows to assess whether a replicate is an outlier or fits into the replicate ensemble. Similarly, the distribution of significant windows for all chromosomes of the replicate and the respective medians across all replicates allow to draw conclusions about the replicates fit to the ensemble of replicates. Furthermore, they allow to identify chromosomes with an odd number of significant windows.

To support the calculation of the combined *p*-value, the correlation between the replicates is calculated based on the so-called probits. Probits are a transformation of the *p*-values of the single experiments and are a by-product of the inverse normal method. The correlations indicate how strong the replicates agree on a common signal. We provide the distribution of the combined *p*-values which allows to adjust the cut-off and *p*-value correction method to the data. After peak-calling, we provide the overlap of the final significant windows with the significant window in each replicate. In contrast to the correlation, this measurement already incorporates the chosen *p*-value cut-off (see also Chapter “Method” in the Additional file 1).

## Results

### Benchmark

We evaluate the results of Sierra Platinum and compare it to PePr [5], and MACS-SA and MACS-CR using MACS2 [4] based on the benchmarking data set (gold standard). Except for the ‘noise free’ set of replicates, we generated peak-calls using all replicates with equal weight (Sierra1, without down-weighting or excluding bad replicates), excluding the ‘noisy’ replicates (Sierra 2), and down-weighting the ‘noisy’ replicates (Sierra 3). In all data sets, the ‘noisy’ replicates could be identified using at least one quality measurement. Additional information is provided in the Chapter “Evaluation” of the Additional file 1.

**Fig. 2:**
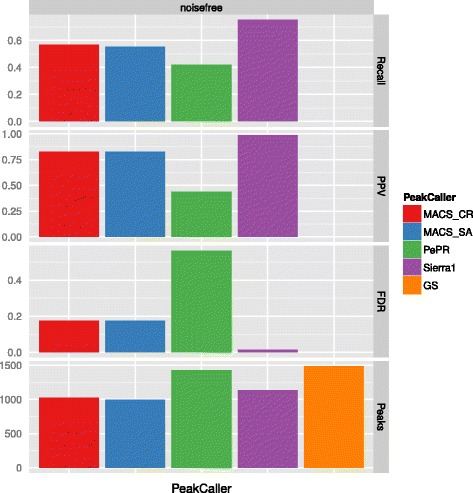
Evaluation results for the noise-free data set (6 replicates)

**Fig. 3:**
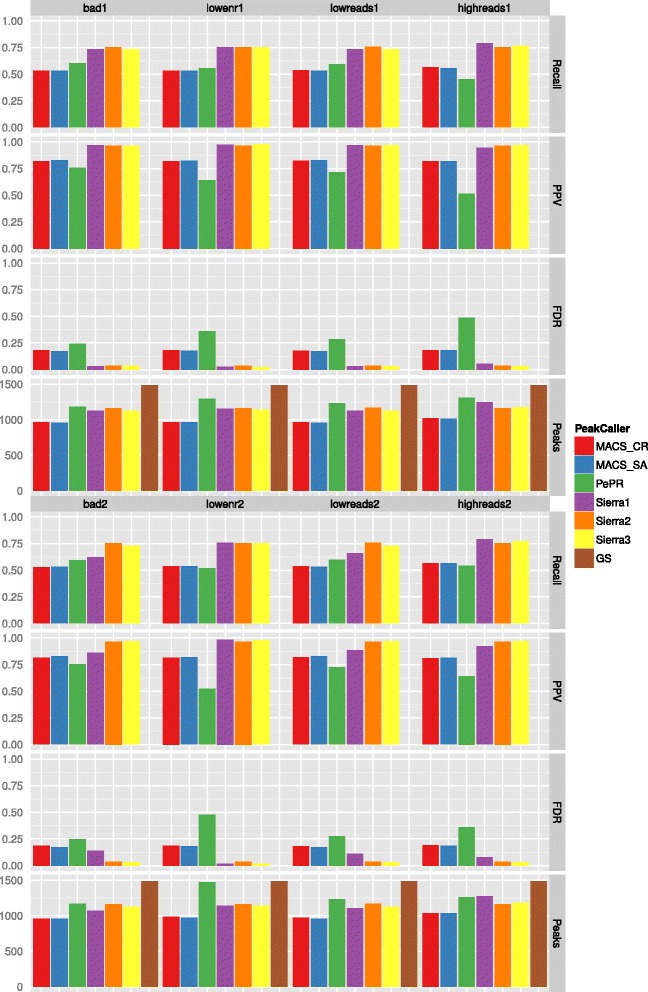
Evaluation results for data sets with noise. *First column*: low sequencing quality. *Second column*: low enrichment. *Third column*: too low sequencing depth. *Fourth column*: too high sequencing depth. *First four rows*: one bad replicate; three replicates in total. *Second four rows*: two bad replicates; four replicates in total

**Fig. 4:**
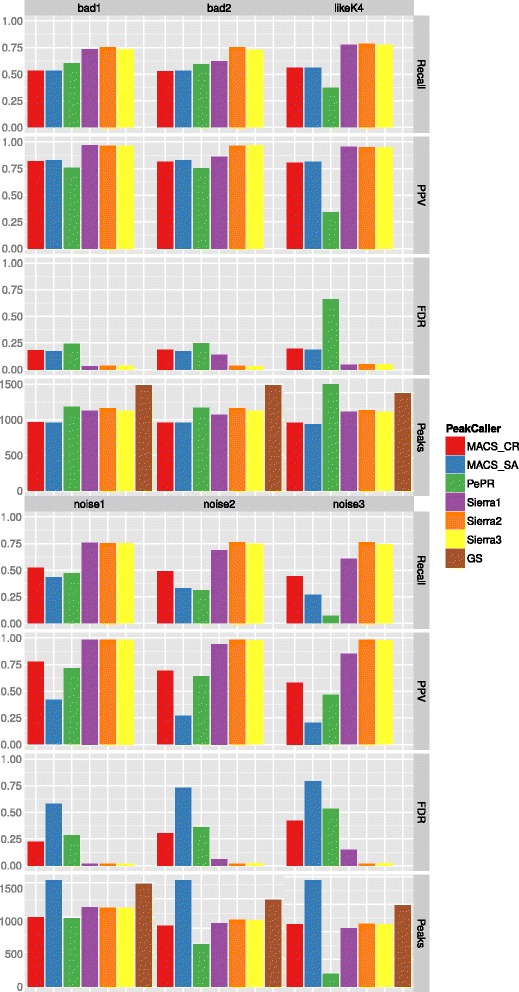
Evaluation results for quality deficits in some of the data sets. *First four rows*, left to right: one under-sequenced replicate with low enrichment and low quality, two under-sequenced replicates with low enrichment and low quality, and a mixture of quality inspired by real data for H3K4me3 in embryonic stem cells. *Second four rows*, left to right: one, two, and three noisy replicates

The results are shown in Figs. 2, 3 and 4. From top to bottom we report Recall, Positive Predictive Value (PPV), False Discovery Rate (FDR), and Number of Peaks (see also Chapter “Benchmark Data Set and Quality Measures” in the Additional file 1). In addition to the results obtained by the different peak-calling methods, we also provide the number of peaks that would be optimally found (gold standard, GS).

First of all, we checked the performance on noise-free data. However, the noise-free data is far away from a data set produced in a laboratory. Thus, we introduced different types of noise, which could also be observed in real data. To be able to assess the effects of the factors independently, we generated data sets containing only one type of noise. In the following, we will show the effects of the following factors: pure sequencing quality, too low enrichment, too low sequencing depth (i.e., under-sequencing), and too high sequencing depth (i.e., over-sequencing). Furthermore, we test for the combination of bad quality, low enrichment, and low sequencing depth (bad1, bad2, likeK4), and noisy signals (see also Chapter “Benchmark Data Set and Quality Measures” in the Additional file 1).

**Noise-free data** For the noise-free data (Fig. 2), Sierra Platinum has the highest recall, followed by both variations of MACS having more than 10 % less recall, while PePr has a recall of ≈30 % less. Compared to MACS (both approaches) and PePr also the PPV and FDR are much better for Sierra Platinum. The high number of peaks generated by PePr comes at the expense of a high FDR. In total, best results are obtained by Sierra Platinum.

**Pure sequencing quality** For pure sequencing quality, we used two benchmarking data sets. Each of them contains two good replicates and either one or two bad replicates (Fig. 3, first column). It does not make a large difference whether we add one or two data sets with a low sequencing quality, and thus more sequencing errors, to two high quality data sets: the results are quite similar to those of the noise-free data set. Excluding the low quality replicates improves the results a bit with respect to recall, PPV, and FDR. Almost as good is the improvement when down-weighting instead of excluding the replicates of low sequencing quality. Compared to MACS and PePr, any approach of Sierra Platinum (all replicates, removing bad replicates, down-weighting bad replicates) performs better with respect to all three quality measurements.

**Low enrichment** A low enrichment, i.e., the signal to noise ratio is low, does not much affect the performance of all peak-callers (Fig. 3, second column). The result of Sierra Platinum can be equally well improved by deleting or down-weighting the low enriched replicates.

**Low sequencing depth** A low sequencing depth does not have a strong influence on the peak-calling quality of Sierra Platinum (Fig. 3, third column). Deleting or down-weighting the replicates with low sequencing quality improves the results even more. Deleting is just marginally better than down-weighting. Still, MACS-CR and MACS-SA have lower recall but a higher PPV than PePr. The recall is about 3 % lower than in the noise-free data for MACS.

**High sequencing depth** Replicates with a too high sequencing depth are not effecting the peak-calls of MACS-CR and MACS-SA (Fig. 3, fourth column). This might be an effect of the two good quality replicates always included in the data sets. Surprisingly, two replicates with a high sequencing depth produce better results than just one replicate with too many reads in the case of PePr (recall and PPV increase by about 10 %). The results of Sierra Platinum in its default settings are affected by the replicates with the too high sequencing depth. Deleting the replicates with too many reads, the recall drops slightly but the PPV increases. Down-weighting these replicate shows similar results. In the case of two over-sequenced samples, the results of the effect of down-weighting or deleting bad replicates can be seen even stronger.

**Bad replicates** We evaluated Sierra Platinum, MACS-SA, MACS-CR, and PePr also on data sets with a mixture of noises. The data sets bad1 and bad2 (Fig. 4) are composed of two good replicates, and 1 respectively 2 under-sequenced replicates with low enrichment and low quality. The recall drops and the FDR tends to increase with the amount of bad replicates in Sierra Platinum, but this can be efficiently compensated by deleting or down-weighting the replicates. In comparison to the other peak-callers, even the native version of Sierra Platinum has higher recall and lower FDR.

The data set *likeK4* contains a mixture of qualities (Fig. 4), i.e., experiment and background may not have comparable data quality and the quality between replicates differs as well. Similarly to the previous data set, recall and FDR are better compared to the other peak-callers independently of the approach used for Sierra Platinum.

**Noisy data sets** We compared all peak-callers on data sets containing 1, 2, or 3 noisy replicates (Fig. 4, bottom row), i.e., replicates with a different signal track. Each data set is filled up with replicates of perfect quality until they contain 6 replicates in total.

The recall of all peak-callers decreases with an increasing amount of noise. In particular MACS-SA and PePr show a strong drop in the recall. Furthermore, the FDR increases strongly. The strongest increase of the FDR is found for MACS-SA since the peaks of all replicates are simply merged. Thus, all peaks from the noisy replicates are kept.

**Summary** Even using the defaults settings–no deletion or down-weighting of replicates—the performance of Sierra Platinum on *noisy* data is superior compared to the performance of other peak-callers on *noise-free* data. Deleting or down-weighting replicates increases the performance of Sierra Platinum on noisy data reaching the performance of Sierra Platinum on noise-free data. Thus, the method implemented in Sierra Platinum is robust against any kind of noise in the data. Moreover, the implemented user interactions for deleting and down-weighting replicates in combination with the visual quality control features allow fine-tuning of the peak-calling results to obtain the optimal results for each data set.

A detailed description of the benchmarking data set and a more intense evaluation of Sierra and its parameters can be found, in the Chapters “Benchmark Data Set and Quality Measures” and “Evaluation” of the Additional file 1, respectively. Furthermore, the justification of the default parameters and a guideline for how to recognize noisy data sets alongside with suggestion for parameter choices for noisy data sets are provided.

### Real data set

#### Peak agreement

**Fig. 5:**
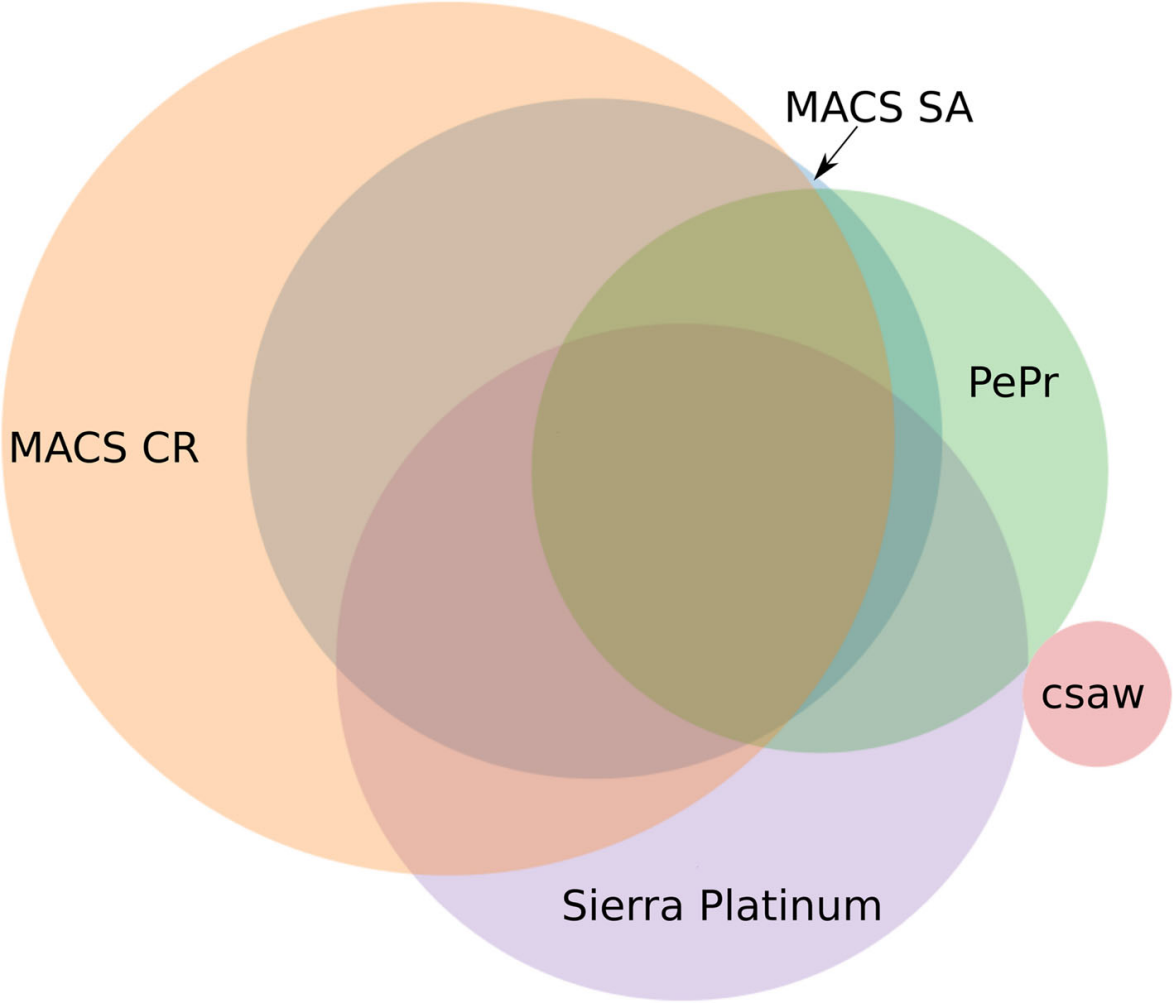
Agreement of the Peak Predictions: The overlap in the peaks predicted by Sierra Platinum (*purple*), MACS-SA (*blue*), MACS-CR (*orange*), PePr (*green*), and csaw (*red*) is shown

The analysis of the agreement of the peaks predicted by Sierra Platinum and by different publicly available peak caller is based on H3K4me3 measurements of all three replicates of BMP4 Trophoblast Cells in the GEO Series GSE16256. We predicted peaks with Sierra Platinum, the MACS-SA and MACS-CR approaches, PePr, and csaw. The overlap of the peak predictions is shown in Fig. 5. A large amount of the peaks are predicted by both MACS approaches, PePr, and Sierra Platinum. Many peaks predicted by Sierra Platinum are also predicted by MACS-CR and MACS-SA. Only csaw does not show a significant overlap with the peak prediction of the other peak caller. The reason for this is, that csaw was designed to make differential peak calls but not peak calls of an experiment with respect to a background.

#### Stem cell marker coverage

We analyzed the stem cell marker coverage and we looked at the genes known to be embryonic stem cell marks, i.e., they are active in embryonic stem cells and their activity is crucial for cell identity and function. We will compare the predicted epigenomic states of the promoters of such markers. The epigenomic state is hereby defined as the collection of peaks for the histone modifications analyzed. Three different epigenomic states will be compared. Firstly, the epigenomic state predicted with Sierra Platinum on the H1 data set. Secondly, the epigenomic state predicted with Sierra Platinum on the ESCs data set. Thirdly, the consolidated epigenomic state of the H1 cell lines downloaded from the NIH Roadmap Epigenomics Webportal of the Washington University (epigenome E003, only H3K4me3, H3K27me3, and H3K9me3; they used the MACS-CR approach). The calculations of the three epigenomic states is described in the supplemental information.

PePr crashed on the H1 and ESCs data set because it could not find any significant window at all. Thus, we cannot compare our results to the epigenetic state generated with PePr. We also run csaw [6] on the H1 data. Even though csaw found a few “up-regulated” peaks, most differential binding events where enriched in the control (“down-regulated” peaks) rather than in the ChIP experiment. This indicates that the results of csaw are very error-prone. In both cases—PePr and csaw—we would argue that due to the high variance of the replicates, it is hard to find windows where the variation between the experiment and the background is much smaller than the variation between the replicates.

We looked at five stem cell markers for embryonic stem cells: SNF2H, BRG1, SSRP1, OCT4, and SNF5 [10]. The peaks generated by Sierra Platinum for the H1 and the ESC data set largely overlap these regions even though the signal was different. In particular, they have very similar peaks for H3K4me3 (light and middle green) but also for H3K9ac (light and dark pink) the agreement of H1 and ESCs peaks is large. Comparing the three marks that we downloaded for E003 to H1 and ESCs, we see this agreement only—if at all—in H3K4me3. We show the results for SNF5 in the following and the results for the remaining markers in the Additional file 1.

##### SNF5

**Fig. 6:**
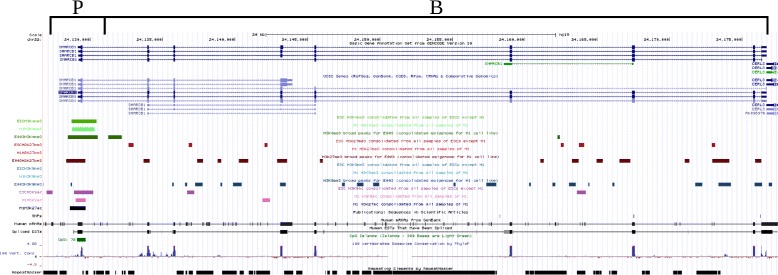
Genomic location of SNF5: The peak-calls are shown below the transcript annotation of SNF5. Peak-calls for the different modifications and data sets are color coded as follows: *green*: H3K4me3, *red*: H3K27me3, *blue*: H3K9me3, *black*: H3K27ac, *purple*: H3K9ac; *light*: H1, medium: ESC, *dark*: E003 (not all combinations exist). The position of the described promoter and gene body are marked with ‘P’ and ‘B’, respectively

Around the main promoter for SNF5, we found a strong activating signal (see Fig. 6). All three activating marks, namely H3K4me3, H3K27ac, and H3K9ac, are present in the epigenomic states of H1 and ESCs if this modification was included in the epigenomic state. Only in the ESCs epigenome there are two H3K27me3 peaks. One peak locates 5’ of the promoter of SNF5 (SMARCB1) and thus, probably does not influence the transcription. The second peak is located nearby the alternative transcription start site and may repress transcription from the corresponding alternative promoter. E003 gives a different picture. The promoter of SNF5 is at least poised according to E003, i.e., there are peaks for H3K4me3 and H3K27me3. There is even a small H3K9me3 peak at the transcription start site. Therefore, one would conclude that this gene is switched off.

##### Overall agreement

**Fig. 7:**
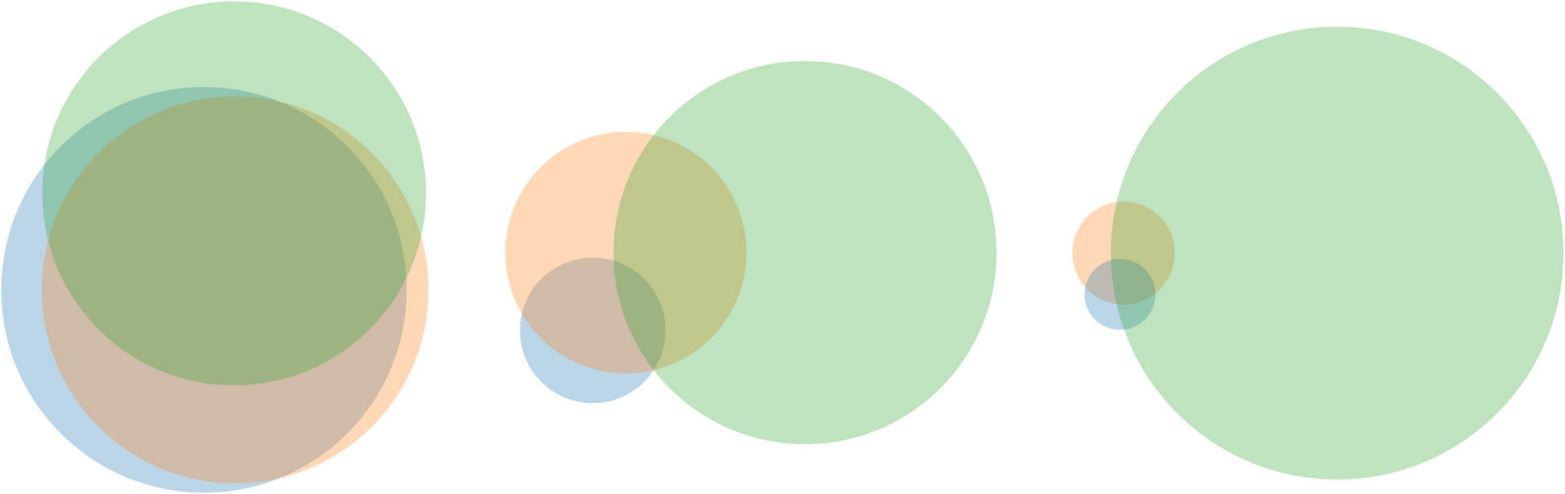
Venn Diagrams for all three modifications showing how often the data sets predict the presence of the mark in the promoters: (*left*) H3k4me3, (*middle*) H3K27me3, (*right*) H3K9me3. Blue circles correspond to H1, orange circles correspond to ESC, and green circles correspond to E003

While the stem cell marker may present a very important class of genes for the cell type studied, the overall agreement in the promoter state indicates whether the observed differences are a global trend or just effects restricted to the chosen example. Therefore, we ask now, how strong the different epigenomic states agree on the association of a promoter with one single modification. We calculated the Venn diagram showing how many promoters are associated with a specific modification in the three data sets (see Fig. 7). In the overlap of all three sets will be therefore all those promoters which are associated with the mark in all three data sets. Association with H3K4me3 shows the strongest agreement. All circles have a similar size and the overlap of all three data sets is large (> 46 %). For H3K27me3 and H3K9me3, this region represents less than 2 % and less than 1 %, respectively. The main reason for this is the large number of promoters associated with this mark in E003 (large green circles in the Venn diagrams). Even though the agreement between H1 and ESC is not strong, it is still stronger than the overlap with E003.

**Fig. 8:**
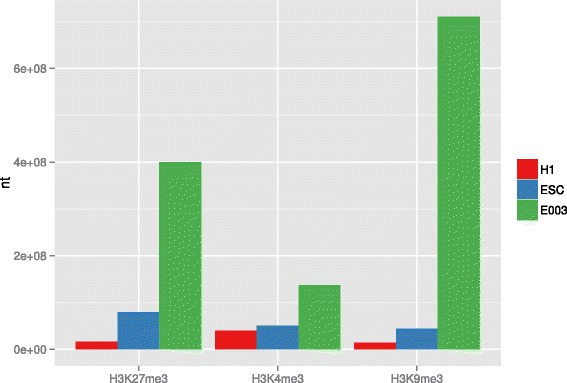
Sum of nucleotides that are covered with peaks of each modification for each data set

We finally investigated whether the excessive amount of predicted peaks by E003 compared to H1 and ESC is a general, genome-wide trend or specific for the promoter regions. As a measurement, we used the total number of nucleotides that are covered with peaks of each modification for each data set (see Fig. 8). Indeed, E003 has a much higher coverage for each of the modifications. The difference for H3K4me3 is the lowest but still, E003 assigns this modification to more nucleotides than H1 and ESC. The effect is most extreme for H3K9me3 in E003.

##### Summary

E003 and H1 are based on the same data and nevertheless predict different peak positions. Given, that the examples we showed are all embryonic stem cell markers which have to be active, one would expect only activating marks. Thus, the Sierra Platinum peak-calls based on both, H1 and ESCs, fit very well the expectation for the shown markers while the E003 peak-calls are suspicious. In particular, the inactivity of SNF5 would be lethal for embryonic stem cells [10]. Also for the other stem cell markers shown here, it is reported that inactivity is lethal [10]. Consequently the active chromatin predicted by Sierra Platinum is more reliable than the may be inactive chromatin or at least with inactive marks speckled chromatin predicted by E003.

In the Chapter “Results” of the Additional file 1, a more detailed comparison of the epigenomes is shown. Besides stem cell marker coverage, we also report the results of our analysis of H3K4me3 and H3K9me3 coverage in the Hox-C and the Hox-D clusters as well as of a promoter region coverage analysis.

### Time and memory consumption

We compared the time and space requirements of the optimized version of Sierra Platinum with those of MACS-CR, MACS-SA, and PePr using real data. For two different sets of window offset and window size, Table 1 lists the runtime and the maximally used memory for all methods. Both, MACS-CR and MACS-SR, reflect only the time and memory used for peak-calling, i.e., of running MACS2. The total time to generate the peaks is longer including additionally the step for combining the input files (MACS-CR) or the output files (MACS-SA), respectively. While for MACS-SA the time for combining the results is negligible, combining the input files for MACS-CR can take very long depending on the permanent storage access speed. In the case of PePr, a complete measurement was not possible since PePr crashed after calculating the candidate windows. Further steps could not be performed since not a single candidate window was found. This also shows that PePr is not robust against noisy data.

**Table 1:** Time and memory consumption for peak-calling the six replicates of H3K4me3 in H1

Method	100nt, 400nt		50nt, 200nt	
	Time (mm:ss)	Max mem (GB)	Time (h:mm:ss)	Max mem (GB)
Sierra platinum	23:16	20.0	48:46	24
PePr	^a^59:15	^a^16.0	^a^4:12:46	^a^21.24
MACS-SA	33:23	1.0	33.41	0.96
MACS-CR	30:47	3.4	30:15	3.26

All results were obtained on the same machine (see Additional file 1 for hardware specification) and with the same input files using a cut-off value of *p*=10^−5^

^a^Elapsed time or maximal memory used until crash

The results show that Sierra Platinum is fastest among all tested methods (window offset = 100 nt, windows size = 400 nt) even though not all steps for MACS-CR and MACS-SA are included in the time and PePr crashed before finishing. Changing the window offset to 50 nt and the window size to 200 nt does not change the runtime or memory consumption of the methods MACS-SA and MACS-CR much. Sierra Platinum uses twice as much time and 20 % more memory. PePr also crashes for these settings after more than 4 hrs.

The memory consumption of Sierra Platinum can not be compared to that of the other methods. For PePr the memory needed is unknown, as it crashed pre-maturely. For MACS-SA, only one replicate is handled at a time, and for MACS-CR all reads are already combined. Also, the memory needs of the pre- and post-processing steps are not included. Finally, Sierra Platinum uses the memory not only for computing the peaks, but also for computing quality information, which increases its memory consumption.

## Discussion

Sierra Platinum is substantially better than the existing peak-calling approaches for replicated ChIP-seq experiments. Our benchmarking results indicate that we are able to find peaks with enough precision to obtain good recalls, low false discovery rates, and positive predictive values on the benchmarking data sets. Even using the default settings, the performance of Sierra Platinum on *noisy* data is superior to the performance of other peak-calling approaches on *noise-free* data. Deleting or down-weighting increases the performance of Sierra Platinum on noisy data reaching the performance on noise-free data. Thus, the method implemented in Sierra Platinum is robust against any kind of noise in the data.

The visual controls implemented in Sierra Platinum enable detecting the source and type of noise and thus allow reducing the influence of noisy replicates by down-weighting or deleting them. Sierra Platinum’s performance is already superior to the other methods for multiple-replicate peak-calling when adapting its parameter settings to those suggested for MACS or PePr. With optimal parameter settings recall *and* positive predictive value can be increased even further. Thus, Sierra Platinum allows high quality peak-calls. Furthermore, by fine-tuning the parameter settings guided by the visual quality controls, the calculations of Sierra Platinum can be adapted to provide optimal results for the given data.We showed that on real data, such as the H1 and ESCs data sets downloaded from the NIH Roadmap Epigenome project [3], peak-calls from Sierra Platinum fit current knowledge reported in literature better than peak-calls produced with MACS-CR. The only other tool able to perform multiple-replicate peak-calling, PePR, was not even able to handle the data but crashed because it could not find any significant window. It is very likely that this is a result of the high variance in the data and its partially low quality. In summary, the strength of Sierra Platinum lies in its robustness against noisy replicates, and in the additional capability to assess their data quality and to reduce the influence of noisy replicates by down-weighting or excluding them.

## Conclusion

Sierra Platinum is a fast and robust multiple-replicate peak-caller. So far, it is the only peak-caller allowing quality control and -steering and therefore leads to better peak-calls compared to current approaches and tools. The procedure and parameters are chosen to produce an optimal result with respect to recall and FDR. Sierra Platinum is robust against noise and thus allows multiple-replicate peak-calling even for replicates not produced by the same lab or study. Alongside with Sierra Platinum, we provide a benchmark data set which allows to compare the performance of peak-callers with respect to specificity and sensitivity. The implementation of the method is optimized such that we only consume as much memory as required to ensure a fast computation of the peak-calls.

## Additional file

Additional file 1 Supplementary information. The supplementary information (pdf file) contains a very detailed description of the method implemented in Sierra Platinum and a description of Sierra Platinum (user interface and infrastructure) and its optimization. Furthermore, we present a very comprehensive benchmark of Sierra Platinum, i.e., a comparison to other peak-callers and peak-calling approaches for replicated ChIP-seq experiments for various conditions and a parameter benchmark for the parameters of Sierra Platinum. We provide derived parameter settings and guidelines for choosing the right parameters based on the quality measurements implemented in Sierra Platinum. The supplementary information contains also a more extensive comparison between the result of Sierra Platinum and MACS-CR on real data. (PDF 3000 kb)

## Abbreviations

ac: Acetylation
bp: Number of base pairs
BRG1: Protein brahma homolog 1
ChIP-seq: Chromatin immunoprecipitation sequencing
DNA: Deoxyribonucleic acid
ESC: Embryonic stem cells
FDR: False discovery rate
GS: Gold standard
H3: histone H3
H3K27ac: Acetylation at histone H3 at the lysine at position 27
H3K27me3: Trimethylation at histone H3 at the lysine at position 27
H3K4me3: trimethylation at histone H3 at the lysine at position 4
H3K9me3: Trimethylation at histone H3 at the lysine at position 9
K4: Lysine (one letter code K) at position 4
MACS-CR: Combining the reads of all replicates and calling peaks using MACS
MACS-SA: Calling peaks of each replicate using MACS and combining the peaks
me3: Trimethylation
nt: Number of nucleotides
OCT4: Octamer-binding protein 4
PCR: Polymerase chain reaction
PPV: Positive predictive value
replicate: Replicated ChIP-seq experiment
SNF2H: Sucrose nonfermenting protein 2 homolog
SNF5: Sucrose nonfermenting, yeast, homolog-like 1
SSRP1: Structure specific recognition protein 1
