# Supplementary material for: Sierra platinum: a fast and robust peak-caller for replicated ChIP-seq experiments with visual quality-control and -steering
Source: BMC Bioinformatics. 2016 Sep 15;17:377. doi: 10.1186/s12859-016-1248-6 (PMC5025614; doi:10.1186/s12859-016-1248-6)
Supplement: Additional file 1 — Supplementary information. The supplementary information (pdf file) contains a very detailed description of the method implemented in Sierra Platinum and a description of Sierra Platinum (user interface and infrastructure) and its optimization. Furthermore, we present a very comprehensive benchmark of Sierra Platinum, i.e., a comparison to other peak-callers and peak-calling approaches for replicated ChIP-seq experiments for various conditions and a parameter benchmark for the parameters of Sierra Platinum. We provide derived parameter settings and guidelines for choosing the right parameters based on the quality measurements implemented in Sierra Platinum. The supplementary information contains also a more extensive comparison between the result of Sierra Platinum and MACS-CR on real data. (PDF 3000 kb) [file 12859_2016_1248_MOESM1_ESM.pdf]

Sierra Platinum: A Fast and Robust Peak-Caller for Replicated ChIP-seq Experiments With Visual Quality-Control and -Steering

Lydia Müller[∘⋈♢△], Daniel Gerighausen[∘∗⋈♡△], Mariam Farman[∘⋈♠], and Dirk Zeckzer[∗⋈♣]
[∘] Bioinformatics Group
[∗] Image and Signal Processing Group
[⋈] Department of Computer Science, Leipzig University, Germany
[♢] Email: lydia@bioinf.uni-leipzig.de
[♡] Email: daniel@bioinf.uni-leipzig.de
[♠] Email: mariam@bioinf.uni-leipzig.de
[♣] Email: zeckzer@informatik.uni-leipzig.de
[△]These authors contributed equally to this work.

Contents

1 Method **4**
1.1 Goal . . . 4
1.2 Overview of the Multi-Replicate Peak-Calling Process . . . 4
1.3 Detailed description of the Multi-Replicate Peak-Calling Process . . . 7
1.3.1 Constructing Windows . . . 7
1.3.2 Joining Windows . . . 7
1.3.3 Computing Mapped Read Quality . . . 7
1.3.4 Computing the Poisson Distribution . . . 8
1.3.5 Computing the Tag Count Distribution . . . 8
1.3.6 Scaling Experiments . . . 9
1.3.7 Computing the Normalized Poisson Distributions . . . 9
1.3.8 Computing Neighborhoods . . . 9
1.3.9 Computing Single Replicate P-Values . . . 10
1.3.10 Converting P- to Q-Values . . . 10
1.3.11 Determining the Significant Windows . . . 11
1.3.12 P-Value Distribution . . . 11
1.3.13 Establishing Pearson's Correlation between Replicates . . . 11
1.3.14 Filtering and Weighting Replicates . . . 13
1.3.15 Computing the Combined P-Value . . . 13
1.3.16 Converting the Combined P-Value into Q-Value . . . 15
1.3.17 Final P-Value Distribution . . . 15
1.3.18 Computing the Agreement between the Multi-Replicate and the Single Replicate Results 15
1.3.19 Computing Narrow Peaks . . . 16
1.3.20 Computing Broad Peaks . . . 16
1.3.21 Computing Peak Quality . . . 16
1.3.22 Storing the Results . . . 16

2 Technical Details and Graphical User Interface **18**
2.1 System . . . 18
2.2 Sierra Platinum Server . . . 18
2.3 Server Configuration File . . . 18
2.4 Sierra Platinum Client . . . 19
2.4.1 Communication with Server . . . 19
2.4.2 Replicates, Parameters, and Starting Computation . . . 19
2.4.2.1 Editing the list of replicates . . . 19
2.4.2.2 Setting parameters . . . 20
2.4.3 Quality Control . . . 20
2.4.4 Correlation Information, Recalculation Parameters, and Restarting Computation . . . 21
2.4.5 Peak Information . . . 21
2.4.6 Quality Information . . . 21
2.4.7 Additional Functionality . . . 21
2.4.7.1 Loading and saving the data mapper . . . 21
2.4.7.2 Export graphics . . . 21

3 Optimization **24**
3.1 Goal . . . 24
3.2 Context and Data Sets . . . 24
3.3 Approach . . . 24
3.4 Optimization of the Multi-Replicate Peak-Calling Process: Overview . . . 26

3.5 Optimization of the Multi-Replicate Peak-Calling Process: Detailed Description . . . 26
3.5.1 Constructing Windows . . . 26
3.5.1.1 Tag Count Parallel . . . 26
3.5.1.2 Chromosome Parallel . . . 26
3.5.1.3 Chunk Parallel . . . 27
3.5.1.3.1 Chunk – Window – Dataset . . . 27
3.5.1.3.2 Chunk – Dataset – Window . . . 27
3.5.1.3.3 Dataset – Chunk – Window . . . 27
3.5.1.3.4 Summary . . . 28
3.5.1.4 Chunk Parallel Coherent . . . 28
3.5.2 Joining Windows . . . 28
3.5.3 Computing Mapped Read Quality . . . 28
3.5.4 Computing the Poisson Distribution . . . 28
3.5.5 Computing the Tag Count Distribution . . . 28
3.5.6 Scaling Experiments . . . 29
3.5.7 Computing the Normalized Poisson Distributions . . . 29
3.5.8 Computing Neighborhoods . . . 29
3.5.9 Computing Single Replicate P-Values . . . 29
3.5.10 Converting P- to Q-Values . . . 30
3.5.11 Determining the Significant Windows . . . 30
3.5.12 P-Value Distribution . . . 30
3.5.13 Establishing Pearson's Correlation between Replicates . . . 30
3.5.14 Filtering and Weighting Replicates . . . 30
3.5.15 Computing the Combined P-Value . . . 31
3.5.16 Converting the Combined P-Value into Q-Value . . . 31
3.5.17 Final P-Value Distribution . . . 31
3.5.18 Computing the Agreement between the Multi-Replicate and Single Replicate Results . . . 31
3.5.19 Computing Narrow Peaks . . . 31
3.5.20 Computing Broad Peaks . . . 31
3.5.21 Computing Peak Quality . . . 31
3.5.21.1 Peak Parallel . . . 31
3.5.21.2 Peak Coherent Parallel . . . 31
3.5.21.2.1 Plain . . . 31
3.5.21.2.2 Space . . . 32
3.5.21.2.3 Broad-Narrow . . . 32
3.5.21.2.4 Smart . . . 32
3.5.21.3 Results . . . 32
3.5.22 Storing the Results . . . 32
3.6 Space Considerations . . . 32
3.7 Results . . . 33

4 Benchmark Data Set and Quality Measures **34**
4.1 Context . . . 34
4.2 State-of-the-Art and Gaps . . . 34
4.3 Goal . . . 34
4.4 Challenges . . . 35
4.5 Benchmarking Data Set Creation . . . 35
4.5.1 Background data . . . 35
4.5.2 Experiment data . . . 35
4.6 Benchmarking Data Sets . . . 36
4.6.1 Replicates Generated . . . 36
4.6.1.1 Noise free . . . 36
4.6.1.2 Wrong signal . . . 36
4.6.1.3 Noisy Signal . . . 36
4.6.1.4 Post-Processing . . . 36
4.6.2 Proposed Benchmarking Data Sets . . . 36
4.7 Statistical Measures for Quality Assessment . . . 37

5 Evaluation **38**
5.1 Introduction . . . 38
5.2 Approach . . . 38
5.3 Sierra Platinum Quality Measures and Visualizations . . . 38
5.3.1 Noise-free data . . . 38
5.3.2 Pure Sequencing Quality . . . 38
5.3.3 Low enrichment . . . 40
5.3.4 Low Sequencing Depth . . . 40
5.3.5 High Sequencing Depth . . . 40
5.3.6 Noisy Data Sets . . . 40
5.3.7 Summary . . . 40
5.4 Parameter Selection . . . 46
5.4.1 How to choose the p-value cutoff . . . 46
5.4.2 How to choose the window size . . . 46
5.4.3 How to choose the window offset . . . 46
5.4.4 Which method for the q-value calculation should be used . . . 47
5.4.5 Summary . . . 47

6 Results **53**
6.1 Data Sets . . . 53
6.1.1 Reference Data . . . 53
6.1.2 Input Data of Sierra Platinum . . . 53
6.1.2.1 Data Set for H1 . . . 53
6.1.2.2 Data Set for ESCs . . . 53
6.2 Peak Calls . . . 53
6.2.1 Peak Calls for H1 . . . 53
6.2.2 Peak-calls for ESCs . . . 53
6.3 Stem cell markers . . . 56
6.3.1 SNF2H & BRG1 . . . 56
6.3.2 SSRP1 . . . 56
6.3.3 OCT4 . . . 56
6.4 Overprediction of H3K4me3 and H3K9me3 in the Hox-C and the Hox-D Clusters . . . 61
6.5 Promoter Analysis . . . 65
6.6 Peak Coverage Analysis . . . 68

Chapter 1

Method

1.1 Goal

The goal of Sierra Platinum is to provide a mathematically sound method for combining the results of replicated experiments. Each replicated experiment consists of the experiment itself and the associated background, the experiment is compared to. Moreover, quality measurements and their visualization are provided to make informed decisions about the quality of the replicates and to select those replicates that should be used or removed.

1.2 Overview of the Multi-Replicate Peak-Calling Process

The multiple-replicate peak-calling process of Sierra Platinum is depicted in Figure 1.1. The input data (left of the figure) consists of all tags of an experiment and its associated background which together form the replicate. The process itself consists of three phases:

(I) *Single replicate p-values:* Windows are constructed and single replicate p-values for each window (represented by pentagons) are computed (Section 1.3.1–1.3.12). This phase is performed for all replicates separately. However, two quality measures compare the windows per replicate to the mean over all replicates for the same window.

(II) *Combined p-values:* For each window, the p-values of the replicates are combined (Section 1.3.13–1.3.15), yielding a combined p-value for each window (represented by the large pentagon).

(III) *Combined peaks:* Suitable narrow and broad peaks and their quality (represented by white peaks) are computed (Section 1.3.16–1.3.21). Further, two quality measures are established and visualized. This phase is computed for the combined windows. Finally, the peaks are exported to files (Section 1.3.22).

Besides computing the peaks, several quality measures are computed. All quality measures and those intermediate results from the method that allow assessing the quality of the input data are visualized. The visualizations support assessing the quality of the input data and making informed decisions. This information can be used to remove or weight replicates (experiments) that are qualitatively weak and to rate the final result. All points, where visualizations are provided and interaction for changing parameters is possible are marked with a magnifying glass in Figure 1.1.

In the following, the 'method' paragraphs describe the computations needed for establishing the peaks, the 'quality measure' paragraphs describe the computations that are used for quality assessment only, and the 'visualization' paragraphs describe the visualizations that support the data assessment.

The steps "Constructing Windows"–"Computing Single Replicate P-Values" are computed only once to establish single replicate p-values. "Converting P- to Q-Values" and "Converting the Combined P-Value into Q-Value" depend on the p to q value conversion method and are computed first with the default method. "Computing the Combined P-Value" is first performed with all replicates included and equally weighted. After examining the results, the weights of the replicates can be changed or replicates can be excluded (Section "Filtering and Weighting Replicates"). Further, the method for converting p to q values can be changed. Then, steps "Converting P- to Q-Values"–"Computing Peak Quality" can be performed again with the new configuration of replicates, weights, and p to q conversion method. This process of changing the configuration and recomputing the combined peaks can be repeated until a convincing configuration was found—i.e., a configuration with optimal quality. Table 1.1 gives an overview of the steps and if they contribute to the method, compute quality measures, or provide visualizations.

Sierra Platinum combines two established methods:

1. The approach of Zhang et al. [30] (implemented in MACS) was adopted for splitting the genome into windows, for calculating the p-values for each window replicate, and for generating narrow and broad peaks.

2. The inverse normal method as presented by Hedges and Olkin [15] and as used by Wright et al. [28] is adapted to combine the p-values of the

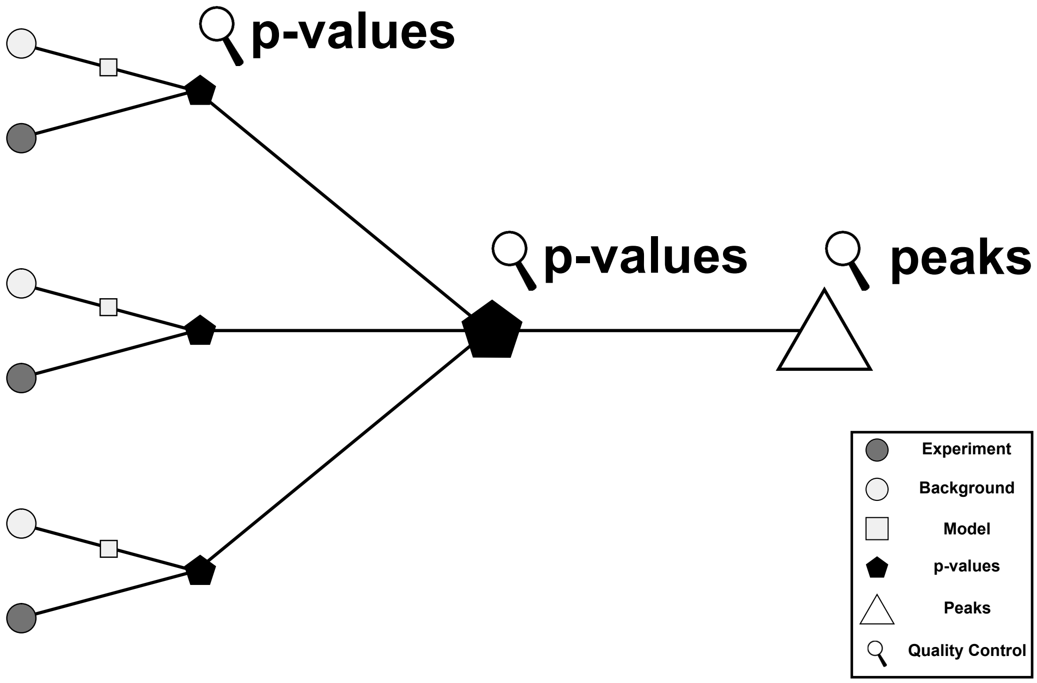

Figure 1.1: Overview of the multiple-replicate peak-calling process. Phase I: Windows are constructed and single replicate p-values for each window are computed (pentagons) Phase II: From the single p-values, combined p-values are computed by combining windows using the inverse normal method (large pentagon). Phase III: Suitable narrow and broad peaks (white triangle) are computed based on the windows' combined p-values. The magnifying glass symbolizes all points, where a visualization-based quality control is included in the peak-calling process.

different replicates for each window into one p-value for each window.

Phase I The steps of this phase are computed for each data set—experiment and background of each replicate—or each replicate separately. However, two quality measures compare the windows per replicate to the mean over all replicates for the same window.

First, the windows are constructed (Section 1.3.1: method). Each window has a start position and a size. All tags overlapping this window are counted. Empty windows are discarded. In principle, this leads to a list of windows. However, to reduce the computation time, the first step is done in parallel on chunks of a certain size. This necessitates a complex data structure, which in turn needs to be transformed into a list (Section 1.3.2: method).

The next step is not needed for performing the peak-calling itself. Computing the mapped read quality serves as a quality assessment of experiment and background of a replicate (Section 1.3.3: quality measure, visualization). It allows to decide, whether or not to use this replicate for the computation of the combined peaks.

Afterwards, the Poisson distribution of the tag counts of the windows is computed (Section 1.3.4: method, quality measure, visualization). Similar to Zhang et al. [30], this serves as a model for computing the single replicate p-values (Section 1.3.9). As a quality estimate, the real tag counts are computed and compared to the theoretical Poisson distribution (Section 1.3.5: method, quality measure, visualization). As in general the amount of used and mapped material differs between experiment and background, the experiments are scaled (Section 1.3.6: method). This allows a better comparison between experiment and background. Based on the scaled experiments, the normalized Poisson distributions are computed (Section 1.3.7: method, visualization).

Next, the 1k, 5k, and 10k neighborhood of each window is determined (Section 1.3.8: method). Now, single replicate p-values are calculated for each window and each replicate based on the global λ of the normalized Poisson distribution and the λ values computed for each neighborhood (Section 1.3.9: method). These p-values determine the peaks of the replicates. To reduce the effect from the correlation between the p-values computed, they are transformed into so-called q-values (Section 1.3.10: method). As the q-values are (un-)corrected p-values, they are called p-values in the subsequent sections. As additional quality measurements, the amount of significant windows (Section 1.3.11: quality measure, visualization) and the p-value distribution (Section 1.3.12: quality measure, visualization) are determined.

Phase II During this phase, the information computed for the replicates is combined. First, the correlation between the replicates is determined (Section 1.3.13: quality measure, visualization). This information is used for adapting the configuration of the replicates performed next. On the one hand, the

Table 1.1: The steps performed by Sierra Platinum: section and name of the step, and whether it is part of the method, a quality measure, or a visualization, respectively.

Section		method	quality measure	visual-ization
	Phase I			
1.3.1	Constructing Windows	✔		
1.3.2	Joining Windows	✔		
1.3.3	Computing Mapped Read Quality		✔	✔
1.3.4	Computing the Poisson Distribution	✔	✔	✔
1.3.5	Computing the Tag Count Distribution	✔	✔	✔
1.3.6	Scaling Experiments	✔		
1.3.7	Computing the Normalized Poisson Distributions	✔	(✔)	✔
1.3.8	Computing Neighborhoods	✔		
1.3.9	Computing Single Replicate P-Values	✔		
1.3.10	Converting P- to Q-Values	✔		
1.3.11	Determining the Significant Windows		✔	✔
1.3.12	P-Value Distribution		✔	✔
	Phase II			
1.3.13	Establishing Pearson's Correlation between Replicates		✔	✔
1.3.14	Filtering and Weighting Replicates			✔
1.3.15	Computing the Combined P-Value	✔		
	Phase III			
1.3.16	Converting the Combined P-Value into Q-Value	✔		
1.3.17	Final P-Value Distribution		✔	✔
1.3.18	Computing the Agreement between the Multi-Replicate and the Single Replicate Results		✔	✔
1.3.19	Computing Narrow Peaks	✔		
1.3.20	Computing Broad Peaks	✔		
1.3.21	Computing Peak Quality		✔	✔
1.3.22	Storing the Results	✔		

replicates need to be correlated to compute justified, combined peaks. On the other hand, correlation is problematic for applying the combination method proposed. Therefore, the configuration has to be corrected for the correlation found.

The replicates are filtered and weighted based on their quality assessment (Section 1.3.14: visualization and interaction). To compute the combined p-values for each window, the inverse normal method is applied (Section 1.3.15: method). During this step, replicates, which are filtered out, are discarded, and the correlation coefficients and weights established previously are applied.

Phase III During this phase, the combined p-values are used for computing additional quality information as well as narrow and broad peaks. The combined p-values are again correlated and therefore converted into q-values (Section 1.3.16: method). As the q-values are (un-)corrected p-values, they are called p-values in the subsequent sections. The resulting p-values are visualized to assess the quality obtained (Section 1.3.17: quality measure, visualization). Further, the agreement between each single replicate and the final combined results is computed and visualized (Section 1.3.18: quality measure, visualization). This quality measure allows to assess the influence of each replicate on the final combined result. Finally, the narrow peaks (Section 1.3.19: method) and the broad peaks (Section 1.3.20: method) of the final combined results are determined together with their quality (Section 1.3.21: quality measure, visualization).

1.3 Detailed description of the Multi-Replicate Peak-Calling Process

1.3.1 Constructing Windows

Method The whole genome is split into overlapping windows of size w with offset o. According to our evaluations (Chapter 5), the window size should be the fragment size used in the experiment, while the window offset should be a quarter of the window size. Most frequently, ChIP-Seq data of histone modifications is fragmented with an average fragment size of 200nt. Therefore, the Sierra Platinum defaults are a window size of $w = 200nt$ and a window offset of $o = 50nt$. However, these parameters are accessible through the graphical user interface (GUI, Section 2.4) and thus can be changed according to the data used.

Each window is compared to the tags obtained from the experiment and the background, respectively. The number of tags overlapping each window is stored separately for experiment and background for each replicate. Sierra Platinum assumes that the data is stored in files that are in bam format and that artifacts (PCR duplicates) were already removed using for example SAMtools [22] or Picard tools [2]. To access the data, the HTSJDK library [1] is used. Counts for experiment and background are calculated during the construction phase of the windows. Windows, which do not overlap with any tag in any data set are removed since this is likely to be an artifact of either too low sequencing depth, unknown genome sequence or highly repetitive sequences to which no tags were mapped (depending on the mapper).

1.3.2 Joining Windows

Method Due to parallelization (Chapter 3), windows are kept in a hierarchical data structure during window construction (Sections 1.3.1 and 3.5.1). However, for the subsequent calculations, a linear data structure is more suitable. Therefore, the hierarchical data structure is flattened into a linear list of windows preserving order by genomic start site of the windows within the chromosomes.

1.3.3 Computing Mapped Read Quality

Quality Measure We provide the quality distribution of all mapped tags passed to Sierra Platinum as a quality control for the user. Therefore, we retrieve the quality as Phred Score for each base of each tag in the provided bam file. The Phred score ranges between 0 and 40 and is calculated as

$$Phred = -10 \cdot log_{10}\left(\frac{p}{1-p}\right) \in [0; 40] \qquad (1.1)$$

where p is the probability that the base call is incorrect. However, using the HTSJDK library [1], we can obtain the base-wise Phred score directly from the data.

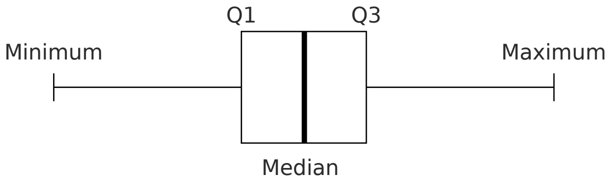

Figure 1.2: Example of a boxplot with lower and upper whisker representing the minimum and maximum in the data.

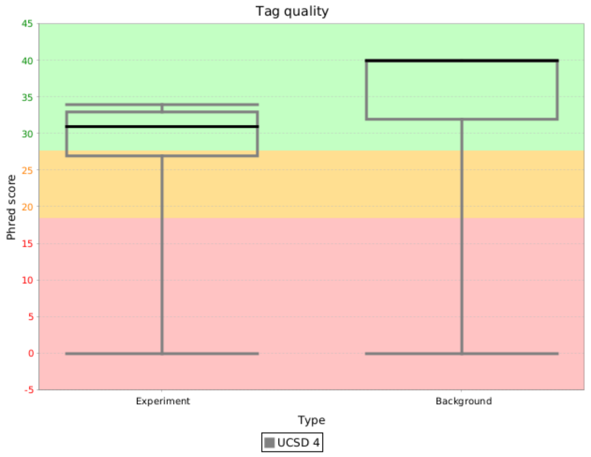

Figure 1.3: Example boxplot figure created by *Sierra Platinum* with the arranged background.

For each data set, we calculate the median, the lower and the upper quartiles, and the minimum and the maximum value of the Phred score distribution.

Visualization The statistics computed are displayed in a boxplot. Boxplots show the median as a line in a box between the lower and upper quartile. This box is extended by the so-called whisker, whose ends represent the lowest and highest value in the data set (Figure 1.2).

The range of Phred-scores is divided into three categories following the approach of *FastQC* [11]. The colors and category ranges for the quality scores used there are also used here. For each replicate, the boxplots for both experiment and background are shown in the same figure for easing their comparison (Figure 1.3).

Ideally, the boxplots for experiment and background are similar, i.e., the read quality was very similar, and most of the bases have a good quality. For real data, this might not be the case. Bad quality of the reads may result in miss-mapped reads and thus, decreases the reliability of the peak-calls based on this data.

If the read quality distributions are very different between experiment and background of the same replicate, one may want to exclude the replicate. In particular, a bad background quality and a good experiment quality can lead to a high rate of false positive peaks. The other way around, a bad experiment quality and a good background quality might be acceptable but comes with the side effect, that it is likely that many peaks are missed.

1.3.4 Computing the Poisson Distribution

Method Following MACS [30], we model the tag distribution of the background with a Poisson distribution and use this as the noise model for the experiment. For each window, we can then ask whether the observed tag counts are significant according to the noise model. We will elaborate more on this in Section 1.3.9.

A Poisson distribution is defined by one parameter λ which describes the mean and the variance of the distribution. Thus, to generate the noise model from the background, we simply calculate the mean of the number of tags in each window for experiment and background of each replicate.

Quality Measure Even though we only need the λ based on the background, we also calculate the λ of the experiment. The latter serves as quality control for the user. Very different means between experiment and background indicate very different library sizes and large differences between experiment and background measurement. It is expected that they do not fit perfectly and we account for this fact by scaling the experiment (see Section 1.3.6). However, scaling may lead to over-estimation of the noise level. Thus, the Poisson distribution of the raw counts (namely the non-scaled counts) of the experiment indicates whether the noise level might be over-estimated.

Visualization To allow the user to observe such issues, we show the Poisson distribution of the raw counts for both data sets, experiment and background, of each replicate as two curves in a line chart (Figure 1.4a). The horizontal axis shows the number of occurrences k of tags in a window and the vertical axis is the probability for each number ablated. Since the number of occurrences can only be integers, the lines between the k-values are only guidelines for a better perception. The red line represents the estimated Poisson distribution for the experiment data and the blue line the estimated Poisson distribution for the background data. It is possible to zoom the line chart to handle also bigger λ values, since their peaks can be far apart from each other.

1.3.5 Computing the Tag Count Distribution

Method, Quality Measure Even though in theory the tags should be Poisson distributed, real data usually does not perfectly fit the theoretical distribution. A small deviation from the theoretical model is acceptable. However, a high deviation or a completely different distribution would mean that the model estimated from the background data is not a good noise model and therefore would result in uninterpretable p-values.

As a quality estimate, we compute the tag count distribution of the real data, i.e., we calculate the relative frequency for each observed tag count. This distribution corresponds to the theoretically estimated Poisson

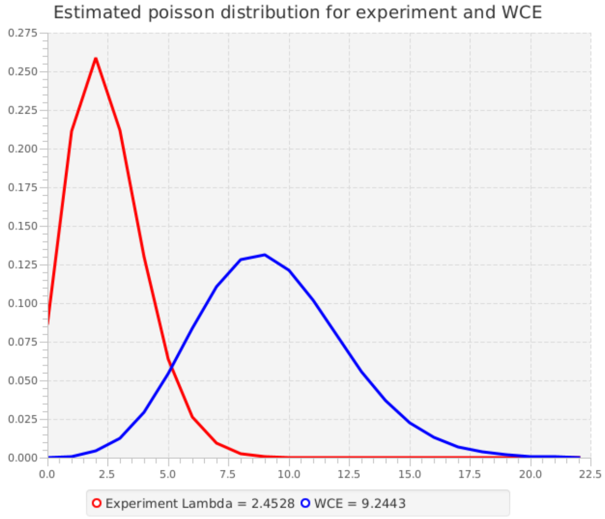

(a) Example of an estimated Poisson distribution for an experiment and the corresponding background.

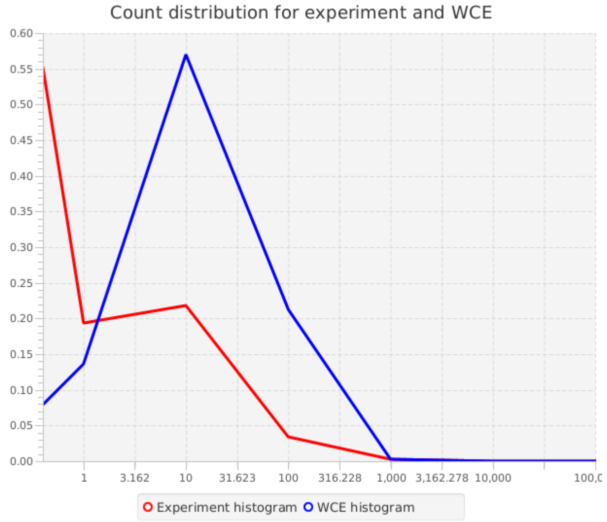

(b) Example of the tag count distribution visualization. The data corresponds to the data used in Figure a.

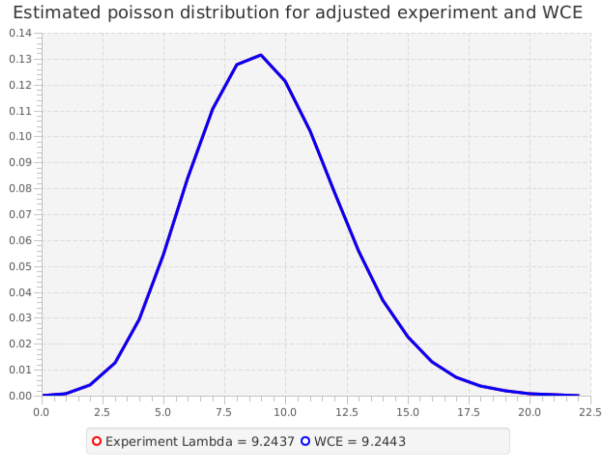

(c) Example of the normalized estimated Poisson distributions. The normalized data was derived from the data used in Figure a.

Figure 1.4: Distribution of tags

distribution. As a measure of fitness, we calculate the least squares difference between the theoretical and the real distribution for both, experiment β_{exp} and background β_{back}.

For each replicate, we use the sum of the least square distances of background and experiment to the corresponding theoretical Poisson distributions as combined least square β for the whole replicate, i.e.:

$$\beta = \beta_{exp} + \beta_{back} \tag{1.2}$$

Since our tool is able to weight replicates during peak-calling, we estimate weights ω based on the least square distance β as follows:

$$\omega = \frac{1}{1+\beta} \tag{1.3}$$

Visualization The tag count distribution is visualized for each replicate using line charts allowing to compare between the tag count distribution and the estimated Poisson distribution. Since real data might contain large outliers, it is necessary to display the number of occurrences of tags k using a logarithmic scale on the horizontal axis. The vertical axis represents the relative frequency k of tags. As for visualizing the Poisson distribution (Section 1.3.4), the experiment is mapped onto red lines and the background data onto blue lines. As shown in Figure 1.4b, the overlap and differences between the tag count distribution and the estimated Poisson distribution can easily be seen. It is clearly visible that the estimated Poisson distributions fit inherently to the data distributions in this example.

1.3.6 Scaling Experiments

Method A good noise model can only be estimated if the amount of used and mapped material are of a comparable amount. For real data, this is usually not the case. Moreover, real data usually suffers from very different library sizes. Therefore, scaling the libraries to the same size is necessary.

In Sierra Platinum, the experiment is scaled such that the library sizes measured as total number of mapped tags are equal. In more detail, we count the total number of mapped tags in the input data separately for experiment and background as t_{exp} and t_{back}, respectively. Then, the scaling factor for the experiment sf is:

$$sf = \frac{t_{back}}{t_{exp}} \tag{1.4}$$

The raw tag counts t_{raw} are then normalized to fit the background counts. The normalized counts t_{norm} are calculated as:

$$t_{norm} = sf \cdot t_{raw} \tag{1.5}$$

Any further reference to counts will refer to the normalized counts from now on.

1.3.7 Computing the Normalized Poisson Distributions

Method The normalization ought scale background and experiment to the same level. As a result also the differences between their respective Poisson distributions should be reduced to a minimum. This can serve as an additional quality check. If the theoretical Poisson distributions estimated from the normalized data are still very different, the normalization was not able to make background and experiment comparable. As a consequence, any p-values estimated based on this data will be spurious and likely to be wrong.

The theoretical Poisson distributions for the normalized data are estimated in the same way as the theoretical Poisson distributions for the raw counts (Section 1.3.4) by simply exchanging the raw counts by the normalized counts.

Visualization It is possible to monitor the results of the scaling and normalization with a visualization of the normalized estimated Poisson distribution in the GUI of *Sierra Platinum*. Since the normalized Poisson distributions are created like their raw Poisson distribution counterparts, the same visualization is used.

An example is shown in Figure 1.4c, where the normalized data was derived from the data used in Figure 1.4a. It is clearly visible that the experiment data was normalized onto the background data since the λ of the background did not change. If for some reason the experiment and the background data are too different, the experiment data can not be normalized that accurately and the experiment and the background lines in the chart do not overlap each other anymore.

1.3.8 Computing Neighborhoods

Method The neighborhoods of sizes 1k, 5k, and 10k are established for each window. These neighborhoods are required during the p-value calculations for the single replicates (Section 1.3.9) to account for local sequence composition biases.

A neighborhood $N_s(w_i)$ of window w_i of size s is the set of windows that overlaps with the interval of size s centered at the mid-point of window w_i. The neighborhood consists of windows on the same chromosome, only.

As discussed in Chapter 3, several options for storing and computing neighborhoods were explored. The final solution does not store neighborhoods, but directly computes the corresponding λ that is then directly used for computing the single replicate p-values (Section 1.3.9). Therefore, each of the neighborhoods is initialized by its range, the window list, and the replicate list. Internally, the index into the window list of the first and of the last neighbor is stored. As the windows are processed sequentially to obtain the corresponding λ-values, the indices are updated for each window. At the same time, the tag count of the window's neighborhood is updated. Dividing the tag count of the neighborhood of window w_i by the number of

windows in this neighborhood yields the $\lambda_s^d(w_i)$ used for the p-value computation:

$$\lambda_s(w_i) = \frac{\sum\limits_{w \in N_s(w_i)} t_w^d}{|N_s(w_i)|} \tag{1.6}$$

where t_w^d is the tag count of window w for data set d (background or experiment of a replicate) and $|N_s(w_i)|$ is the number of windows of the neighborhood $N_s(w_i)$ of window w_i.

1.3.9 Computing Single Replicate P-Values

Method After preparing the experiment counts and the noise distribution, we use this data to calculate for each window and replicate a p-value. The p-value is the probability that one observes an at least as high tag count in random data as observed in the experiment. Hereby, the random data is modeled by the Poisson distribution with mean λ. Thus, the p-value for observing c tags in the experiment is calculated as the reciprocal of the cumulative Poisson distribution with mean λ.

$$\begin{aligned} p &= P(X \geq c, \lambda) \\ &= 1 - P(X < c, \lambda) \\ &= 1 - \sum_{i=0}^{c-1} \frac{\lambda^i \cdot e^{-\lambda}}{i!} \end{aligned} \tag{1.7}$$

It is known that due to biases in the library preparation and the local sequence composition, local estimates of the mean tag count would serve as a better noise model than the global estimate. Therefore, we use the same approach as MACS [30] and calculate the mean tag counts in the 1k, 5k, and 10k neighborhood of each window resulting in λ_{1k}, λ_{5k}, and λ_{10k}, respectively.

The final mean of the Poisson distribution of the noise model is defined as:

$$\lambda = max\{\lambda_{global}, \lambda_{1k}, \lambda_{5k}, \lambda_{10k}\} \tag{1.8}$$

where λ_{global} is the lambda estimated over all windows.

1.3.10 Converting P- to Q-Values

Method Within one replicate and for the final significance test, we repeat the same test for each window to obtain p-values for each window. However, it is well known that this leads to the so-called multiple testing problem. The more often a test is performed, the higher the chance to obtain a false positive result. With other words, the resulting p-values from the tests are too low due to multiple testing.

Several methods exist that allow correcting the p-values thus controlling the false discovery rate. The corrected values are referred to as q-values. In Sierra Platinum, two different methods are implemented: one proposed by Holm-Bonferroni and one proposed by Storey.

Holm-Bonferroni The Holm-Bonferroni correction [17] is a rather conservative method and thus may reduce the number of significant windows dramatically. It assumes that the list of p-values is sorted ascending, i.e., $i < j \rightarrow p_i \leq p_j$.

In Sierra Platinum, we obtain such a list using a parallelized merge sort. The i-th p-value p_i is corrected to q_i by

$$q_i = min(p_i \cdot (N - i), 1) \tag{1.9}$$

where N is the number of tests performed. In our case, the number of tests is equal to the number of windows since we perform one test for each window.

Storey Storey's q-values [25] also calculate the correction factor for the p-values. However, the underlying method is different. It uses the fact that random p-values are uniformly distributed but significance tests usually skew the distribution towards 0 or 1. A good estimate for the rate of false positives $\hat{\pi}_0$ is the height of the uniform distribution while the rate of true positives is the height of the p-value distribution without the uniformly distributed part. Similarly to the Holm-Bonferroni correction, the correction is done stepwise on the sorted list of p-values. Storey's q-value calculation uses a bottom-up approach, i.e., starting with the largest p-value p_N:

$$q_N = \hat{\pi}_0 \cdot p_N \tag{1.10}$$

The subsequent q-values are calculated as follows:

$$q_i = min\left(\frac{N}{i} \cdot \hat{\pi}_0 \cdot p_i, q_{i+1}\right) \tag{1.11}$$

where i is running from $N - 1$ to 1.

To obtain the height of the uniform distribution $\hat{\pi}_0$, Storey proposes several methods. We implemented two of them: 'Storey Simple' and 'Storey Bootstrap'.

Storey Simple The most simple one is to estimate the height $\hat{\pi}_0$ from a representative p-value in the p-value distribution, i.e., 0.5.

Storey Bootstrap One can also fit a cubic spline to the p-value distribution and use the spline to estimate the height of the distribution $\hat{\pi}_0$. We implemented the bootstrap approach that makes fitting the cubic spline more robust [25].

Interaction The p-value correction method can be set using a drop-down-box (Figure 1.8, Section 1.3.14). The available p-value correction methods are 'None', 'Holm-Bonferroni', 'Storey Simple', and 'Storey Bootstrap'. The default is set to 'Holm-Bonferroni' to obtain a conservative correction.

As the q-values are (un-)corrected p-values, we will refer to them as p-values in the subsequent sections.

1.3.11 Determining the Significant Windows

Quality Measure The last quality measurement that Sierra Platinum provides for each replicate is the distribution of the significant windows. More precisely, for each chromosome c and each replicate i, we count the number of significant windows s_i^c:

$$s_i^c = |\{w \in W^c | p_i^w < \hat{p}\}| \tag{1.12}$$

where W^c is the set of all windows of chromosome c, p_i^w is the p-value of window w for replicate i, and $\hat{p}$ is the significance cutoff. Additionally, we calculate the median distribution of the significant windows for each chromosome. In detail, for each chromosome c, we calculate the median significant window $\bar{s^c}$:

$$\bar{s^c} = median\{s_i^c | \forall i \in [1, n]\} \tag{1.13}$$

where n is the number of replicates.

This measurement allows the user to investigate two facts.

Has the current replicate an odd distribution of the significant windows compared to the median distribution? If the overall distribution of significant windows is very different from the median distribution, then the peak-calling of this replicate will not overlap strongly with other replicates and may reduce the quality of the combined peak-calling over all replicates. An odd distribution can have several reasons. One possibility is that the conditions for the experiment of one replicate were very different from the conditions of the other replicates, which might have induced changes in the epigenetic state. Furthermore, it might mean that part of the library preparation or sequencing did not work out as they should. Since this approach is designed to do peak-calling for multiple replicates, i.e., peak-calling for the repeated measurement of the same state, one might prefer to exclude such replicates from peak-calling.

Is there a chromosome with an odd number of significant windows? If the overall distribution is similar to the median but only a few chromosomes diverge from the median, the replicate is suited for the multiple-replicate peak-calling step. However, one might want to perform peak-calling on this single replicate afterwards to investigate the differences in the peaks between this replicate and the multiple-replicate peak-calling on this chromosome.

Visualization The visualization shown in Figure 1.5 provides the necessary information to help the user to deal with the mentioned facts. The amount of significant windows for each chromosome is visualized as a bar chart. The orange bars show the number of significant windows for the replicate and can be compared with the red bars that show the median of significant windows for each chromosome. The user can easily determine if the distribution of significant windows for each replicate is odd by comparing it with the median distribution or detect single chromosomes that deviate from the median.

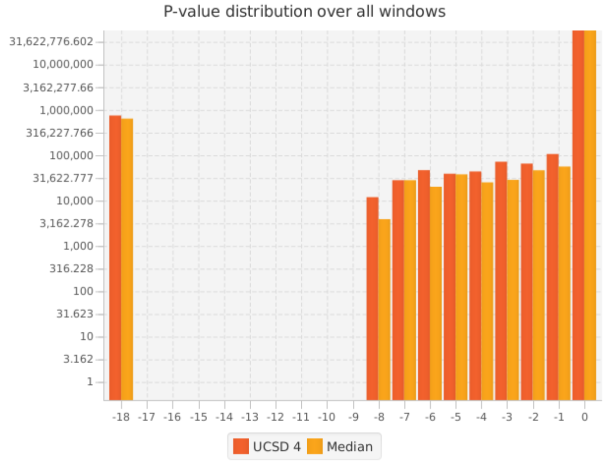

Figure 1.6: Example of the p-value distribution generated during the single peak-calling step. Red: significant windows of one replicate. Orange: median of significant windows over all replicates.

1.3.12 P-Value Distribution

Quality Measure, Visualization For each replicate, we compute the distribution of the p-values and visualize this distribution using a bar chart histogram to check the quality of the single replicate peak-calling step (Figure 1.6). The combined p-values are binned into intervals from 1 to 10^{-18} and visualized as bars. Both x- and y-axis are logarithmically scaled. The numerical method behind the p-value distribution produces values down to 10^{-16}. Smaller values are assigned to bin 10^{-18}.

1.3.13 Establishing Pearson's Correlation between Replicates

Quality Measure Sierra Platinum also provides quality measurements between replicates additionally to those within replicates. The Pearson's correlation between the replicates allows to justify whether the replicates seem to agree on the significance of the windows in consensus (positive correlation).

As a logical consequence from the fact that all replicates measured the same modification (or chromatin bound protein) under comparable conditions and in the same cell line, positive correlations between the replicates are expected.

A positive correlation close to 0 may result from differences in the protocol, the conditions, the sequencing, or the mapping method for the tags. The resulting peak-calls may be biased by this fact and likely contain false negatives and false positives.

Negatively correlated replicates have to be treated with caution for two reasons. (1) Negative correlation indicates that windows in one replicate are significant

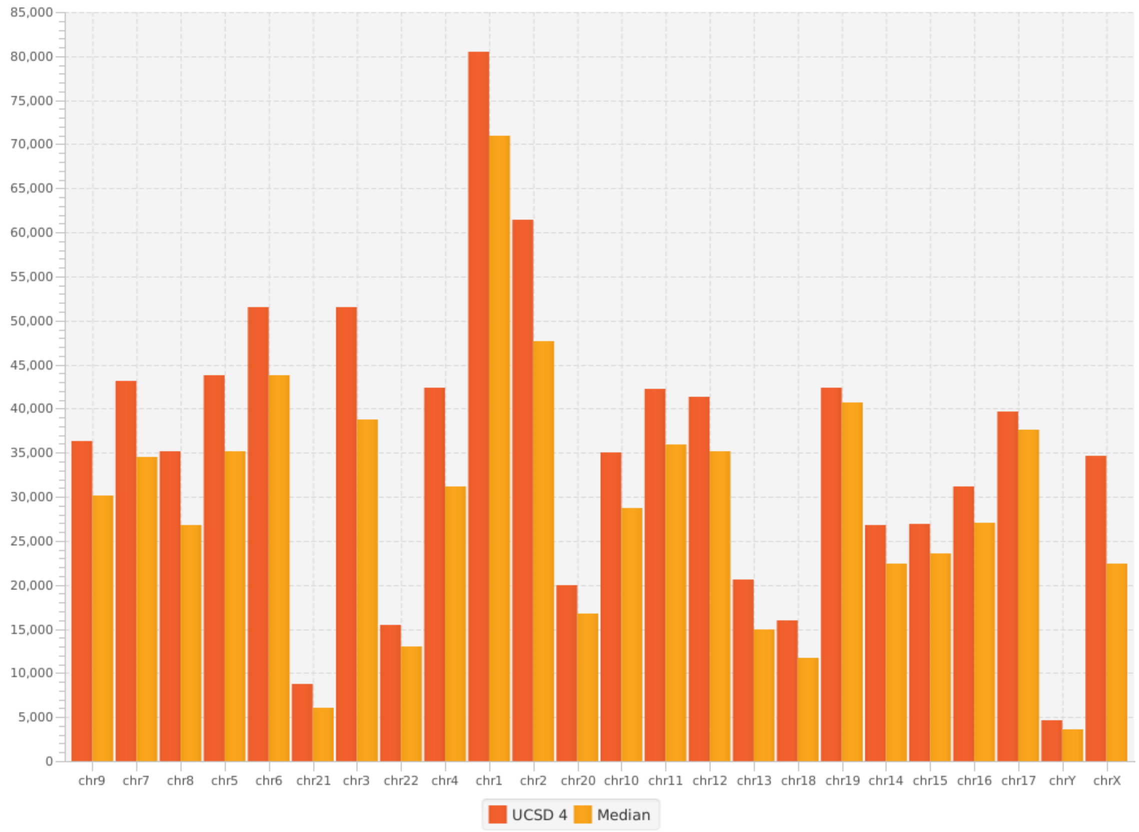

Figure 1.5: Example of significant window distribution over all chromosomes. Red: significant windows of one replicate. Orange: median of significant windows over all replicates.

while in the other replicate they are clearly not significant. This indicates that something went wrong in one of the experimental procedures. (2) For the inverse normal method that we use to combine the p-values of the replicates (see Section 1.3.15), negative correlations are problematic and alter the significance level. Therefore, all replicates used for the multiple-replicate peak-calling have to be positively correlated to each other.

Pearson's correlation assumes that the tested variables are normally distributed. However, p-values are uniformly distributed and are therefore not suited for correlation estimates. We solved this problem by estimating the correlations from the so-called probits instead of from the p-values. Probits are obtained by transforming the p-values with the inverse cumulative standard normal distribution. They are calculated by the inverse normal method, which is also used to combine the p-values of each window of the replicates into one single p-value for each window (see Section 1.3.15).

Let τ, τ' be the vectors of all probits for two replicates and let n_w be the number of windows and thus, also be the length of the vectors τ and τ'. The mean $\bar{\tau}$ of τ is computed as

$$\bar{\tau} = \frac{1}{n_w} \sum_{i=1}^{n_w} \tau_i \tag{1.14}$$

and the standard deviation s_τ of τ is computed as

$$s_\tau = \sqrt{\frac{1}{n_w - 1} \sum_{i=1}^{n_w} (\tau_i - \bar{\tau})^2} \tag{1.15}$$

The mean $\bar{\tau'}$ and the standard deviation $s_{\tau'}$ of τ' are calculated analogously. The Pearson's correlation $\rho_{pearson}(\tau, \tau')$ is thus

$$\rho_{pearson}(\tau, \tau') = \frac{\left(\sum_{i=1}^{n_w} \tau_i \tau_i'\right) - n_w \bar{\tau} \bar{\tau}'}{(n_w - 1) s_\tau s_{\tau'}} \tag{1.16}$$

Visualization The correlations between all replicates are visualized in a heatmap (Figure 1.7). Each cell in the heatmap describes the strength of the correlation between the corresponding column and row. Positive correlations are encoded with red and negative correlations with blue, respectively. The strength of the correlation is mapped to the saturation value, using the *HSB* color model.

$$color_{i,j} = \begin{cases} \text{HSB}(0, c_{i,j}, 1.0), & \text{if } c_{i,j} \geq 0 \\ \text{HSB}(240, (-1 * c_{i,j}), 1.0), & \text{if } c_{i,j} < 0 \end{cases} \tag{1.17}$$

In the GUI of Sierra Platinum, it is possible to see the strength of the correlation for each cell by a tool tip. With this visualization it is easy to see how the replicates are correlated to each other and the user can recognize replicates that are problematic for the

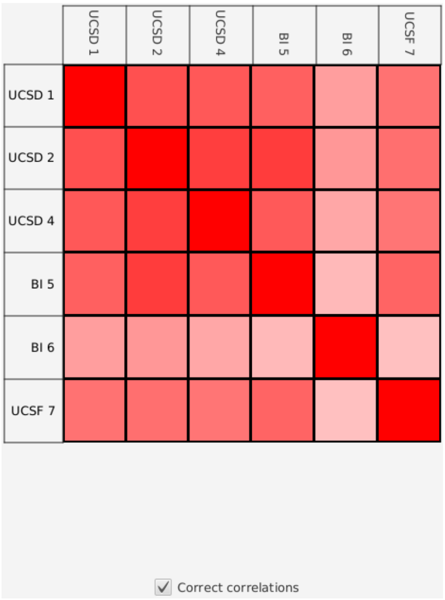

Figure 1.7: Top: heatmap of the Pearson's correlation between the replicates. Bottom: checkbox for enabling or disabling the correlation correction while computing the combined p-value. If the checkbox is checked, $\hat{\rho}^*$ is computed as described in Section 1.3.15, otherwise $\hat{\rho}^*$ is set to 0.

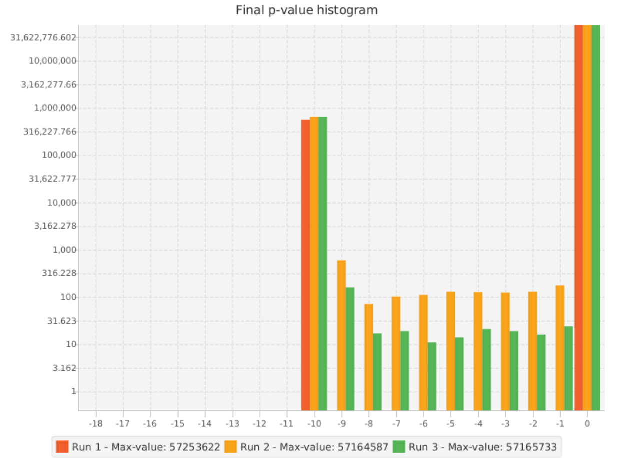

Figure 1.9: Comparison of the final p-value distributions with different weight settings. Each run is assigned a different color according to the color legend below the figure. The p-values of the bins are mapped to the x-axis while the amount of windows having the respective p-value are mapped to the y-axis. A logarithmic scale is used in both cases. Here, three different runs are shown assigned to the colors red (run 1), orange (run 2), and green (run 3). From the p-value distribution alone, run 1 would be preferred over run 3 over run 2.

p-value combination step (see also Sections 1.3.14 and 2.4.4 describing how to enable/disable replicates and how to set weights).

1.3.14 Filtering and Weighting Replicates

Visualization and Interaction Sierra Platinum uses several parameters that influence the computation of the combined peaks. First of all, the user can decide whether or not to use the correlation correction based on Equation 1.22 (Section 1.3.15). If the correlation correction is disabled using the checkbox shown in Figure 1.7, $\hat{\rho}^*$ is set to 0. Moreover, the user can decide whether or not to use a replicate (right column of Figure 1.8). Further, she can assign a weight to each active replicate (middle column of Figure 1.8) or disable weights altogether (weight checkbox in the first row of Figure 1.8). Finally, she can decide whether or not to compute the quality of the peaks (checkbox at the bottom of Figure 1.8; for a description please see Section 1.3.21).

During the initial run, default parameters are used: correlation correction is enabled, all replicates are enabled, the p-value correction method is set to "Holm-Bonferroni", and the weights are set to the ω values of each replicate (Equation 1.3, Section 1.3.5). Further, the peak quality computation is computed by default.

These parameters can and should be changed based on the results of the initial run and the second part of the computation (Section 1.3.15–1.3.22) should be performed again (see also Section 2.4.4). The single replicate peak-calling steps from the first part are not influenced by these parameters and thus this part does not have to be recomputed again. The results of these improved runs can again be analyzed using a histogram (shown in Figure 1.9) to compare the p-value distributions for different parameters settings. The color of each bar encodes a run with a specific parameter setting. As it is not advisable to use more than 8 different colors [26], only the results for the last 8 runs are provided. The results of each run are also stored separately by adding the number of the run to the file name (Section 1.3.22).

1.3.15 Computing the Combined P-Value

Method While MACS would generate a peak list using the calculated p-value at this point, we first apply the inverse normal method to calculate the combined p-value and then generate the peak list based on the combined p-value.

Let p_i be the p-value for replicate i. To calculate the combined p-value, the p-values p_i of all replicates are transformed into probits τ_i using the inverse cumulative standard normal distribution Φ^{-1}:

$$\tau_i = \Phi^{-1}(p_i) \tag{1.18}$$

Since the p-values p_i are uniformly distributed for each window, the respective probits are normally distributed with mean 0 and standard deviation 1 due to the transformation. This step is done for each window and each replicate.

For each window, the combined probit is calculated based on the probits of the replicates. In the simplest

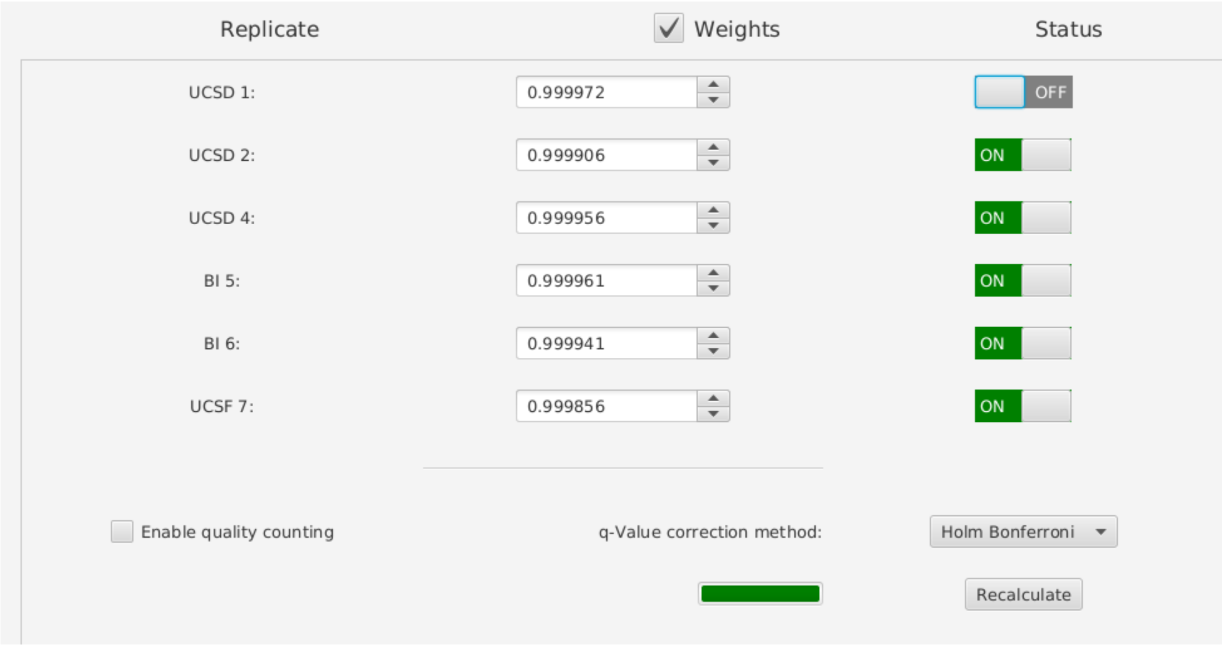

Figure 1.8: Overview of the options to weight replicates provided by the GUI of Sierra Platinum. The upper part contains one row per replicate showing the replicate identifier (left column), the assigned weight (middle column), and whether the replicate is used (ON) or not (OFF, right column). The weight checkbox on top of the middle column allows for disabling weights altogether (if unchecked). The lower part contains a checkbox that allows to enable or disable the computation of the quality of the peaks (Section 1.3.21) and a drop-down-box that allows to select the p-value correction method (Sections 1.3.10 and 1.3.16). Pressing the button (bottom right) starts the recomputation.

case, the replicates are not correlated. Then, all replicates have equal weight and the combined test statistic $\bar{\tau}$ for the n_r replicates is computed as:

$$\bar{\tau} = \frac{1}{\sqrt{n_r}} \cdot \sum_{i=1}^{n_r} \tau_i \tag{1.19}$$

Again, the test statistic $\bar{\tau}$ follows a standard normal distribution.

The corresponding combined p-value p is calculated based on the cumulative standard normal distribution Φ: the p-value is the one-sided, left cumulative probability calculated using the normal distribution with mean 0 and standard deviation 1:

$$p = \Phi(\bar{\tau}) \tag{1.20}$$

However, the replicates and thus the probits are expected to be correlated (see Section 1.3.13 for more details). Therefore, we use a weighted version of the inverse normal method based on the extension proposed by Hartung [14] that can cope with correlations. Therefore, we assigned weights to each replicate to be able to down-weight replicates that are of lower quality (see Section 1.3.5).

According to Hartung [14], the approximated correlation $\hat{\rho}$ between the probits for n_r replicates of a window is

$$\hat{\rho} = 1 - s_\tau^2 \tag{1.21}$$

where s_τ is calculated as given by Equation 1.15.

The correlation estimate $\hat{\rho}^*$, which will be used for the calculation of the combined probit is then calculated as

$$\hat{\rho}^* = max\left\{-\frac{1}{n_r - 1}, \hat{\rho}\right\} \tag{1.22}$$

For better readability of the equation to calculate the combined probit, we define the sum of weights

$$\omega = \sum_{i=1}^{n_r} \omega_i \tag{1.23}$$

and the sum of the squares of the weights

$$\hat{\omega} = \sum_{i=1}^{n_r} \omega_i^2 \tag{1.24}$$

whereby ω_i is the weight for the i-th replicate. The default for Sierra Platinum is to use ω_i of replicate i according to Equation 1.3 (Section 1.3.5).

The resulting combined probit is calculated as

$$\tau = \frac{\sum_{i=1}^{n_r} \omega_i \tau_i}{\sqrt{\hat{\omega} + [\omega^2 - \hat{\omega}][\hat{\rho}^* + \kappa\sqrt{\frac{2}{n_r+1}}(1 - \hat{\rho}^*)]}} \tag{1.25}$$

The parameter κ controls the significance level of the p-value as calculated in Equation 1.20. Hartung [14] experimentally estimated two values for κ from which one should be chosen. In Sierra Platinum, we use the first value proposed, i.e., $\kappa = 0.2$.

Both values of κ described control the significance level well for positive correlations independent of the number of replicates and the variance of the weights. Negative correlations, however, are problematic. In particular in combination with a large variance of the weights, the actual significance level is higher than the one calculated with Equation 1.20. This would lead to false positive peaks.

In the case of multiple-replicate peak-calling, we combine biological and/or technical replicates of the measurement of the same chromatin bound protein under approximately the same conditions in the same cell type or at least in similar cell types. Thus, we expect that the replicates correlate positively (or at least not negatively) with each other. Therefore, negative correlation indicates that the replicates do not fit together and that they may result from an error in the experimental protocol. As a consequence, one would exclude those replicates from peak-calling. Hence, the chosen value of κ is suitable for the use in Sierra Platinum.

Sierra Platinum can also be used for peak-calling single replicates. In this case, the inverse normal method step is skipped and the final p-value is identified with the p-value of the replicate. All other steps are not affected by the number of replicates.

1.3.16 Converting the Combined P-Value into Q-Value

Method Similar to the conversion of the single replicate p-values into q-values (Section 1.3.10), we convert the combined, multiple-replicate p-value into a q-value for each window. The methods described in Section 1.3.10 are applied for the conversion and the resulting q-values are used in the subsequent computations.

Interaction The p-value correction method can be set using a drop-down-box (Figure 1.8, Section 1.3.14). The available p-value correction methods are 'None', 'Holm-Bonferroni', 'Storey Simple', and 'Storey Bootstrap'. The default is set to 'Holm-Bonferroni' to obtain a conservative correction.

As the combined q-values are (un-)corrected combined p-values, we will refer to them as p-values in the subsequent sections.

1.3.17 Final P-Value Distribution

Quality Measure, Visualization The result of the combination of the p-values is shown in Figure 1.10. The combined p-values are binned into intervals from 1 to 10^{-18} and visualized as bars. Both x- and y-axis are logarithmically scaled. The numerical method behind the p-value distribution produces values down to 10^{-16}. Smaller values are assigned to bin 10^{-17} for values of $\bar{\tau} > -20$, while values of $\bar{\tau} <= -20$ are assigned to bin 10^{-18}.

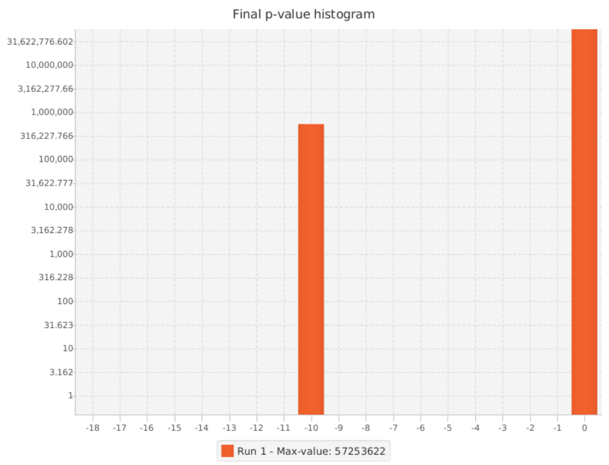

Figure 1.10: Example of the combined p-value distribution.

1.3.18 Computing the Agreement between the Multi-Replicate and the Single Replicate Results

Quality Measure As a further quality measurement, Sierra Platinum calculates the agreement between the multiple-replicate result and the single replicate results. More precisely, it calculates the fractions of the significant windows according to the combined p-value that are also significant in the different replicates. Let C be the set of windows that are significant according to the combined p-value, i.e.,

$$C = \{w \in W | p^w < \hat{p}\} \tag{1.26}$$

where W is the set of all windows, p^w is the combined p-value of window w, and $\hat{p}$ is the significance cutoff.

Analogously, R_i is the set of windows that are significant according to the p-value for replicate i:

$$R_i = \{w \in W | p_i^w < \hat{p}_i\} \tag{1.27}$$

where p_i^w is the p-value of replicate i for window w, and $\hat{p}_i$ is the significance cutoff of replicate i. The agreement between the multiple-replicate result and the result of replicate i is thus

$$a_i = \frac{|C \cap R_i|}{|C|} \tag{1.28}$$

Visualization This mutual agreement is visualized using a bar chart (Figure 1.11a). Each bar represents a replicate and the y-axis gives the percentage of agreement with the multiple-replicate peak-calling result.

This quality measurement eases assessing if the replicates agree with each other and thus with the combined results or if one replicate contributes much less than the other replicates to the final result. A high agreement of all replicates with the multiple-replicate result shows that the replicates themselves agree in their peak-calls.

Again, for multiple runs, the overlap bar chart (Figure 1.11b) is extended with the overlap information of each run.

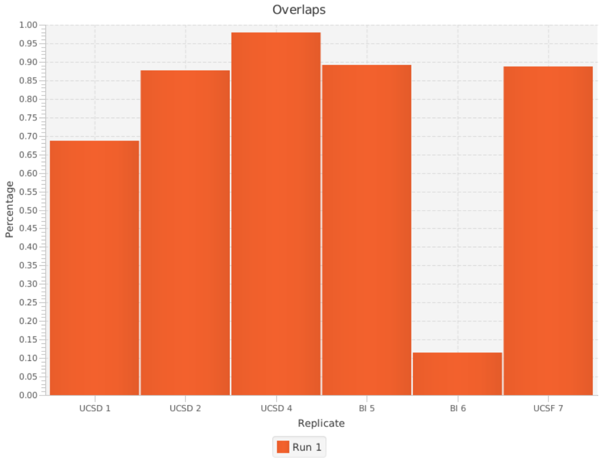

(a) Example of the overlap visualization between all replicates for a single run. The x-axis shows the replicate number while the percentage of overlap of the respective replicate is mapped onto the y-axis.

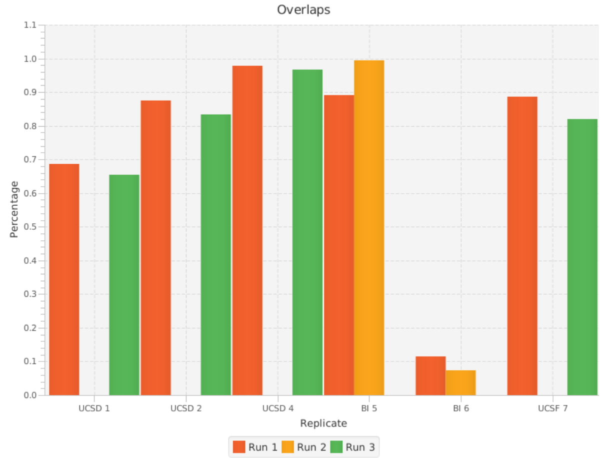

(b) Comparison of the overlap of the peaks between the replicates for multiple runs. During the first run (red) all replicates were used while during run 2 (orange) only replicates 3 and 4, and during run 3 (green) only replicates 1, 2, 3, and 6 were used. While for run 3 the overlap of the individual replicates is more or less balanced, for run 2 this is not the case: replicate 4 overlaps more than 90% with the final result while replicate 5 overlaps less than 35% with the final result.

Figure 1.11: Comparison of the overlap of the peaks between the replicates.

1.3.19 Computing Narrow Peaks

Method We adopted the computation of the narrow peaks from MACS [30]. Significant windows overlapping in their genomic position are merged into the same peak. A window is significant if the combined p-value of this window does not exceed the user-defined significance cutoff. Sierra Platinum reports the lowest combined p-value of the combined p-values of the windows contributing to the peak.

Narrow peaks should be calculated for those modifications known to produce very sharp peaks such as H3K4me3.

1.3.20 Computing Broad Peaks

Method It was found that many histone modifications form broad domains of consecutive modified nucleosomes. Therefore, we also included the computation of broad peaks into Sierra Platinum and again adopted the procedure from MACS [30].

If two peaks are less than two window sizes apart from each other, then they are joined into the same broad peak. This copes with the fact that ChIP-seq is a measurement of a population signal rather than a single cell protocol and that the epigenetic state is controlled by stochastic processes. Thus, at the time of measurement, nucleosomes might be completely unmodified or unmodified in many cells of the population even though they are usually modified. This results in gaps between the peaks. These gaps can also be an artifact of the experimental or computational method. In both cases, one can close the gap computing broad peaks.

1.3.21 Computing Peak Quality

Quality Measure Finally, Sierra Platinum provides a last quality control: the read quality within the peaks. Therefore, we calculate the median read quality for each peak (which is also exported together with the peaks, Section 1.3.22).

Visualization For each replicate, Sierra Platinum again provides two boxplots for the median peak quality distribution in the experiment and in the background similar to those introduced in Section 1.3.3. As shown in Figure 1.12, the GUI of Sierra Platinum provides an overview over all data sets. The whiskers of the boxplots represent the minimum and maximum value of the data set and the background of the plot shows the different quality levels thus assisting the user to interpret the boxplots.

Replicates with a very low median peak quality distribution might be excluded since they only contribute with low quality—and thus suspiciously—data to the final result. Further, strong differences between the median peak quality distributions of experiment and background indicate suspicious results. As detailed analyses show, such replicates should be removed from the analysis (cf. Chapter 5).

1.3.22 Storing the Results

After each run of the multiple-replicate peak-calling process the broad and narrow peaks are exported and saved as *bed* or *csv* files with the p-value as the score field on the server Additionally, it is possible to export the peaks on the client using the GUI of Sierra Platinum.

Sierra Platinum uses *Google GSON* [8] for all data storage. It is usually faster and more robust than the previously used, dated object serialization provided by Java. For efficiency reasons, all files are compressed.

More information is provided in Section 2.4.

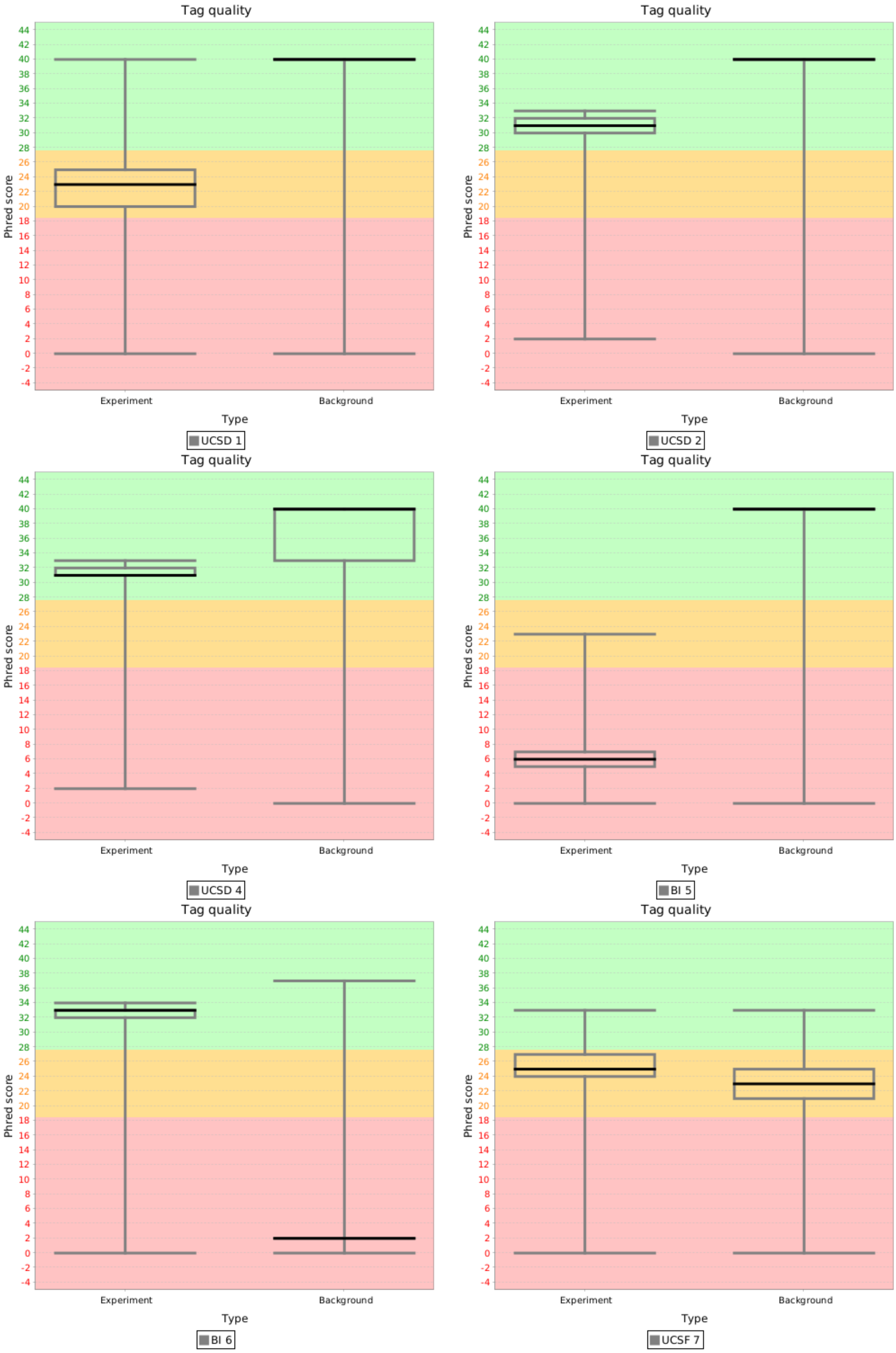

Figure 1.12: Final quality boxplots for experiment and background of 6 replicates.

Chapter 2

Technical Details and Graphical User Interface

2.1 System

Sierra Platinum is completely written in JAVA 8 and uses JavaFX as API for the GUI. Therefore, it is possible to run Sierra Platinum on Windows, Linux, and Mac. The libraries used are Apache Commons IO [3], Apache Commons Logging [4], Apache Commons Math [5], Apache Commons Net [6], Apache Commons VFS [7], HTSJDK [1], JFreeChart [9], Gson [8], and JSch [10].

Choosing Java supports the generation of graphical user interfaces in a straight forward way. Moreover, its language concept based on software engineering principles supports maintainability.

While the computational requirements could be substantially optimized (Chapter 3), they are still high and require a powerful workstation or a server. Since the input data for peak-calling process can be very large, the calculation needs performant workstations that are more powerful than current standard desktop computers. Therefore, the program is split into two parts:

Sierra Platinum Server: performs the peak-calling itself. It takes its parameters from the client and transfers the result to the client. The server is normally run on high-performance machines with sufficient resources, i.e., CPUs with several cores, a large amount of memory, and a high I/O bandwidth.

Sierra Platinum Client: the GUI that allows the user to select the data to use (replicate data sets), to set and adjust the parameters, to assess the results, and to export the results on the users's client. The client can be run on almost any current standard desktop computer.

Additionally, Sierra Platinum can be used in batch mode from a command line. To do so, the user has to create a configuration file for the server. This configuration file can be created with the client by selecting all replicates within the GUI and exporting the client configuration. This can be useful, if, e.g., no direct server connection is possible because of security policies in the lab environment. In principle, a skilled user can create the configuration files manually since they are gzipped JSON files. An example of the configuration is provided in the Section 2.3. With this configuration file, it possible to run the server in batch mode without the need of a client connection. The server will then read all the input data, perform the computations export all results, and then terminate. The user can import the results as a datamapper in the client and then check the results offline, that is, without any server running.

2.2 Sierra Platinum Server

When starting the server, the user can select the maximum number of threads, which are used during IO intensive operations. After every calculation, the server exports the results in bed and csv format to disk. Additionally, it stores the current data mapper and a log file with all information about the latest calculation.

The server can be started in server or in batch mode. In server mode, it accepts connections from clients. When the server is running a job, it is sending progress information to each client. Only one job can be run at a time, due to the large amount of resources needed. Therefore, the server is locked while performing the job, meaning, that it does not accept any additional jobs.

Furthermore, it is possible to run the server in batch mode by providing a previously created job configuration file. In batch mode, the server will compute all results and terminate afterwards.

2.3 Server Configuration File

The server configuration file for batch mode can be exported from the client as described in Section 2.4.7.1. Since the configuration file is a gzipped JSON file, it is also possible to create it manually for automated pipelines. The syntax (format) of the file is given by the following example (white space and line breaks added for readability purposes):

```
{"replicates":
 [{"experiment":"expA.bam",
   "background":"backA.bam",
   "name":"ReplicateA"},
```

```
  {"experiment":"expB.bam",
   "background":"backB.bam",
   "name":"ReplicateB"}],
 "windowsize":200,
 "offset":50,
 "pvaluecutoff":1.0E-5,
 "peakmode":false,
 "numCores":4,
 "jobName":"Example"
}
```

The first seven lines show, how an example with two replicates is generated. The keyword “replicates” is followed by a list (between ‘[’ and ‘]’ of replicates. Each replicate is delimited by the curly brackets and contains three fields: the “experiment” file name (e.g., “expA.bam”), the “background” file name (e.g., “backA.bam”), and the replicate “name” (e.g., “ReplicateA”). Furthermore, the parameters have to be given: the window size, the offset, and the p-value cutoff. The variable ’peakmode’ is only for internal use and should be always ’false’ if the configuration file is created manually. The “numcores” entry determines the maximal number of threads used for the computation. Finally, the “jobName” describes the file prefix which is added to the output files. After creating the configuration file, all white space (including tabs and line breaks) has to be removed to create a JSON file containing exactly one line, and the file has to be compressed with gzip.

2.4 Sierra Platinum Client

The client lets the user interact with the server for selecting the input data of the peak-calling process and for adjusting the parameters. After the calculation, the client visualizes the quality control steps allowing the user to assess the quality of the replicates and of the resulting peaks.

2.4.1 Communication with Server

First, the Sierra Platinum Client is connected to a server using the connection dialog (Figure 2.1). If the calculation should be performed locally, since the data set is small or because the local computer has enough resources, the client can create it’s own instance of the server (‘Start local server’ checkbox). Besides the name of the server (‘Host’), two ports are provided: the ‘Serverport’ is the port on which the server is listening to connections and commands from potential clients, while the ‘Clientport’ is the port on which the client is listening to data received from the server. In principle, different clients on the same computer could use different ‘Clientports’ connecting to different servers. Moreover, it is possible to connect more than one client to the server and to secure the server with a password since it is possible to cancel jobs in the GUI. After the calculation, the server sends a data mapper object to each connected client and the client presents the data. Furthermore, it is possible to pull the data mapper

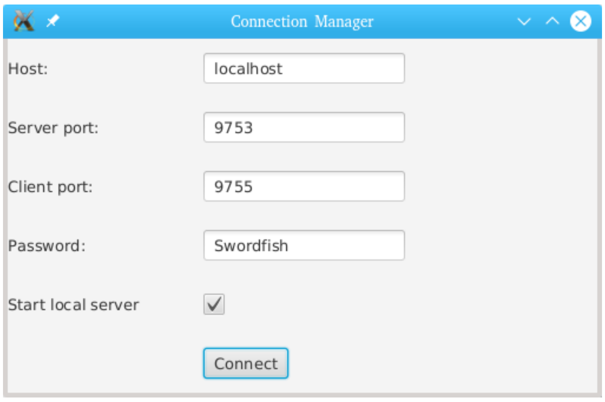

Figure 2.1: The server connection window of Sierra Platinum.

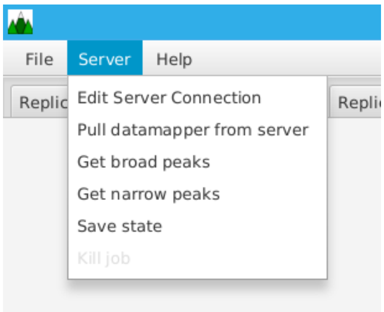

Figure 2.2: The Server Menu

from the server if the client was not connected to the server when the server finished the job.

The connection dialog is opened automatically after starting the client. Further, a new connection can be opened any time using the Menu entry ‘Server → Edit Server Connection’ (Figure 2.2).

2.4.2 Replicates, Parameters, and Starting Computation

To call peaks for a set of replicates, first a list of replicates is created followed by setting the relevant parameters for the process (Figure 2.3). The complete settings—list of replicates and parameter settings—can be saved to file using the menu ‘File → Config Management → Save config’. Alternatively, the settings can be loaded by using ‘File → Config Management → Load config’.

Finally, the process is started by pressing the ‘Start’ button (Figure 2.3). The progress of the computation is shown by the progress bar to the right of the ‘Start’ button.

2.4.2.1 Editing the list of replicates

The user adds replicates using the ‘Add replicate button’ (Figure 2.3). For each of the added replicates, the file associated to the experiment and the file associated to the background of the replicate are selected on the local computer or on the server computer by using a file system browser [7]. A context sensitive menu in the main window allows to change the files associated

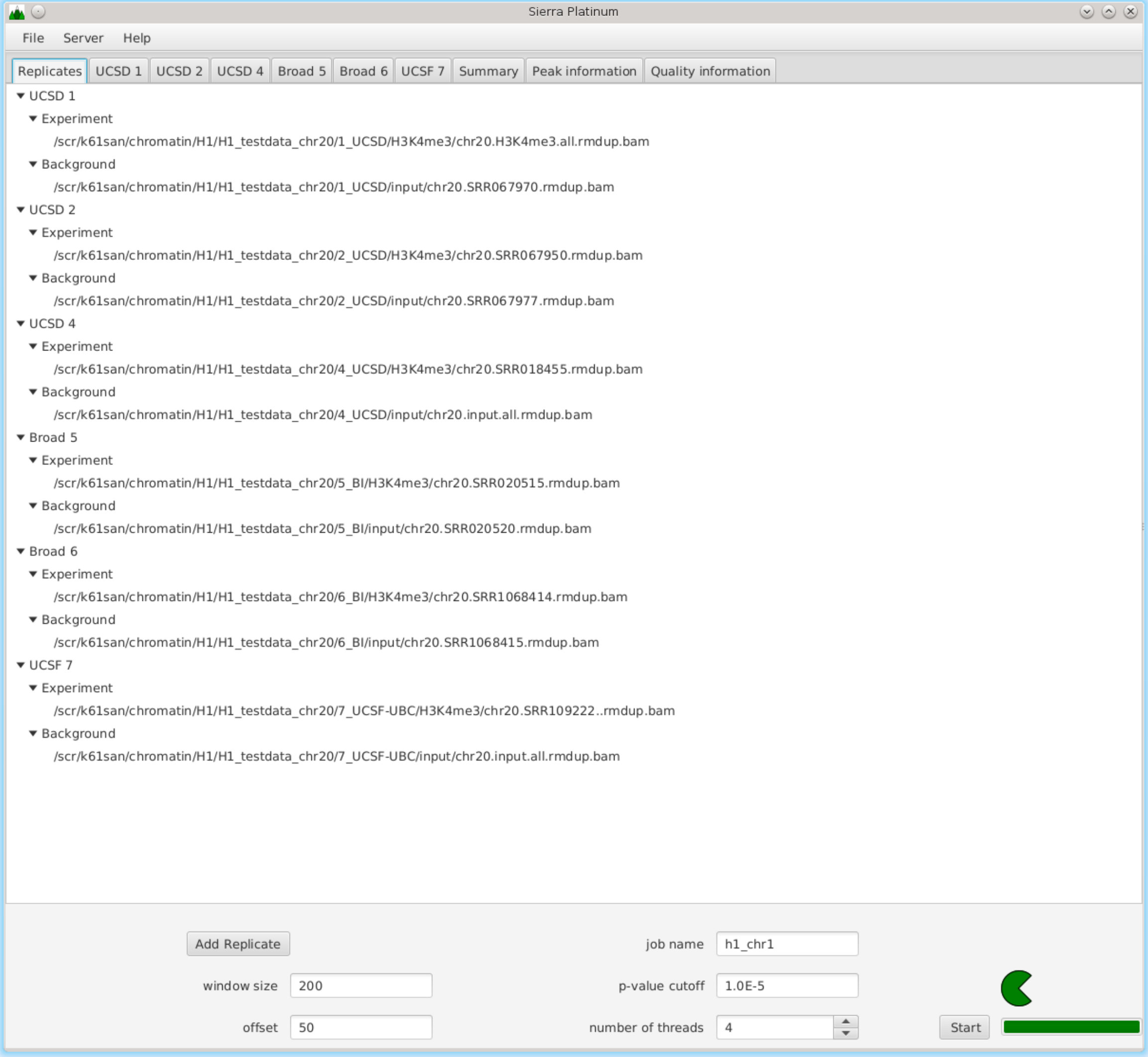

Figure 2.3: The settings-tab in the GUI of Sierra Platinum. In the large, middle window, the replicate information is shown. For each replicate, the file names for experiment and background are given. In the lower part, the 'Add Replicate' button allows adding additional replicates. Below this button, the parameters for the 'window size' and the 'window offset' can be changed. Further, the 'Job name', the 'p-value cutoff', and the 'number of threads' can be assigned. Finally, to the right, the 'Start' button allows starting a computation and the progress bar to the right of this button shows the progress of the computation. A context sensitive menu in the main window allows to change the files associated with the replicates' experiment and background and to delete replicates.

with the replicates' experiment and background and to delete replicates.

2.4.2.2 Setting parameters

The following settings for the current computation can be adjusted (Figure 2.3): 'window size' and 'offset' (Section 1.3.1), 'p-value cutoff' (Sections 1.3.11, 1.3.18, 1.3.19), and the 'number of threads' that should be used for computation. Further, the 'job name' can be set. The job name is used for assigning names to the files used for the information exported.

2.4.3 Quality Control

After computation, for each replicate, a 'Replicate' tab is created showing all relevant information computed for this replicate (Figure 2.4):

- the estimated Poisson distribution for experiment and background (Figure 2.4, top left; Section 1.3.4, Figure 1.4a),
- the estimated Poisson distribution for adjusted experiment and background (Figure 2.4, bottom left; Section 1.3.7, Figure 1.4c),
- the count distribution for experiment and background (Figure 2.4, top middle; Section 1.3.5, Figure 1.4b),

- the p-value distribution over all windows (Figure 2.4, bottom middle; Section 1.3.12, Figure 1.6),
- the mapping quality (Figure 2.4, top right; Section 1.3.3, Figure 1.3),
- the distribution of significant windows per chromosome (Figure 2.4, bottom right; Section 1.3.11, Figure 1.5).

This serves for assessing the quality of each of the replicates and for deciding, how to adjust the combination of replicates for the final results.

2.4.4 Correlation Information, Recalculation Parameters, and Restarting Computation

The 'Summary' tab (Figure 2.5)

- shows the Pearson Correlation of the replicates (Figure 2.5, left top; Section 1.3.13, Figure 1.7),
- allows for enabling or disabling the correlation based correction (Figure 2.5, left bottom, checkbox; Section 1.3.13, Figure 1.7),
- allows for setting a weight affecting the combination of the single replicates for creating the combined peaks (Figure 2.5, right top, middle column; Section 1.3.14, Figure 1.8),
- allows for enabling (ON) or disabling (OFF) a replicate (Figure 2.5, right top, right column; Section 1.3.14, Figure 1.8),
- allows for setting two parameters and restarting the computation (Figure 2.5, right bottom row; Section 1.3.14, Figure 1.8).

The user can enable correlation correction and select a weight for each replicate, which is used while combing the p-values. Additionally, it is possible to exclude one or more replicates from the recalculation and change to the q-value correction method. Moreover, quality counting can be enabled or disabled.

The defaults used during the initial run (Section 1.3.14) are: correlation correction is enabled, all replicates are enabled, the p-value correction method is set to "Holm-Bonferroni", and the weights are set to the ω values of each replicate (Equation 1.3, Section 1.3.5). Further, the peak quality computation is computed by default.

Finally, the recalculation is started with the new settings. Therefore, it is necessary to send the job to the server again.

2.4.5 Peak Information

The 'Peak information' tab shows two diagrams:

- the final p-value histogram showing the combined p-values for all windows (Figure 2.6, left; Section 1.3.15, Figure 1.10; Section 1.3.14, Figure 1.9)

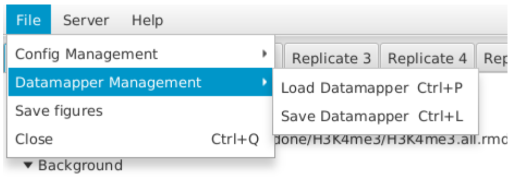

Figure 2.7: The File Menu showing the sub menu for storing and loading the data mapper.

- the percentage of agreement of each replicate with the multiple-replicate result (Figure 2.6, right; Section 1.3.18, Figure 1.11a; Section 1.3.14, Figure 1.11b)

2.4.6 Quality Information

The 'Quality information' tab shows the distribution of the peak quality for all replicates (Section 1.3.21, Figure 1.12).

2.4.7 Additional Functionality

2.4.7.1 Loading and saving the data mapper

Sierra Platinum automatically exports a data mapper file for each run, which contains all information that are presented in the GUI. With this mapper, it is possible to archive all the additional data created by Sierra Platinum for the peak-calling and to analyze this data even without a server connection. The data mapper can be exported by using the menu entry 'File → Datamapper Management → Save Datamapper' and imported by using 'File → Datamapper Management → Load Datamapper' (Figure 2.7).

The client configuration can be exported and imported like the Datamapper using the 'Config Management' menu entry.

2.4.7.2 Export graphics

All figures created by Sierra Platinum can be exported as a bundle or as a single figure for later utilization as *.png* files. The user can export all figures by using the Menu Entry 'File → Save figures' (Figure 2.7). Single figures can be exported by clicking them with the right mouse button.

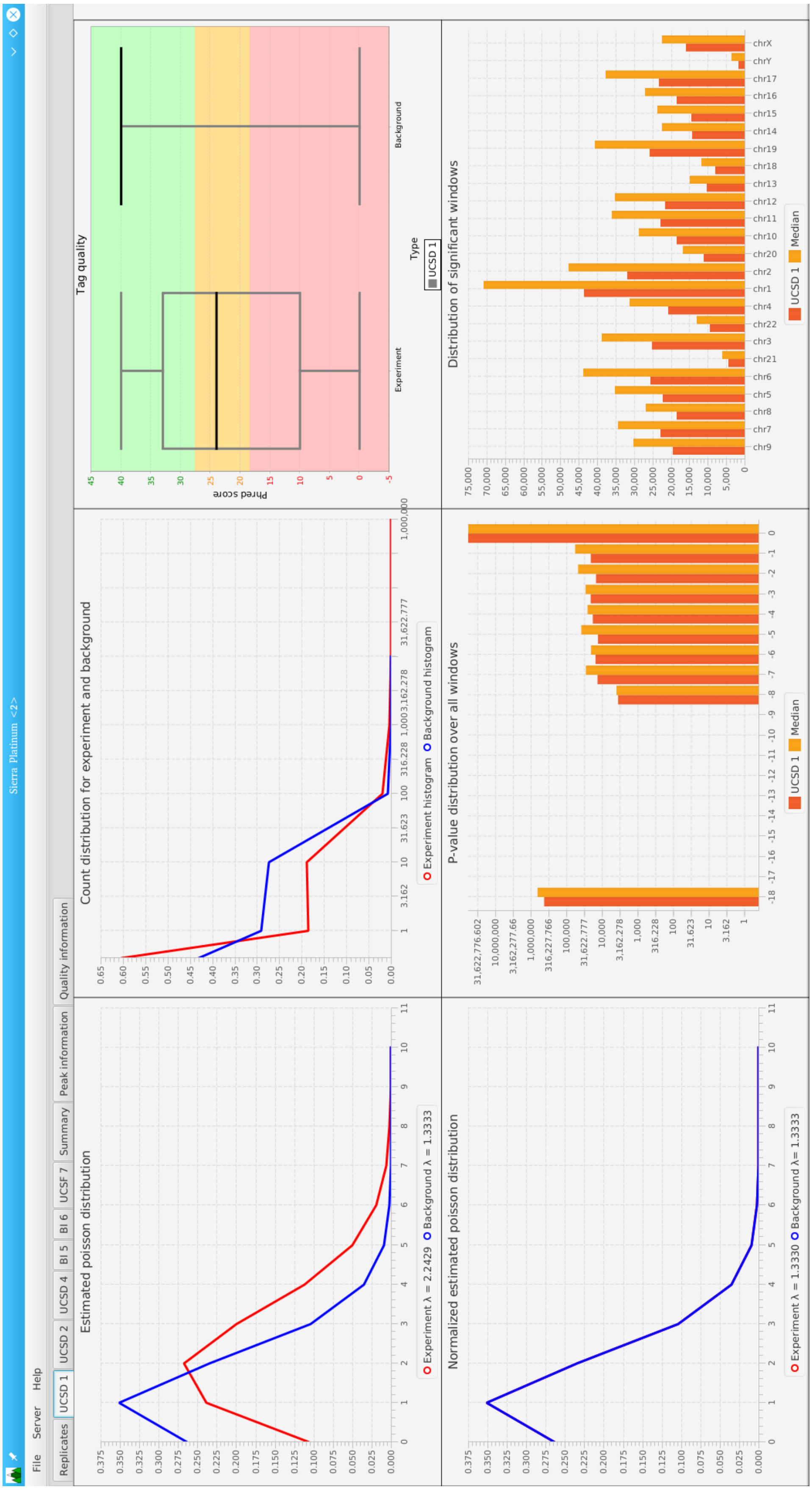

Figure 2.4: A replicate tab in the GUI of Sierra Platinum. Top left: the estimated Poisson distribution for experiment and background. Top middle: the count distribution for experiment and background. Top right: the mapping quality. Bottom left: the estimated Poisson distribution for adjusted experiment and background. Bottom middle: the p-value distribution over all windows. Bottom right: the distribution of significant windows per chromosome.

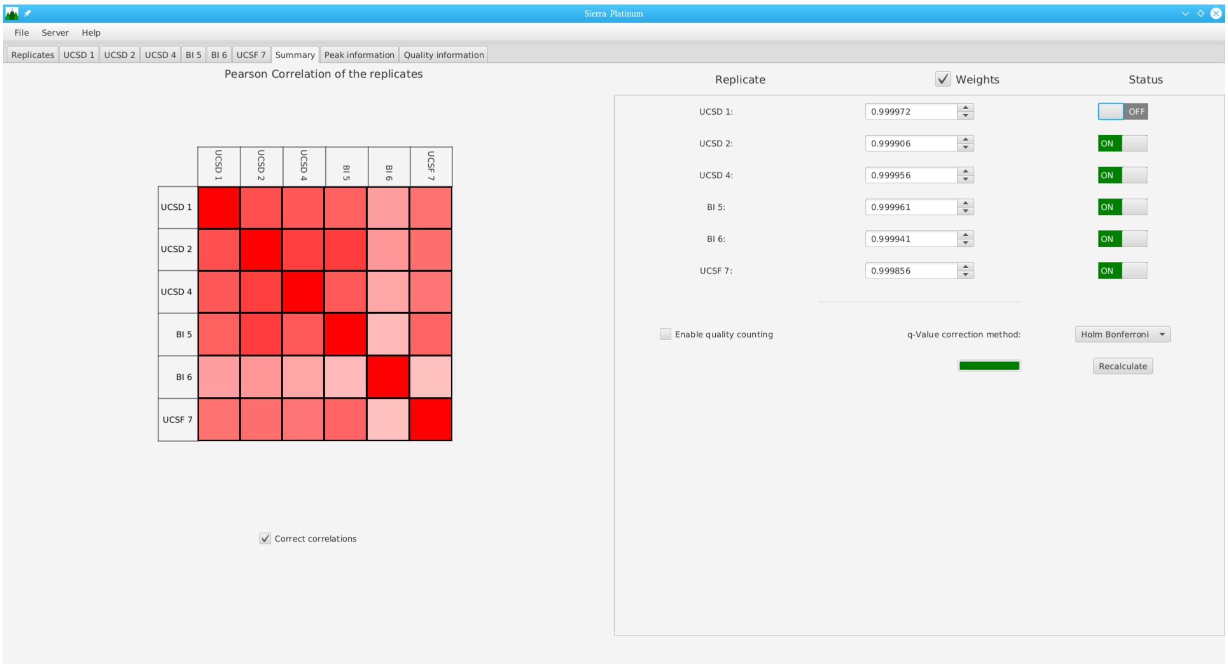

Figure 2.5: The ‘Summary’ tab in the GUI of Sierra Platinum. Left top: the Pearson Correlation of the replicates. Left bottom: checkbox allowing for enabling or disabling the correlation based correction. Right top, middle column: weights affecting the combination of the single replicates for creating the combined peaks. Right top, right column: enabling (ON) or disabling (OFF) a replicate. Right bottom row: two parameters and restarting the computation.

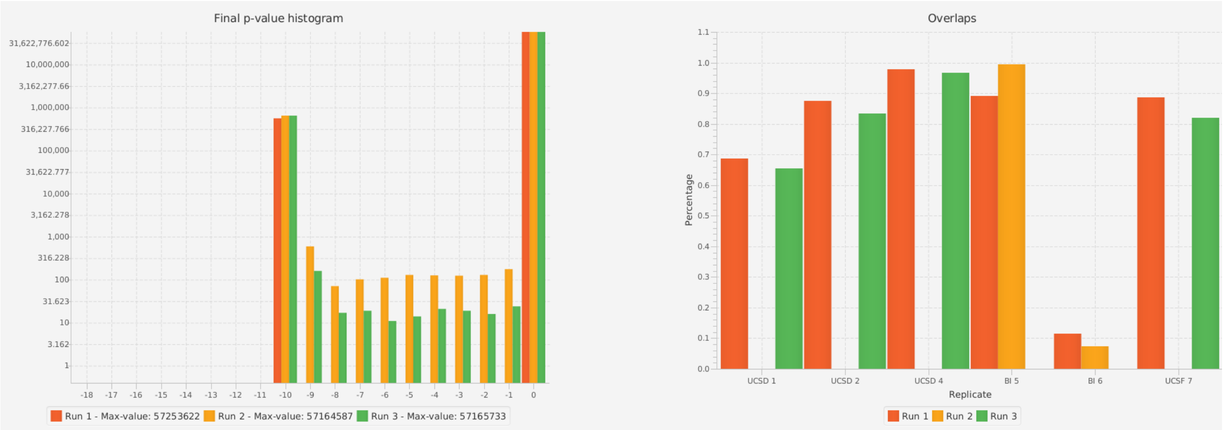

Figure 2.6: The ‘Peak information’ tab in the GUI of Sierra Platinum. Left: the final p-value histogram showing the combined p-values for all windows. Right: the percentage of agreement of each replicate with the multiple-replicate result. Three runs with different setting for weights and different replicate combinations are shown.

Chapter 3

Optimization

3.1 Goal

Writing this multiple-replicate peak-caller is a technological challenge due to the amount of input data. A straight-forward approach would be slow and memory intense. Therefore, the goal was to optimize the solution presented in the previous chapters: the computation time should be minimal (time efficiency) and the amount of memory used should be low (space efficiency).

3.2 Context and Data Sets

Throughout this description, we provide timings that are obtained using the hardware configuration of the server and the software shown in Table 3.1. We set the Java garbage collector to the new, still experimental G1 garbage collector. To account for variations, each test case was performed three times and the average time over these three runs is reported.

For assessing the performance, we used data from the NIH Roadmap Epigenomics Project [24]. More specifically, we used ChIP-seq data for trimethylations at lysine at position 4 at histone H3 (H3K4me3) in the embryonic stem cell line H1 and the corresponding ChIP-input of 6 replicates. All data sets were downloaded, extracted, clipped and mapped. We used `segemehl` [16] to map the reads to the human genome version hg19 with 80% accuracy. SAMtools [22] were used to convert the SAM files produced by `segemehl` into bam files, to sort them, to remove PCR replicates, and to create bam index files (bai files).

Table 3.1: Hardware and Software Specification

	Specification
Memory	32 GB
CPU	2 x Intel(R) Xeon(R) CPU E5-2630 v3 @ 2.40GHz (total: 16 cores, 32 threads)
Operating System	Kubuntu, Ubuntu 14.04.3 LTS, 3.13.0-63-generic Ubuntu SMP, 64 bit.
Programming Language	Java v8 (build 1.8.0_60-b27)

Two parameter settings were used. The first uses a window offset of 100nt, a window size of 400nt, and a cutoff value of $p = 10^{-5}$. The second uses a window offset of 50nt, a window size of 200nt, and a cutoff value of $p = 10^{-5}$.

Each replicated experiment consists of the experiment itself and the associated background, the experiment is compared to. Overall, r replicates and $n = 2 \cdot r$ data sets (r experiments and r backgrounds) are considered.

3.3 Approach

To achieve an acceptable time efficiency, a standard concept of Java was used: threads. Whenever suitable, the data to be computed is distributed onto several threads allowing to compute information in parallel. The number of available threads depends on the CPU(s) of the computer. Typically, Intel-based CPUs provide two threads per core and several cores per CPU. In the test case used here, at most 32 threads could be used.

The parallelization concept used here is data parallelism. The complete algorithm of Sierra Platinum described in Chapter 1 is handled sequentially. Whenever a part of the algorithm is applied to a large amount of data such that there is no dependency between individual computations, the data is split into blocks and each block of data is assigned to one thread. A typical block of data could be, e.g., one replicate or one data set (background or experiment). Taking all windows, a set of n windows can also form a block.

An overview of the complete peak-calling process is given in Section 3.4. Details are described in the respective sub-sections of Section 3.5 including which steps of Sierra Platinum needed to be and could be parallelized and which parallelization approaches were evaluated.

Table 3.2: The steps performed by Sierra Platinum (Chapter 1): section, the name of the step, whether it is executed in parallel or sequentially, the default number of threads used, and the time used in mm:ss for the two parameter settings using the optimal variants of the algorithms. Steps with empty cells were merged into other steps or are purely interactive.

Section	execution	default number of threads	100nt, 400nt (mm:ss)	50nt, 200nt (mm:ss)
	Phase I			
3.5.1 Constructing Windows	parallel	6	08:53	15:32
3.5.2 Joining Windows	sequential	1		
3.5.3 Computing Mapped Read Quality	merged			
3.5.4 Computing the Poisson Distribution	sequential	1	00:13	00:43
3.5.5 Computing the Tag Count Distribution	parallel	32	00:49	00:26
3.5.6 Scaling Experiments	parallel	32	00:03	00:08
3.5.7 Computing the Normalized Poisson Distributions	sequential	1	00:13	00:31
3.5.8 Computing Neighborhoods	merged			
3.5.9 Computing Single Replicate P-Values	parallel	32	02:06	10:09
3.5.10 Converting P- to Q-Values	parallel	32	00:42	03:46
3.5.11 Determining the Significant Windows	sequential	1	00:14	00:30
3.5.12 P-Value Distribution	parallel - sequential	32 - 1	00:48	01:42
	Phase II			
3.5.13 Establishing Pearson's Correlation between Replicates	parallel	32	00:22	00:51
3.5.14 Filtering and Weighting Replicates	interac-tion			
3.5.15 Computing the Combined P-Value	parallel	32	00:02	00:15
	Phase III			
3.5.16 Converting the Combined P-Value into Q-Value	sequential	1	00:11	00:20
3.5.17 Final P-Value Distribution	sequential	1	00:05	00:10
3.5.18 Computing the Agreement between the Multi-Replicate and Single Replicate Results	sequential	1	00:01	00:02
3.5.19 Computing Narrow Peaks	sequential	1	00:01	00:01
3.5.20 Computing Broad Peaks	sequential	1	00:01	00:01
3.5.21 Computing Peak Quality	parallel	6	05:19	13:36
3.5.22 Storing the Results	sequential	1	00:01	00:01
Total			20:03	48:46

Algorithm 1 Construct Windows

Determine windows overlapping at least one tag in one data set
Merge these windows (some strategies, see below)
Flattening: determine final, ordered window list

3.4 Optimization of the Multi-Replicate Peak-Calling Process: Overview

Following the structure of the article describing the methods and functionality of Sierra Platinum (Chapter 1), we describe the computation strategies—data structures and algorithms—implemented and compared that allowed us to optimize the run time and the space requirements of the tool. Table 3.2 shows an overview of the optimizations performed. For each step, a reference to its section, the name of the step, whether it is executed in parallel or sequentially, the default number of threads used, and the time used in mm:ss for the two parameter settings using the optimal variants of the algorithms. The section and the name are the same as in the description of the functionality (Chapter 1). Some steps were merged into other steps or are purely interactive steps.

For the complete peak-calling process, the optimized version of Sierra Platinum uses ≈ 20 minutes and ≈ 20 GB of heap memory (window offset: 100nt, window size: 400nt) and ≈ 50 minutes and ≈ 26 GB of heap memory (window offset: 50nt, window size: 200nt) respectively. Please notice, that due to the use of Java, only an estimation of the memory consumption is possible.

3.5 Optimization of the Multi-Replicate Peak-Calling Process: Detailed Description

3.5.1 Constructing Windows

Constructing the windows is the first step of Sierra Platinum. Windows having a length of l nt are searched for. The offset between two subsequent windows is o nt. A window is constructed whenever the l nt under consideration overlap with tags from at least one experiment or background of one replicate. The number of overlaps is counted and stored with each window.

The general procedure for constructing windows is outlined in Algorithm 1. To accelerate this step, different parallelization strategies were developed and tested:

Tag Count Parallel: count tags in parallel (Section 3.5.1.1)

Chromosome Parallel: create one thread per chromosome (Section 3.5.1.2)

Algorithm 2 Chromosome Parallel

```
for each chromosome (parallel) do
  for each window do
    for all data sets do
      count tags overlapping this window
    end for
    if at least one overlapping tag is found then
      add window to window list
    end if
  end for
end for
```

Chunk Parallel: count chunks in parallel (Section 3.5.1.3)

One important constraint during optimization was to keep the space consumption of all data structures—whether permanent or temporary—at a minimum. Thus, we minimized the computation time while always considering to keep the memory consumption low.

3.5.1.1 Tag Count Parallel

The first idea was, to count tags in parallel. First, all windows are constructed sequentially. Then, for each window, the number of tags overlapping this window is counted for each data set. Parallelization was achieved by assigning a certain number of windows to each available thread. During flattening, only those windows are copied to the final list of windows that contain at least one tag in one data set.

This method is simple, but a lot of windows does not overlap any tags. These windows are empty and need not be generated and kept until flattening in the first place. They need to be removed by the garbage collector which takes additional time. The experiments show that the number of threads used for computation should be maximal.

The method uses more than 9 and a half hours (offset: 100nt, size: 400nt) and more than 37 hours (offset: 50nt, size: 200nt) of wall clock time, respectively (Table 3.3). The additional time used for flattening is ≈ 4 seconds (offset: 100nt, size: 400nt) and ≈ 12 seconds (offset: 50nt, size 200nt), respectively.

3.5.1.2 Chromosome Parallel

The second idea is, to count the tags for each chromosome in parallel. For each chromosome, one thread is established and within this thread Algorithm 2 is performed.

As the chromosomes have very different lengths, this leads to an unbalanced use of threads, which shows in the time used for computing the windows. Moreover, it can be observed that the number of threads used reduces over time until at the end only one thread is busy.

Overall, this methods needs more than 15 hours of wall clock time; 5 and a half hours more than 'Tag Count Parallel' for a window offset of 100nt and a window size of 400nt (Table 3.3). However, for a window

Table 3.3: Constructing all windows for 6 replicates: time for different strategies

Strategy	100nt, 400nt (hh:mm:ss)	50nt, 200nt (hh:mm:ss)
Tag Count Parallel	09:38:22	37:02:14
Chromosome Parallel	15:13:13	29:56:02
Chunk – Window – Dataset	08:53:58	18:48:29
Chunk – Dataset – Window	09:28:09	19:49:34
Dataset – Chunk – Window	10:12:28	out-of-memory
Chunk Parallel Coherent	00:09:32	00:15:32

Algorithm 3 Chunk – Window – Dataset

```
for for all chunks (parallel) do
  for for all windows of this chunk do
    for for all data sets do
      count tags overlapping this window
    end for
  end for
end for
```

Algorithm 4 Chunk – Dataset – Window

```
for for all chunks (parallel) do
  for for all data sets do
    for for all windows of this chunk do
      count tags overlapping this window
    end for
  end for
end for
```

offset of 50nt and a window size of 400nt, it needs 7 hours less than 'Tag Count Parallel': ≈ 30 hours (Table 3.3). The additional time used for flattening is ≈ 14 seconds (offset: 100nt, size: 400nt) and ≈ 41 seconds (offset: 50nt, size: 200nt), respectively.

3.5.1.3 Chunk Parallel

To achieve a more equalized distribution of the workload for each thread, while adding only those windows that contribute to the final result, chunks of equal size are created. Each chunk contains c windows, where c is a predefined chunk size. Each chunk is assigned to a thread such that chunks are computed in parallel. For each window, the tag count for each data set is computed. Thus, three loops are used for the computation: one for the chunks, one for the windows of the chunks, and one for the data sets. Rearranging these loops yields the three variations of this strategy described by Algorithms 3–5 (Section 3.5.1.3.1–Section 3.5.1.3.3).

After computing all windows, the final, ordered window list is determined. This step is necessary, because nested data structures are used by the individual variations of the strategy.

3.5.1.3.1 Chunk – Window – Dataset In this variation (Algorithm 3), all chunks are handled in parallel, the windows of each chunk are constructed sequentially, and the overlaps of each window with tags from each data set are computed sequentially. Thus, it is always clear, if a window can be added to the list, or not.

For this strategy, the following data structure is used. Each window is added to its chunk sequentially. All chunks of a chromosome are stored in a list, according to their number. This yields a list of chunks, with each chunk containing a list of windows. Finally, a map from each chromosome to its ordered list of chunks is used. This nested structure is flattened to obtain the final ordered window list.

For a window offset of 100nt and a window size of 400nt, the time needed by this approach (≈ 9 hours, Table 3.3) is half an hour less than for 'Tag Count Parallel', while the order of magnitude is the same. However, for a window offset of 50 nt and a window size of 200nt, it is considerable faster (≈ 19 hours, Table 3.3) than both 'Tag Count Parallel' and 'Chromosome Parallel'. The time used for flattening is ≈ 20 seconds (offset: 100nt, size: 400nt) and ≈ 53 seconds (offset: 50nt, size: 200nt), respectively.

3.5.1.3.2 Chunk – Dataset – Window In this variation (Algorithm 4), all chunks are again handled in parallel. However, only the tags for the current data set are counted for each window.

Therefore, the data structure was changed. The chromosome is mapped to a map from window start position to window. Thereby, windows having tag counts for previously considered data sets are retrieved from the window map and the new tag counts for the current data set are added.

The run-time needed is similar to 'Chunk – Window – Dataset' (Table 3.3). Flattening takes ≈ 20 seconds (offset: 100nt, size: 400nt) and ≈ $2:10$ minutes (offset: 50nt, size 200nt), respectively.

3.5.1.3.3 Dataset – Chunk – Window The data structure used for this variation (Algorithm 5), is the same as for the previous one. For a window offset of 100nt and a window size of 400nt, the computing times were similar to the previous two strategies (Table 3.3). The time used is 45 minutes longer while the

Algorithm 5 Dataset – Chunk – Window

```
for for all data sets do
  for for all chunks (parallel) do
    for for all windows of this chunk do
      count tags overlapping this window
    end for
  end for
end for
```

time needed for flattening stayed the same. However, 26GB of memory are not sufficient for computing the windows using a window offset of 50nt and a window size of 200nt.

3.5.1.3.4 Summary All three strategies described produce runtimes in the same order of magnitude. The overall load is the same. It can be observed that the load is *not* IO bound as all threads use the complete available computing time and use a maximum of available threads. Moreover, a large number of disc locks can be observed. However, the strategy Dataset – Chunk – Window needs more memory and could not be used to compute the example with a window offset of 50nt and a window size of 200nt. Overall, the strategy Chunk – Window – Dataset is fastest.

3.5.1.4 Chunk Parallel Coherent

As we found that IO is not yet the limit, an additional idea was exploited. The time consumption is large, as long as the tags overlapping individual windows are fetched from the file. Therefore, instead of fetching the tags for each window, the tags for the complete chunk are fetched, and then the tags are counted for each window belonging to this chunk. This strategy improved performance, i.e., reduced computation time, by an order of magnitude from more than 9 hours to less than 10 minutes (offset: 100nt, size: 400nt) and from more than 19 hours to less than 16 minutes (offset: 50nt, size: 200nt), respectively (Table 3.3). Further, the load shows that now IO is the limiting factor and no longer CPU power.

As all tags are already processed, this allows to compute the mapped read quality (Section 3.5.3) and the tag count of the data set (Section 3.5.5) at the same time as the windows instead of in separate steps. As the fetching of data is comparatively slow—in fact it is the bottleneck of the computation—this additionally reduces the time needed for these steps by a factor of three.

Testing sequential versus parallel execution showed that a certain number of threads is beneficial for reducing the computation time (see Table 3.4). However, after saturation, adding threads will decrease performance again. For our example and computational environment, an optimal number of 6 threads was determined. Please notice, that the optimal number of threads depends on the system architecture and might vary.

Table 3.4: The time needed for constructing all windows for 6 replicates using the strategy Chunk Parallel Coherent with different thread pool sizes

Number of Threads	100nt, 400nt (mm:ss)	50nt, 200nt (mm:ss)
1	17:48	22:53
2	14:00	14:47
4	11:31	12:10
**6**	**08:53**	**10:45**
8	10:23	13:05
16	10:10	14:03
32	11:06	15:07

3.5.2 Joining Windows

This part is described together with the window construction in Section 3.5.1.

3.5.3 Computing Mapped Read Quality

To obtain the mapped read quality, we calculate for each data set the median, the lower and the upper quartiles, and the minimum and the maximum value of the Phred score distribution.

This step can be joined with the window construction step, if strategy 'Chunk Parallel Coherent' is used (Section 3.5.1.4). In this case, it will not use additional time.

Otherwise, it takes $\approx$ 11 minutes (offset: 100nt, size: 400nt) and $\approx$ 13 minutes (offset: 50nt, size: 200nt), respectively. Parallelization using threads does not decrease the time needed, as this step is essentially IO limited.

3.5.4 Computing the Poisson Distribution

Computing the distribution of the data and its noise model is already fast—$\leq$ 15 seconds (offset: 100nt, size: 400nt), $\leq$ 45 seconds (offset: 50nt, 200nt)—and therefore was not optimized.

3.5.5 Computing the Tag Count Distribution

Computing the tag count distribution of the real data, i.e., the relative frequency for each observed tag count is performed in parallel for all replicates by assigning each replicate to a thread. At the same time, the least square distances to the Poisson distribution (for each data set), the bins (for each data set), and the final weights (for each replicate) are computed. Overall, the computation time is $\approx$ 50 seconds (offset: 100nt, size: 400nt), $\approx$ 26 seconds (offset: 50nt, size: 200nt).

3.5.6 Scaling Experiments

In Sierra Platinum, each experiment is scaled such that the library sizes of experiment and background of a replicate measured as total number of mapped tags are equal. Scaling is done for all windows in parallel. Therefore, each window is assigned to a thread and all experiments of all replicates are scaled. This step is very fast taking ≤ 5 seconds (offset: 100nt, size: 400nt) and ≤ 8 seconds (offset: 50nt, size: 200nt), respectively. Thus, no further optimization is necessary.

3.5.7 Computing the Normalized Poisson Distributions

Computing the distribution of the scaled data and its noise model is already very fast taking ≤ 15 seconds (offset: 100nt, size: 400nt) and ≤ 35 seconds (offset: 50nt, size: 200nt), respectively, and therefore was not parallelized.

3.5.8 Computing Neighborhoods

The neighborhoods of 1k, 5k, and 10k for each window are required during the p-value calculations for the single replicates to account for local sequence composition biases (Section 3.5.9).

Several options for storing and computing neighborhoods were explored:

1. Compute and Store
   (a) Store with window (computed and stored during flattening)
   (b) Store separately (computed and stored before p-value computation)
2. Compute and Use during p-value computation

Compute and Store with Window The first solution computes the neighborhood while constructing the window list during the flattening phase. The indices of the first and of the last window of the respective neighborhood are stored with each window, which requires six additional values per window. As flattening is performed sequentially, the neighborhoods are also created sequentially.

Compute and Store Separately Storing the neighborhood with each window is not necessary as it is only used for one step ('Computing Single Replicate P-Values', Section 3.5.9). To minimize space consumption, the two steps were separated and the neighborhoods are constructed just before they are used. Thus, the second solution computes all neighborhoods for all windows and stores them in a separate class. The storage of the neighborhoods is released immediately after the single replicate p-value computation (Section 3.5.9).

This step is time consuming and therefore was parallelized. As neighborhoods of a window belong to

Table 3.5: Computing window neighborhoods and p-values for 6 replicates and 3 different neighborhood sizes: time for different strategies.

<table>
<tr><th>Strategy</th><th>Neighborhood (mm:ss)</th><th>p-value (mm:ss)</th></tr>
<tr><td>Compute and Store with Window</td><td>< 00:30</td><td>02:57</td></tr>
<tr><td>Compute and Store Separately</td><td>00:55</td><td>02:57</td></tr>
<tr><td>Compute and Use During p-Value Computation</td><td colspan="2">02:06</td></tr>
</table>

the same chromosome as the window itself and coherence should be exploited, a parallelization over chromosomes was chosen.

Compute and Use During p-Value Computation The final solution does not store neighborhoods any more, but directly computes the corresponding λ that is then directly used for computing the single replicate p-values (Section 3.5.9). Therefore, each of the neighborhoods is initialized by its range, the window list, and the replicate list. Internally, the indices into the window list of the first and of the last neighbor are stored. As the windows are processed sequentially to obtain the corresponding λ-values, the indices are updated for each window. At the same time, the tag count of the window's neighborhood is updated. The parallelization is again over all chromosomes.

Summary The time consumption of the different solutions (including p-value computation) is given in Table 3.5. It shows that the most space efficient solution—computing and using the neighborhood directly during p-value computation—is also the fastest solution.

3.5.9 Computing Single Replicate P-Values

The final λ value for each window is computed as the maximum of the global λ value and the λ-values of each neighborhood. For each window and for each replicate, a p-value is computed from the final λ value and the tag counts.

When computing the neighborhoods explicitly for all windows, the single replicate p-values are computed in parallel over all windows assigning an equal number of windows to each thread:

$$n = \frac{|windows|}{|cores|} + 1 \quad (3.1)$$

except for the last thread, which handles the remaining windows. This heavily reduces the time needed. It scales with the number of threads available. The

Table 3.6: Time needed for transforming p- to q-values using different strategies

Strategy	100nt, 400nt (mm:ss)	50nt, 200nt (mm:ss)
Holm-Bonferroni	01:45	03:35
Storey-Simple	07:09	25:26
Storey-BootStrap Spline	04:04	03:52

Table 3.7: Computing Pearson's correlation for 6 replicates: time for different strategies.

Strategy	100nt, 400nt (mm:ss)	50nt, 200nt (mm:ss)
Sequential	3:38	8:00
Parallel row	0:53	1:50
Parallel all	0:22	0:45

time used for the given configuration is $\approx$ 3 minutes (Table 3.5).

Using the space efficient version, the single-replicate p-values for each window are directly computed from the window and its neighborhood in one step. All chromosomes are computed in parallel by assigning each chromosome to a thread that then computes the p-values (and the neighborhoods) for all windows on this chromosome. The combined approach uses only $\approx$ 2 minutes (offset: 100nt, size: 400nt) (Table 3.5) and $\approx$ 10 minutes (offset: 50nt, size: 200nt), respectively, and the space consumption is minimal.

3.5.10 Converting P- to Q-Values

The p-value correction methods are run in parallel for each replicate. Each method was split into two parts: sorting the p-values and converting p- to q-values. For sorting the p-values, 32 threads are used, while for converting only 6 from the 32 threads available are used (one per replicate). The overall time needed by the three different methods implemented are shown in Table 3.6. For a window offset of 100nt and a window size of 400nt, the Holm-Bonferroni correction can be computed relatively fast using 1:45 minutes, both Storey-Simple with 7:09 minutes and Storey-BootStrap Spline with 4:04 minutes take longer. For a window offset of 50nt and a window size of 200nt, the Holm-Bonferroni correction can be computed relatively fast using 3:46 minutes, Storey-BootStrap Spline with 3:52 minutes takes approximately the same time, and Storey-Simple takes much longer using 25:26 minutes. Moreover, the Storey-BootStrap Spline correction is dependent on random numbers and thus does not produce consistent results over different runs.

3.5.11 Determining the Significant Windows

Sierra Platinum provides for each replicate the distribution of the significant windows: for each chromosome c and for each replicate i, we count the number of significant windows s_i^c. Additionally, we calculate the median distribution of the significant windows for each chromosome.

This step is already very fast using $\leq$ 15 seconds (offset: 100nt, size: 400nt) and $\leq$ 30 seconds (offset: 50nt, size: 200nt), respectively, and therefore was not parallelized.

3.5.12 P-Value Distribution

Computing the distribution of the p-values uses $\approx$ 50 seconds (offset: 100nt, size: 400nt) and $\approx 1 : 45$ minutes (offset: 50nt, size: 200nt), respectively. The first part of the computation is parallelized assigning each replicate to a thread, while the second part is sequential.

3.5.13 Establishing Pearson's Correlation between Replicates

To compute the Pearson's correlation between the replicates, the mean and the standard deviation of the probits over all windows for each replicate are computed, as well as the correlation between the replicates themselves. Three possible strategies were evaluated:

1. Sequential computation: one thread is used for all computations
2. Parallel: compute sequentially each of the following steps in parallel
   (a) Computing the mean values of the replicates: one thread per replicate
   (b) Computing the standard deviations of the replicates: one thread per replicate
   (c) Computing the Pearson correlation of each pair of replicates. This results in a correlation matrix. The main diagonal of this correlation matrix is always one and not computed. Further, upper and lower triangle of the correlation matrix are symmetric. Therefore, only the upper triangle is computed.
      i. Row: compute one row after each other, one thread per column
      ii. All: use one thread per correlation computed

The results for computing the correlation between six replicates are shown in Table 3.7. Computing all correlations in parallel is fastest followed by computing each row in parallel. Computing all steps sequentially is very slow, as expected.

3.5.14 Filtering and Weighting Replicates

This step is purely interaction. Only its results are used as parameters for the subsequent steps.

3.5.15 Computing the Combined P-Value

The combined p-value is computed in parallel over all windows assigning an approximately equal number of windows to each thread except the last:

$$n = \frac{|windows|}{|cores|} + 1 \tag{3.2}$$

This heavily reduces the time needed and scales with the number of threads available. The time needed is $\leq$ 5 seconds (offset: 100nt, size: 400nt) and $\leq$ 15 seconds (offset: 50nt, size: 200nt).

3.5.16 Converting the Combined P-Value into Q-Value

The conversion of the combined, multiple-replicate p-value into a q-value for each window was not parallelized and takes $\leq$ 15 seconds (offset: 100nt, size: 400nt) and $\leq$ 20 seconds (offset: 50nt, size: 200nt), respectively.

3.5.17 Final P-Value Distribution

The computation the distribution of the final p-values uses $\leq$ 5 seconds (offset: 100nt, size: 400nt) and $\leq$ 10 seconds (offset: 50nt, size: 200nt), respectively, and was not parallelized.

3.5.18 Computing the Agreement between the Multi-Replicate and Single Replicate Results

For computing the agreement between the multiple-replicate result and the single replicate results, i.e., for calculating the fractions of the significant windows according to the combined p-value that are also significant in the different replicates, $\leq$ 5 seconds (both offset: 100nt, size: 400nt and offset: 50nt, size: 200nt) are needed. Therefore, this step was not optimized.

3.5.19 Computing Narrow Peaks

Computing narrow peaks—significant windows overlapping in their genomic position—uses $\leq$ 5 seconds (both offset: 100nt, size: 400nt and offset: 50nt, size: 200nt). Therefore, this step does not need to be optimized.

3.5.20 Computing Broad Peaks

Computing broad peaks also uses $\leq$ 5 seconds (both offset: 100nt, size: 400nt and offset: 50nt, size: 200nt). Therefore, this step does not need to be optimized.

3.5.21 Computing Peak Quality

The computation of the median read quality for each peak is parallelized similarly to the window construction step (Section 3.5.1). The strategies implemented and evaluated are:

Peak Parallel: Compute the quality for all peaks in parallel (Section 3.5.21.1)

Peak Coherent Parallel: Compute the quality for a subset of peaks in parallel (Section 3.5.21.2)

- Plain: Get the reads overlapping the interval containing all peaks in the subset, from the start of the first peak until the end of the last peak in the set, for computing the peak quality (Section 3.5.21.2.1)
- Space: Get the reads overlapping the interval containing all peaks in the subset, from the start of the first peak until the end of the last peak in the set, for computing the peak quality using less space than Plain (Section 3.5.21.2.2)
- Broad-Narrow: Get the reads overlapping the interval for all broad peaks in the subset and compute the quality of broad and narrow peaks in parallel (Section 3.5.21.2.3)
- Smart: Get only reads overlapping the peaks of the subsets for computing the quality (Section 3.5.21.2.4)

3.5.21.1 Peak Parallel

For each replicate, the peak quality is calculated separately for narrow and broad peaks. Each of these calculations is done in parallel. Each thread handles a number of peaks from the peak list in parallel. This strategy is similar to the 'Chunk Parallel' strategy during window construction. The time needed for computing all peaks' qualities using this strategy is $\approx$ 16 minutes (Table 3.8) for a window offset of 100nt and a window size of 400nt. For a window offset of 50nt and a window size of 200nt, the available memory was not sufficient for this strategy.

3.5.21.2 Peak Coherent Parallel

3.5.21.2.1 Plain As the coherent strategy proved to be useful for the window construction step, it was also applied here. Instead of fetching the interval of reads overlapping with a single peak, all reads in the interval starting at the first peak until the end of the last peak of a list of peaks are fetched. Using the same amount of threads, the computation uses $\approx$ 60 minutes (offset: 100nt, size: 400nt). Increasing the number of threads to 8 leads to an increase of computation time used to $\approx$ 80 minutes. Increasing the number of threads to 12 leads to either an out-of-memory error (20 GB memory) or an increase of computation time used to > 8 hours (30 GB of memory). Increasing the number of threads to 32 leads to an out-of-memory error.

For the second example (offset: 50nt, size: 200nt), the time increases to $\approx 2:48$ h using 6 threads. As already for the first example memory problems occur if the number of threads are increased, an increase of threads was not tested for this example.

Table 3.8: Computing peak quality for 6 replicates: time for different strategies (6 threads)

Strategy	100nt, 400nt (hh:mm:ss)	50nt, 200nt (hh:mm:ss)
Peak Parallel	16:02	out-of-memory
Peak Coherent Parallel – Plain	59:59	2:48:06
Peak Coherent Parallel – Space	23:28	37:31
Peak Coherent Parallel – Space – Smart	17:49	31:18
Peak Coherent Parallel – Broad-Narrow	07:26	13:54
Peak Coherent Parallel – Broad-Narrow – Smart	05:19	13:36

3.5.21.2.2 Space The original approach uses two queues to store all reads. This allows a simple logic for computing the overlap between the reads and the peaks. Using only one queue requires a more complicated logic for this computation but is much more space efficient. First of all, the pointers in the first queue referencing reads are not needed. More important is, however, that less reads are hold concurrently in memory. This allows for a large reduction concerning the space requirements.

3.5.21.2.3 Broad-Narrow As all narrow peaks are parts of broad peaks, all reads needed for establishing their quality are already considered while computing the quality of the broad peaks. Therefore, both can be computed in one step. This saves ≈ 50% of the computation time at the expense of using more memory due to needing to store a copy of these reads. This can lead to a large memory consumption in case of very broad peaks. Therefore, not the objects obtained from the HTSJDK library [1] holding the complete information are stored. Only the information needed is copied to a new object that is then stored until it is not needed any more. This reduces the maximal space consumption of Sierra Platinum while increasing the amount of garbage collection needed.

3.5.21.2.4 Smart In the plain coherent strategy, the reads overlapping empty parts between the peaks and not necessarily any peak are also fetched. Using the property of the HTSJDK library [1] to retrieve an array of intervals can be used to reduce the amount of reads considered. This strategy reduced computation time by approximately 30% to ≈ 5:30 minutes (offset: 100nt, size: 400nt) in combination with 'broad-narrow' (Table 3.8).

For the smaller window offset and window size, the smart strategy is similar to the non-smart one; ≈ 13:30 minutes (offset: 50nt, size: 200nt). However, all time measurements for the second example have a large variance of 11:45 – 15:11 minutes.

3.5.21.3 Results

Overall, the combination of the 'Broad-Narrow' and the 'Smart' variation of the 'Peak Coherent Parallel' strategy gives the least time needed. However, the space consumption is higher than for 'Peak Coherent Parallel – Space – Smart'. Here, a clear trade-off between time and space needed exists and has to be decided upon the local configuration of the server, especially, the memory available for computation.

3.5.22 Storing the Results

During each run, the following information is stored: the parameters that were used for the current step of the computation, the data mapper—an internal structure of Sierra Platinum containing all information needed for the visualizing the various quality measures computed—, and the resulting peaks as *bed* and as *csv* files with the p-value as the score field. Exporting all this information takes ≈ $1 - 2$ seconds (both offset: 100nt, size: 400nt and offset: 50nt, size: 200nt).

If needed, the complete state comprising the data mapper and the window list can be stored. However, window list creation is fast compared to storing and loading the state, which currently takes ≈ 7 minutes.

Sierra Platinum uses *Google GSON* [8] for all data storage. It is usually faster and more robust than the previously used, dated object serialization provided by Java. For efficiency reasons, all files are compressed.

3.6 Space Considerations

Several considerations were taken into account to reduce the amount of space needed during computation. First of all, the primitive data types of java were used wherever possible. This is necessary to reduce the memory footprint of the program, as object instances of 'Integer' or 'Double', for example, use much more memory than their primitive counterparts 'int' and 'double'. In fact, the space consumption using object instances is prohibitive as early tests revealed.

Second, only the most important information is stored internally in the data structures and exported for reuse. The window neighborhoods were originally stored for each window in the window class. As this information can be computed in a fast and convenient manner before calculating the combined p-value and as it is not used before or after this step, the neighborhoods can either be stored separately being created immediately before and removed immediately after using them or they are not stored at all but created on the fly before being used and removed afterwards. This reduced the space consumption by 20%.

Further, all parts of the window and data mapper data structures (and related classes) that can easily be recomputed are removed before exporting them. Windows, for example, store their chromosome as well as their start and end position, the tag count for each replicate, the associated p-values, and the final p-values. Before exporting the state, the tag counts are removed.

In fact, the information stored in the windows accounts for more than 99% of the space permanently used during the computation (5.6 GB compared to 2.4 MB, window offset: 100nt, window size: 400nt). On the other hand, the information taken from each read during window construction and quality assessment contributes considerably to the volatile objects created and removed during computation.

3.7 Results

The analysis shows that the different steps of Sierra Platinum fall into the following categories:

Fast, serial:

- Joining Windows
- Computing the Poisson Distribution
- Computing the Normalized Poisson Distributions
- Determining the Significant Windows
- Converting the Combined P-Value into Q-Value
- Final P-Value Distribution
- Computing the Agreement between the Multi-Replicate and Single Replicate Results
- Computing Narrow Peaks
- Computing Broad Peaks

IO bound, serial:

- Storing the Results

IO bound, parallel:

- Constructing Windows
- Computing Peak Quality

Maximum parallel:

- Computing the Tag Count Distribution
- Scaling Experiments
- Computing Single Replicate P-Values
- Converting P- to Q-Values
- P-Value Distribution
- Establishing Pearson's Correlation between Replicates
- Computing the Combined P-Value

It showed that the best strategy for constructing windows is Chunk Parallel Coherent (Section 3.5.1.4). This strategy is one order of magnitude faster (factor of 54) than the second best solution. Moreover, the best strategy for computing the window neighborhoods is the integrated approach Compute and Use During p-Value Computation (Section 3.5.8). Further, the 'parallel all' strategy is best for the computation of the correlation (Section 3.5.13). Finally, the best strategy for computing the quality of the final peaks is the combination Peak Coherent Parallel – Broad-Narrow – Smart (Section 3.5.21).

All other steps are either fast enough when computing them sequentially or are best computed with maximum parallelism without further optimization. Overall, the fastest combination (using Holm-Bonferroni correction of p-values) for 6 replicates uses 20:03 minutes (offset: 100nt, size: 400nt) and 48:46 minutes (offset: 50nt, size: 200nt), respectively.

Chapter 4

Benchmark Data Set and Quality Measures

4.1 Context

Testing and benchmarking are essential steps while implementing new methods. Hereby, testing refers to the robustness of the new tool: showing that even with erroneous or bad data, the tool still produces reasonable results without crashing. Benchmarking means running a tool with data for which the ideal results are known and then calculating how close the computed results match the ideal results. Typically, benchmarking results are compared to those of existing tools/approaches performing the same task. While data sets for tests are relatively easy to design, designing benchmarking data sets poses a major challenge.

4.2 State-of-the-Art and Gaps

Surprisingly, even though there are tools to simulate the ChIP-seq experiments, no benchmarking data set for peak-calling is published so far. Koohy et al. [20] and Wilbanks et al. [27] already described the problem and therefore compared the peak-calling results of different peak-callers based on the number of shared peaks of 3 different transcription factors.

As benchmarking data sets are not available, workarounds are used, e.g., using real data instead of artificial data. However, for real data the 'ground truth' is often not known and can just be approximated by verifying the results using known information.

Depending on the tools task, this can be done in different ways. If already other tools exist that fulfill the task, it can be asked how much of the results of the other tools can be recovered by the new tool. However, in those cases it is difficult to decide which tool is better, if the results differ.

Another frequent approach is to use experimentally validated results. In the case of differential expression analysis, for example, qPCR results or spike-in genes are used. However, in this case the set of benchmarking data points is very small since experimental verification cannot be done for all genes and also has it's own limitations.

In the case of peak-calls for ChIP-seq data, two other approaches are often chosen. One approach is to show that the resulting peaks are similar to the results obtained when measuring the same with ChIP-chip, i.e., the first step of the experimental procedure is the same but the sequences pulled out are measured with a chip instead of being sequenced. The other approach is to test the tool with a transcription factor for which the binding motif is known. The benchmark consists of testing whether the peaks detected with the new tool contain a binding site according to the binding motif.

For the first approach, recall and false discovery rate can only be estimated since also ChIP-chip does not guarantee to produce perfect results. For the second approach, one can not guarantee that all binding sites are found since the binding motif may be inaccurate and binding site prediction can be erroneous, too. Furthermore, a binding site does not ensure that the measured transcription factor was bound and thus, a peak is expected. In other words, the second approach does not provide an estimate for the negatives.

To be able to compare our new peak-calling method (Chaper 1), we created several benchmarking data sets and propose four statistical measures for assessing the quality of peak-callers. In this chapter, we describe the method for creating these benchmarking data sets, the proposed benchmarking data sets, and the proposed statistical measures for assessing the quality.

4.3 Goal

In general, benchmarking data sets should provide a combination of data sets with the following properties:

Ideal case: Even though the real data sets will never be 'ideal', the ideal case should always be part of a benchmarking data set. A method, which produces wrong/bad results in the ideal case, can not be assumed to produce reliable results for non-ideal data and thus should not be applied to real data.

Single noise case: For any source of noise, there should be at least one data set simulating this type of noise varying the parameters representing this type of noise. In this way, the robustness of the method with respect to the different sources of noise can be estimated.

Multi-noise case: At least one data set should be designed to demonstrate the performance of the tool when different sources of noise appear together in the same data set. Therefore, data sets with a useful combination of different sources of noise should be part of the benchmarking data set.

Real case: A data set, which is inspired by the sources and strength of noise of real data would be desirable.

4.4 Challenges

Major challenges for constructing benchmarking data sets are:

1. to define a model that produces artificial data that looks like real data
2. to define which sources of noise exist
3. to model the sources of noise in the chosen model system

4.5 Benchmarking Data Set Creation

We used ChIPsim [18], an R package to simulate ChIP-seq, to generate a benchmarking data set for peak-calling that shows characteristics of histone modifications such as broad domains. Instead of using the default model, we followed the nucleosome density example in the manual making several changes to adapt it to our purpose. In principle, the simulation procedure consists of the following steps:

1. Generate a genome sequence
2. Generate features using a Markov model
3. Generate a signal density for all features (i.e., how much signal at a specific base is given to the annotated feature)
4. Calculate the read density according to the signal density and the fragment length
5. Sample reads from the genome according to the read density
6. Sample the base quality for each base according to a defined quality distribution
7. Introduce sequencing errors based on base quality

We used this procedure in two configurations, one time to generate the background and one time to generate the experiment data. Some aspects of the configurations stay the same in both. The genome for background and experiment is the same to be able to map the simulated reads to the same genome. We generate ten chromosomes with the lengths as given in Table 4.1. Each chromosome is obtained by sampling as many bases from the set of DNA bases as desired. The Markov chain generating the features requires a length parameter. This length corresponds to the length of the genomic region assigned to the feature. We used a unique feature length of 146 bases for all states, i.e., each feature corresponds to exactly one nucleosome. The fragment length for sequencing follows a normal distribution with a mean of 200 bases and a standard deviation of 4 bases. Furthermore the values were bound to the interval $[150; 250]$. Thus, the simulated fragments will have a length between 150 bases and 250 bases and are on average 200 bases long.

Table 4.1: Chromosome names and lengths

chromosome	length
chr1	$1 \cdot 10^5$
chr2	$2 \cdot 10^5$
chr3	$3 \cdot 10^5$
chr4	$4 \cdot 10^5$
chr5	$5 \cdot 10^5$
chr6	$6 \cdot 10^5$
chr7	$7 \cdot 10^5$
chr8	$8 \cdot 10^5$
chr9	$9 \cdot 10^5$
chr10	$10 \cdot 10^5$

4.5.1 Background data

We generated trivial features using a one state Markov chain as model for the background corresponding to an unspecific antibody. The density was taken from a Γ-distribution with shape parameter $k = 1$ and scale parameter $\theta = 20$. This setting was suggested by the nucleosome density example in the tutorial of ChIPsim. Calculating the read density and sampling the reads are performed as provided by ChIPsim. The read quality is generated either in "high", "mid", or "low" mode, which are explained below. We took the optimal sequencing depth from the tutorial (number of reads equal to 10% of the genome length) and varied it to 1% for under-sequenced and 40% for over-sequenced data, respectively.

4.5.2 Experiment data

A two state Markov chain of order 1 is used to generate the features for the experiment data. One state represents the background, i.e., the noise which is unspecific to the theoretical antibody used. Its characteristics are the same as those of the background data since both represent basically the same, unspecific binding to the chromatin.

The second state represents the experimental data, i.e., the modified histone the experiment wants to measure. Sticking to an example in the tutorial, we used a single parameter Pareto distribution to generate the density for the specific histone. The shape parameter r is set to 5 as in the tutorial. The average density is

calculated based on the background model as

$$avgDens = k \cdot \theta \cdot e \quad (4.1)$$

where k is the shape parameter and θ is the scale parameter of the background Γ-distribution, and e is the enrichment over the background. The optimal enrichment e is set to 5 and is varied to 2 for low and 4 for mid level enrichment, respectively.

Based on the average density, the lower bound for the density is estimated as

$$lb = (r - 1) \cdot avgDens \quad (4.2)$$

The density for each feature is then drawn from the single parameter Pareto distribution with shape r and lower bound lb.

The sequencing depth is set analogously to the background data. The base quality of the sequencing reads are generated in the modes "high", "mid", and "low". We used Poisson distributions bound to the interval $[0; 40]$ to generate the probability for each possible Phred score. In detail, the probability of Phred score ϕ is

$$P(\phi) = P_{Poisson}(40 - \phi, \lambda) \quad (4.3)$$

where λ is specific for the mode. We set $\lambda = 7$ for mode "high", $\lambda = 17$ for mode "mid", and $\lambda = 29$ for mode "low". The probabilities are normalized such that they sum up to 1 in the interval $[0; 40]$.

4.6 Benchmarking Data Sets

As described in the previous section, we generated benchmarking data of different quality. In this section, we give an overview of the benchmarking data sets generated and how they were processed. Additionally to the generated genome and read files, we provide the reference signal, i.e., the gold standard for the peak's location, as annotation file in BED [19] format.

4.6.1 Replicates Generated

The replicates generated can be subdivided according to their type into 'noise free', 'wrong signal', 'and noisy signal'.

4.6.1.1 Noise free

We generated 6 replicates with optimal parameters for experiment and background. These replicates can be used to evaluate the performance in the ideal case as well as for finding optimal parameters for the peak-caller.

4.6.1.2 Wrong signal

Three replicates with different features each are generated. They can be used to test the performance of the peak-caller when ChIP-seq data may be erroneous. This might happen, for example, when the antibodies did not work correctly.

Table 4.2: Parameter settings for the different levels of quality. See Section 4.5 for a description of the parameters.

	low	middle	high
sequencing quality	29	17	7
sequencing depth	0.01	0.1	0.4
enrichment	2	4	5

4.6.1.3 Noisy Signal

Peak-calling can be affected by poor data quality. In particular, low sequencing quality, over-sequencing, under-sequencing, and low signal enrichment may affect the ability to find peaks and the quality of the predicted peaks. We designed two replicates for each combination of low, middle, and high sequencing quality, sequencing depth, and enrichment (see Table 4.2).

In total, we generated 27 replicates of different quality yielding 54 read files; 27 for the experiment and 27 for the background. While the read files are generated in pairs, i.e., always one file for experiment and one file for background with the same characteristic, read files with different characteristics can be combined to replicates as well to study the effect of unequal quality of background and experiment.

4.6.1.4 Post-Processing

Since we do not simulate adapters, the read files of the replicates generated are directly mapped onto the artificial genome generated during simulation. We used `segemehl` [16] to index the genome and map the reads onto the genome. Mapping was performed with 80% accuracy (i.e., 80% of the alignment have to be identical between read and genome) and only the best hit is reported. The resulting `SAM` [22] files are converted into `BAM` [22] files and sorted using `SAMtools` [22]. Afterwards, `SAMtools` is used to remove PCR replicates. We provide the genome fasta files, the `segemehl` index, the read files in `fastq` format, and the PCR duplicate free and sorted `BAM` files and the corresponding `BAM` index files.

4.6.2 Proposed Benchmarking Data Sets

The generated replicates can be combined to different data sets for benchmarking. As a minimal test for peak-calling, we propose the following combinations:

Noise free: 6 noise free replicates—background and experiment data. Using this data shows if the method produces useful results. Methods that perform poorly on this data should not be used for real data.

Noise: 3 data sets with 6 replicates each combining 1, 2, and 3 replicates with noisy signal but good

quality otherwise with 5, 4, and 3 noise free replicates, respectively. This will indicate the impact of experiments resulting in a wrong signal (e.g., failure of the antibody).

Low quality: 2 data sets with 3 and 4 replicates. In each data set, 2 noise free replicates are used together with either 1 or 2 replicates with low sequencing quality and good quality otherwise.

Over-sequenced: 2 data sets with 3 and 4 replicates. In each data set, 2 noise free replicates are used together with either 1 or 2 replicates with a (too) high sequencing depth and good quality otherwise.

Under-sequenced: 2 data sets with 3 and 4 replicates. In each data set, 2 noise free replicates are used together with either 1 or 2 replicates with a (too) low sequencing depth and good quality otherwise.

Low enrichment: 2 data sets with 3 and 4 replicates. In each data set, 2 noise free replicates are used together with either 1 or 2 replicates with low enrichment and good quality otherwise.

Bad: 2 data sets with 3 and 4 replicates. In each data set, 2 noise free replicates are used together with either 1 or 2 replicates with low sequencing quality, (too) low sequencing depth, and low enrichment. These data sets will show the combined effect of the different sources of noise.

4.7 Statistical Measures for Quality Assessment

The benchmarking data sets allow evaluating statistical parameters to assess the quality of the peak-calls produced with the peak-caller chosen. The gold standard provides a list of peaks specified by the genomic location. Based on the gold standard, we propose to calculate the following statistical measures for quality assessment: the number of peaks n_p, the *recall*, the positive predictive value (PPV), and the false discovery rate (FDR).

Let PC be the set of peak-callers and $pc \in PC$ a peak-caller. Let further p_{pc} be a peak detected by peak-caller pc, p^g a peak in the gold standard, and p^g_{pc} a peak in the gold standard found by the peak-caller pc. Then, n_p, recall, PPV, and FDR are defined as:

$$n_p(pc) = |\{p_{pc}\}| \tag{4.4}$$

$$recall(pc) = \frac{|\{p^g_{pc}\}|}{|\{p^g\}|} \tag{4.5}$$

$$PPV(pc) = \frac{|\{p^g_{pc}\}|}{n_p(pc)} \tag{4.6}$$

$$FDR(pc) = \frac{|\{p_{pc}\}| - |\{p^g_{pc}\}|}{n_p(pc)} \tag{4.7}$$

While p_{pc} and p^g are easy to determine, p^g_{pc} needs a careful definition. This definition has to consider the properties of our gold standard:

1. The length of the peaks in the gold standard are multiples of 146bp by construction since all the features generated with the Markov chain have a length of 146bp, i.e., we simulate individual histones rather than peaks. A peak in the gold standard will always start and end with a feature but may include more than one feature. Thus, peaks have a minimal length of 146bp but can extend much further in length.

2. Reads of length 36bp are generated assuming a fragment length of 200bp on average for sequencing. Thus, enrichment will have the same resolution. Therefore, peak-calling is restricted to the resolution given by the fragment length for sequencing and will not reach the 146bp resolution of the features.

As a consequence, gold standard peaks and predicted peaks will not match up completely. Thus, taking a 100% identical peak match as criterion for finding a peak of the gold standard would be too hard and will distort the performance of the peak-caller evaluated. Therefore, we propose to soften the criterion for rediscovering a peak of the gold standard. We motivate our criterion by the following thoughts:

- The minimum peak length is 146bp.
- The resolution expected for one nucleosome is 200bp on average but lies in the interval $[150; 250]$.
- Given the 146bp resolution, even a perfect prediction would cover only up to 73% (= 146/200) on average. However, the coverage might be as low as 58.4% when the sequencing fragment length is 250bp.
- Not only perfect matches should be counted.
- Incompletely found peaks should also be counted as "found" when most of the peak is found.

Therefore, our proposed soft criterion is a reciprocal overlap of at least 50% between prediction and gold standard:

$$p^g_{pc} := \{p_{pc} | \exists p^g : \text{reciprocal overlap}(p^g, p_{pc}) \geq 0.5\} \tag{4.8}$$

With this criterion, it is now possible to calculate the assessment parameter for any peak-caller on the gold standard benchmarking data sets.

Chapter 5

Evaluation

5.1 Introduction

We present the results of an evaluation of Sierra Platinum. In the main manuscript, we compared the results of Sierra Platinum to other peak-calling methods using the benchmarking data described in Section 4.6 together with the statistical measures described in Section 4.7. The peak-calling methods are presented in Section 5.2.

Here, we show, how different types of noisy data can be recognized using the quality measure visualizations of Sierra Platinum (Section 5.3). Afterwards, we evaluate the influence of the parameter settings of Sierra Platinum on the quality of its peak-calling results (Section 5.4).

5.2 Approach

We compared our results using Sierra Platinum to the results of the following methods:

PePr The multiple-replicate peak-caller PePr v1.0.1 [29].

MACS-SA For each of the replicates, we started MACS2 v2.1.0 [30] in the 'callpeak' mode and called broad peaks. These peaks were then merged to obtain the final resulting peak list.

MACS-CR We used SAMtools [22] to merge the bam files of the experiments of the replicates into a single experiment bam file and the bam files of the background of the replicates into a single background bam file. MACS2 v2.1.0 [30] was started as for the single replicates.

If not stated explicitly, the parameter settings were the same for all peak-calling methods: window size = $200nt$ (equal to the fragment size), window offset = $50nt$, p-value cut-off = 10^{-5} and peak type = 'broad'.

5.3 Sierra Platinum Quality Measures and Visualizations

In the main manuscript, we showed that down-weighting or deleting noisy replicates is beneficial for the performance of Sierra Platinum. While we know which replicates have good quality and which replicates have bad quality in the case of the benchmarking data sets, for real data this information is commonly not available. However, we assess the quality of the replicates using the quality measures and visualizations included in Sierra Platinum. Several visualizations showing different quality measures of the replicates allow the user to judge whether a replicate can be used for peak-calling, should be excluded from peak-calling, or should be down-weighted during peak combination. In this section, we will show, how the different types of noise can be recognized using the visualizations provided by Sierra Platinum.

5.3.1 Noise-free data

Figure 5.1a shows a noise free replicate. The theoretical distributions (at least those calculated for the normalized ones) are (almost) identical (top and bottom, left). Each tag distribution has a single peak and the peak of the experiment is at lower tag counts than the peak of the background distribution (top, middle). However, the respective means of the two distributions are almost identical (location of the peak of the theoretical distribution). The "boxes" of the boxplots are in the green area (top, right). The replicate specific distributions for p-values (bottom, middle) and significant windows (bottom, right) do not differ much from the corresponding median distributions. The correlation with other replicates is high and thus, red is the predominant color in the heatmap (Figure 5.1b). All significant windows of the replicates overlap largely and to approximately the same amount with the final significant windows (Figure 5.1c, right).

5.3.2 Pure Sequencing Quality

Replicates with low sequencing quality are easily recognizable since the "box" of the boxplot is not in the green area and may even drop into the red area of the boxplot (see Figure 5.2a, top, right). However, also the distribution of the significant windows and the amount of overlap with the final significant windows indicate the low sequencing quality. On can observe that the number of significant windows is lower than on average (see Figure 5.2a, bottom, right) and the amount of overlap with the final significant windows is low (see Figure 5.2b, right).

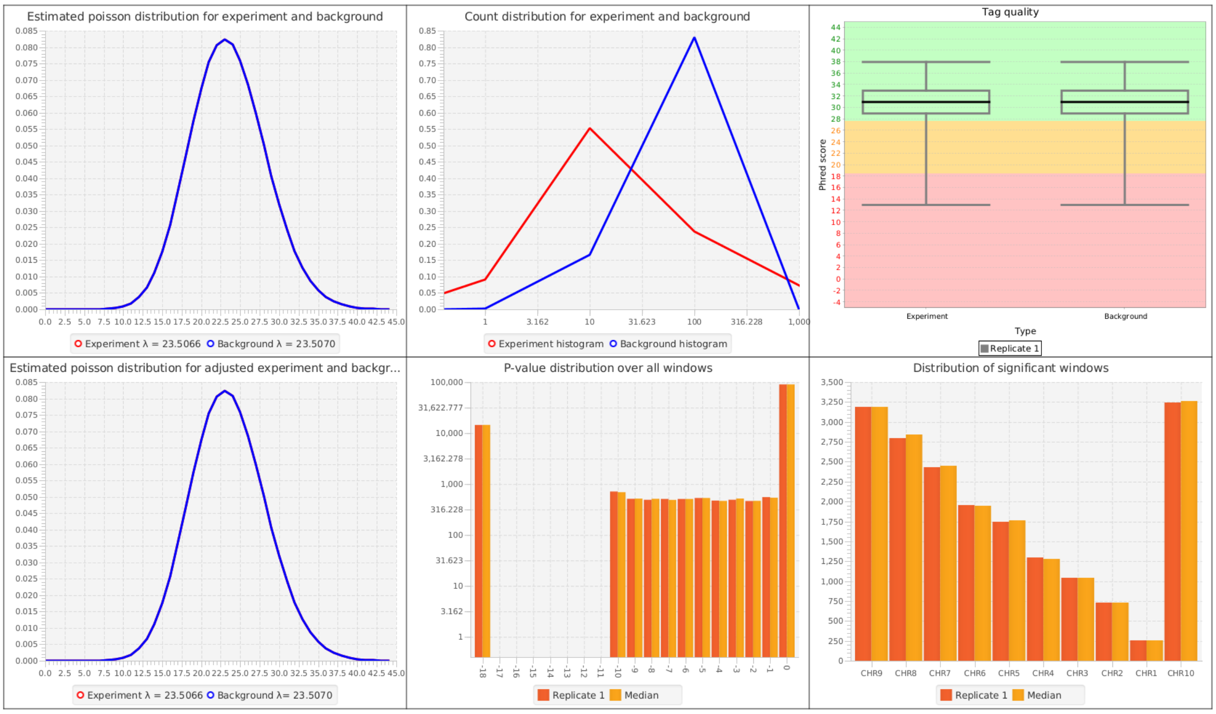

(a) Replicate view.

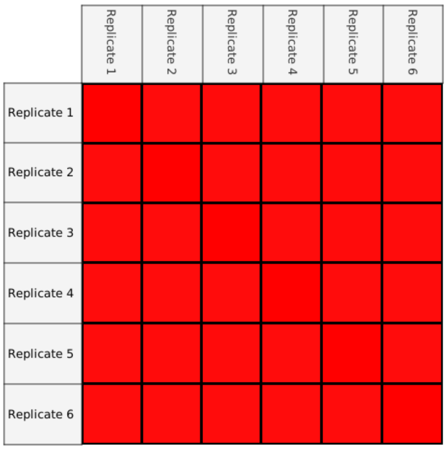

(b) Heatmap.

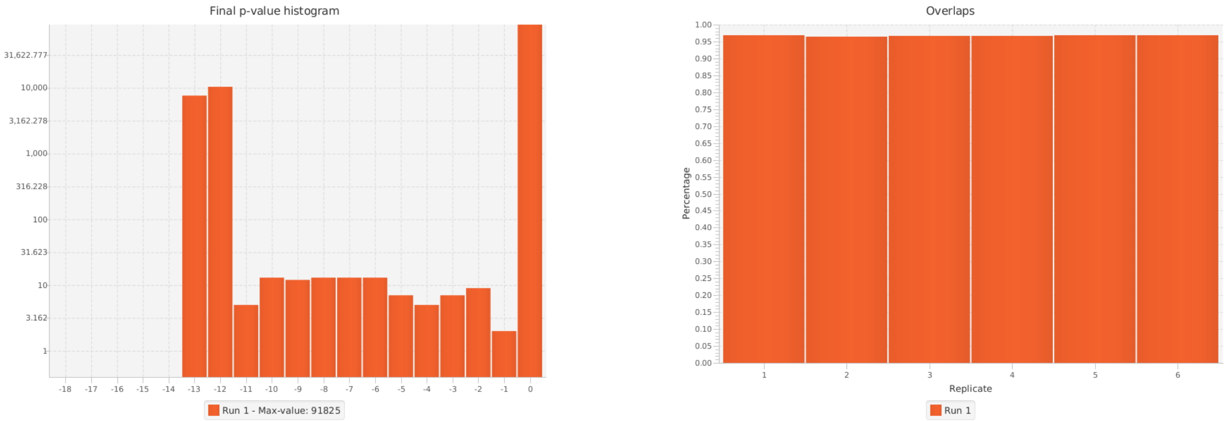

(c) Result charts.

Figure 5.1: Quality measurements for a noise free replicate.

5.3.3 Low enrichment

For replicates with a too low signal-to-noise ratio, e.g., due to ineffective antibodies, the visualizations look like those in Figure 5.3. Low enrichment usually leads to an unclear peak in the experiment tag count distribution, i.e., there is no peak but a plateau (Figure 5.3a, top, middle). Furthermore the significant windows are less frequent in the replicate than in the median distribution of significant windows (Figure 5.3a, bottom, right). All other quality measurements and visualizations in the single replicate view are similar to those of the good replicates. The result charts show a lower amount of overlap for low enriched replicates with the final significant windows (Figure 5.3b, right).

5.3.4 Low Sequencing Depth

Under-sequencing of the experiment leads to peaks close to zero in the tag count distribution (Figure 5.4a, top, middle) since not enough reads are sampled from the data to cover the genome. Under these circumstances, it is hard to reliably estimate the parameter of the background model which affects the peak-calls. The number of significant windows in the replicate is much lower than that in the median replicate (Figure 5.4a, bottom, right) and the p-value distribution of the replicate differs strongly from that of the median (Figure 5.4a, bottom, middle). In particular, there are more windows with p-values close to one but less with p-values close to zero in the replicate compared to the median of all replicates. The correlation with the other replicates is lower (Figure 5.4b, less saturated squares) and the overlap with the final significant windows is very low (Figure 5.4c, right). Furthermore, the p-value distribution of the final p-values has a bathtub shape with a large amount of very low and very high p-values, while there are only few windows with p-values between the lowest two and highest p-value (Figure 5.4c, left).

5.3.5 High Sequencing Depth

When replicates are over-sequenced, the tag count distributions have their peak at almost the same position and low tag counts are particularly rare (Figure 5.5a, top, middle). For most p-values in the p-value distribution the window count is lower than in the median replicate (Figure 5.5a, bottom, middle). Furthermore, high sequencing depth might lead to artifacts. Figure 5.5b, right, shows, for example, that the significant windows of replicate 3 and 4 overlap to 100% with the final significant windows. However, from the distribution of the good replicates in Figure 5.1 we learned that even with perfect data the overlap is not 100% but slightly below 100% since the input signals differ. Likewise under-sequencing, also over-sequencing results in a strongly bathtub-shaped distribution of the final p-values (Figure 5.5b, left).

5.3.6 Noisy Data Sets

When something went completely wrong during the experimental procedure or when the antibody did not work correctly, this would result in a wrong signal. Then, a replicate does not reflect the same signal as the other replicates, This can be recognized using the heatmap. No or very low correlation with all other replicates indicates a wrong signal (Figure 5.6a, white or red with low saturation). Furthermore, the overlap with the final significant windows is also low (Figure 5.6b, right) and the final p-value distribution is that extremely bathtub-shaped that only three different p-values are observed, namely the lowest two and the highest p-value (Figure 5.6b, left).

5.3.7 Summary

Sierra Platinum provides a wide range of visual quality controls. They allow judging, how well a replicate is suited for peak-calling in general and how well it fits to the other replicates. Hereby, each quality control provides insights into specific aspects of the data such as tag distributions or overall significance. This allows not only to identify replicates with poor quality but also the identification of the type of noise. Thus, the user of Sierra Platinum is able to react adequately by deleting or down-weighting certain replicates or by taking lower quality into account for further analysis of the data.

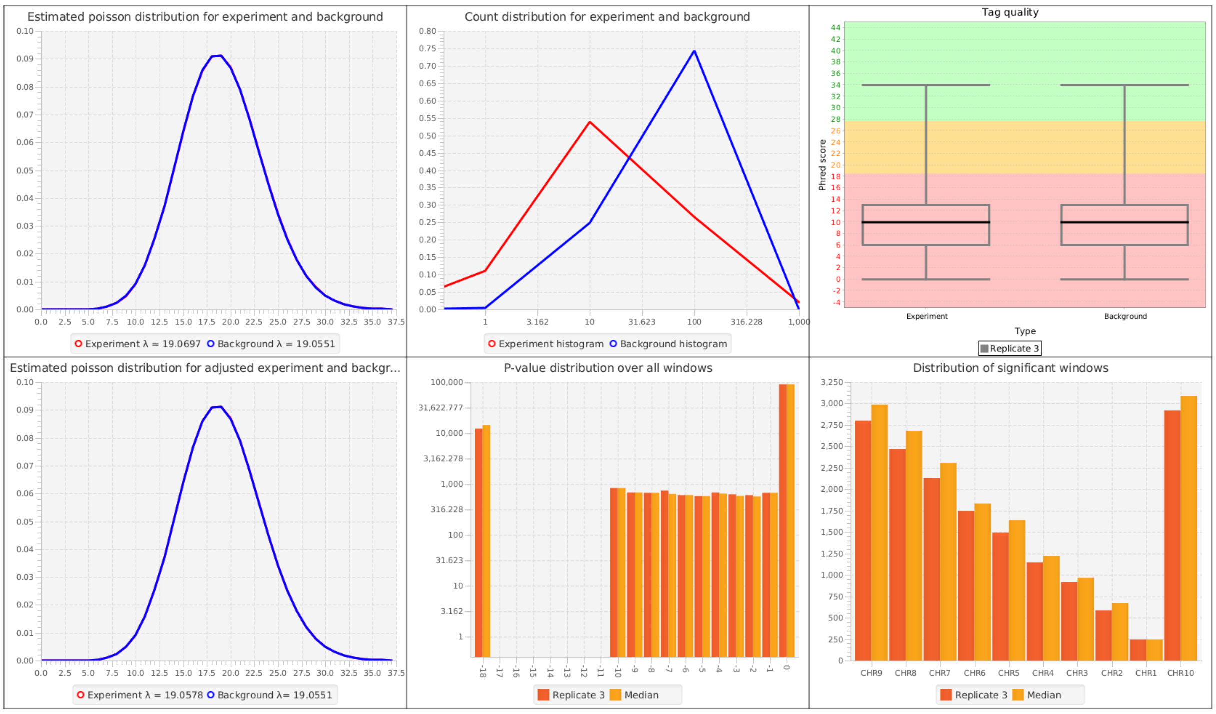

(a) Replicate view.

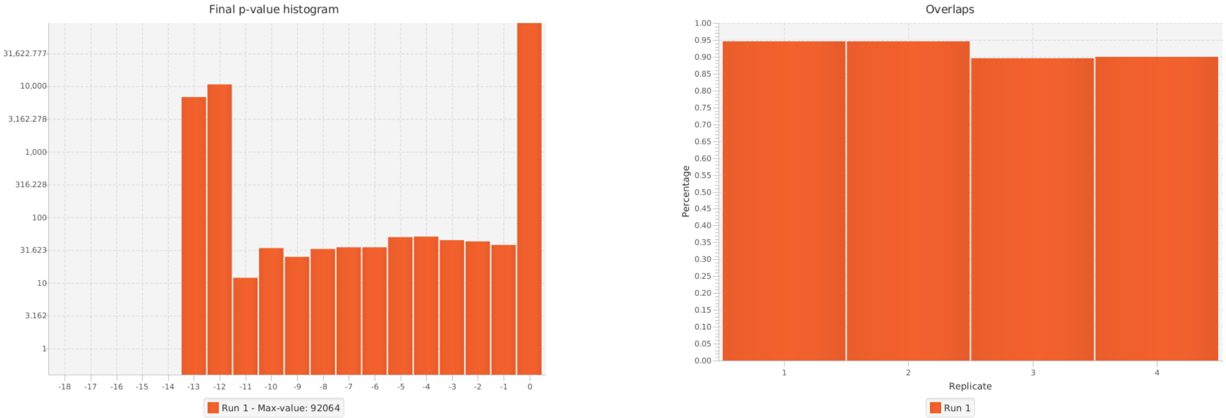

(b) Result charts.

Figure 5.2: Quality measurements for a replicate with low sequencing quality

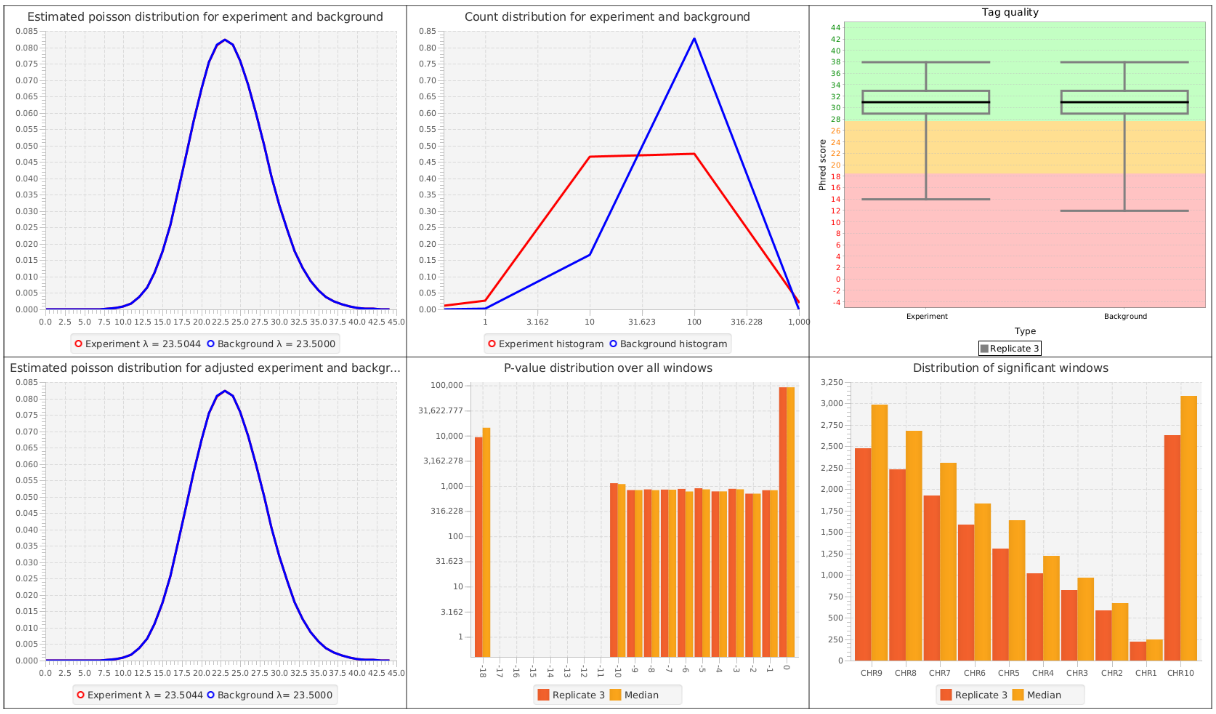

(a) Replicate view.

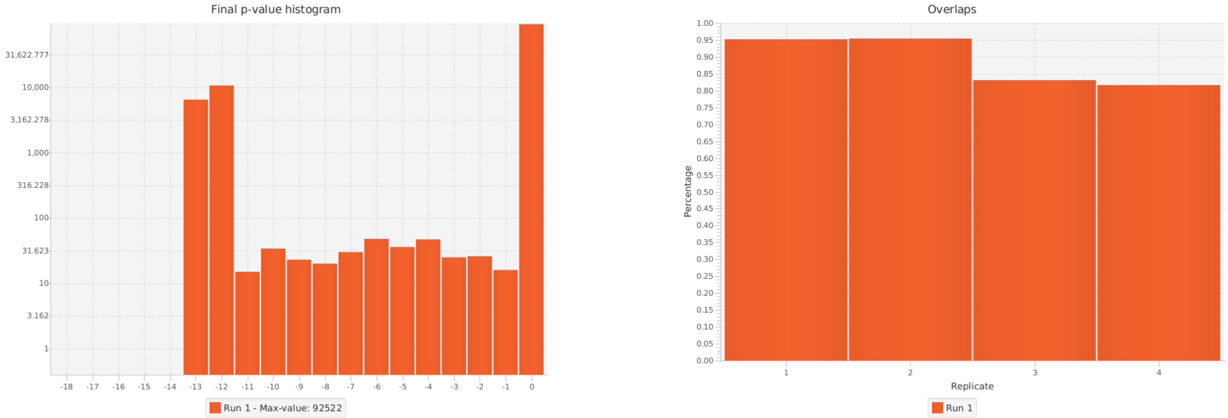

(b) Result charts.

Figure 5.3: Quality measurements for a replicate with low enrichment

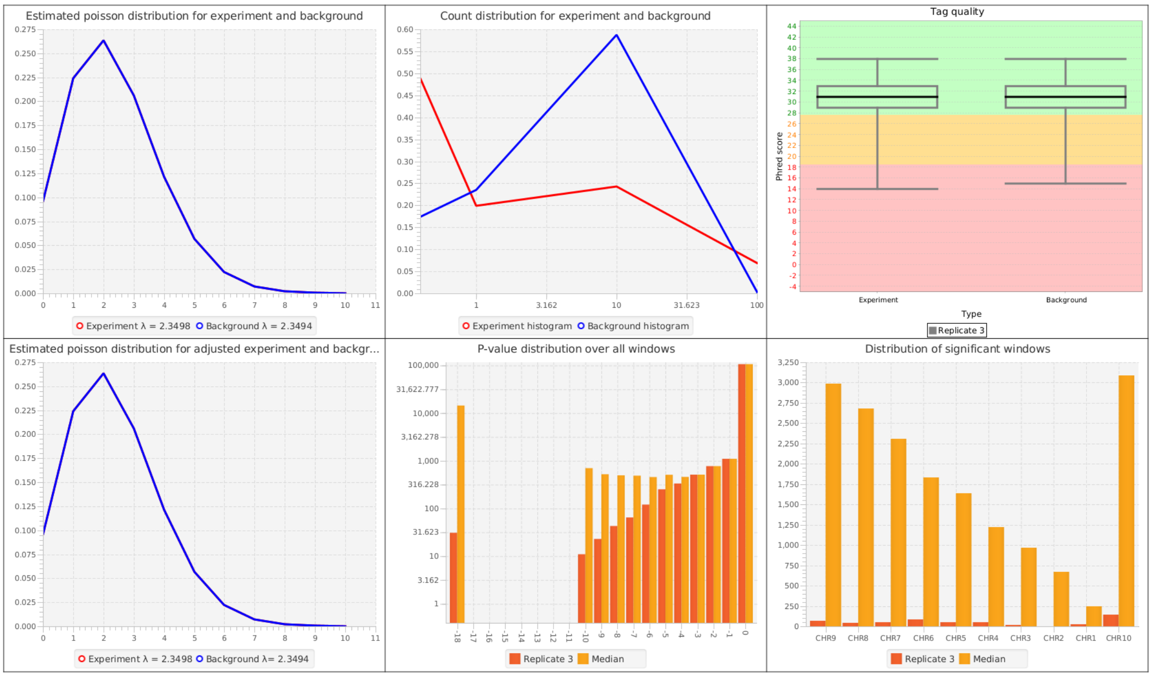

(a) Replicate view.

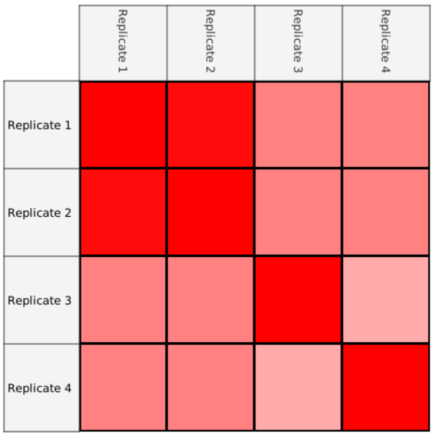

(b) Heatmap.

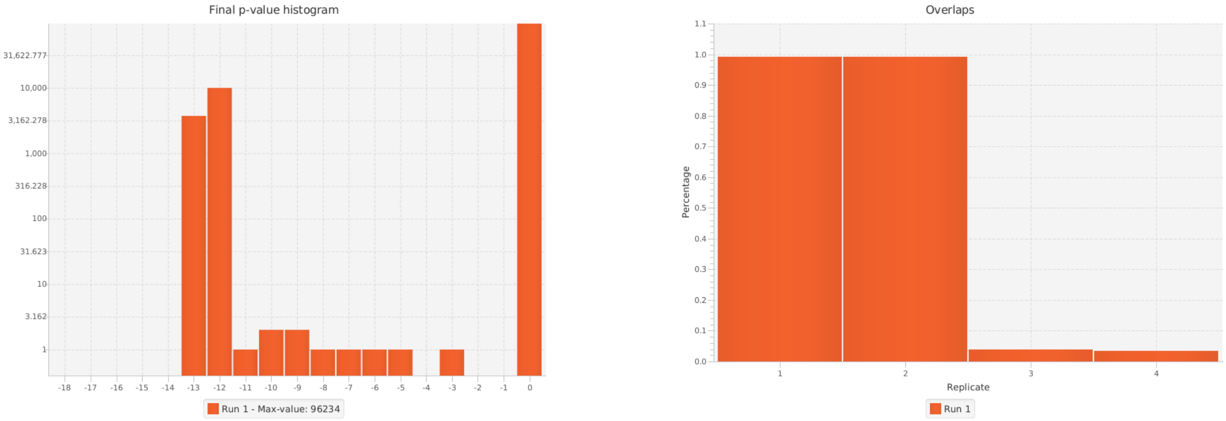

(c) Result charts.

Figure 5.4: Quality measurements for an under-sequenced replicate

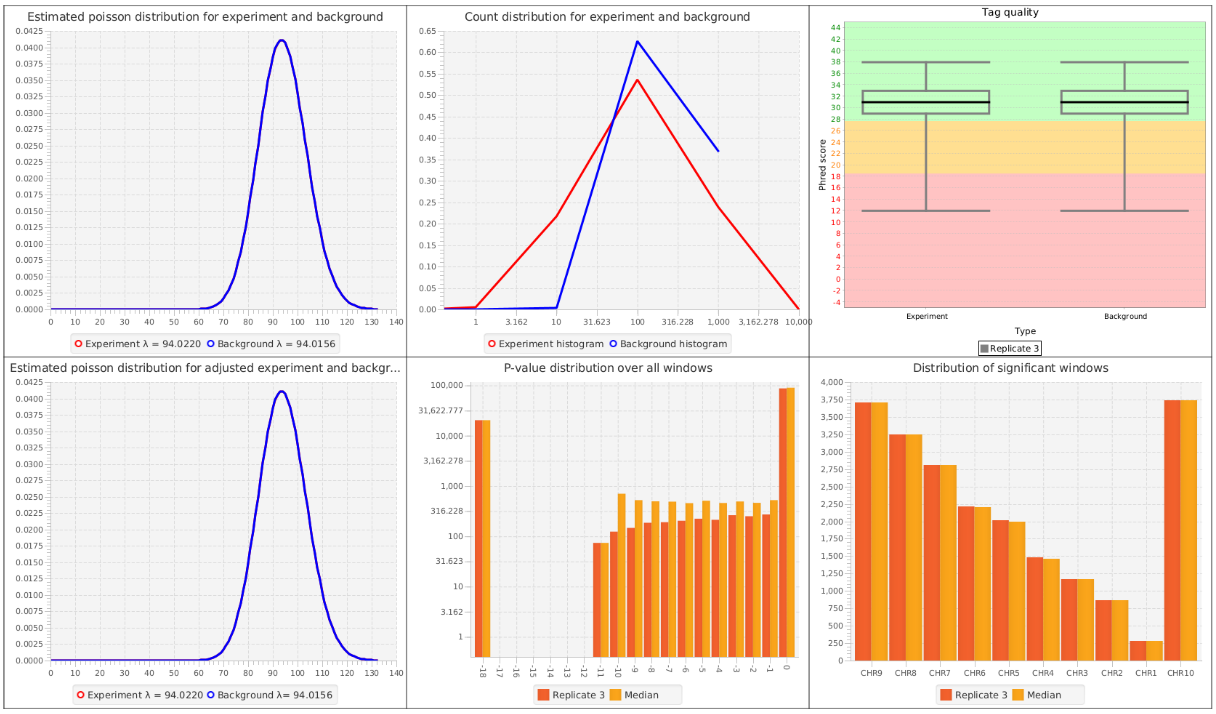

(a) Replicate view.

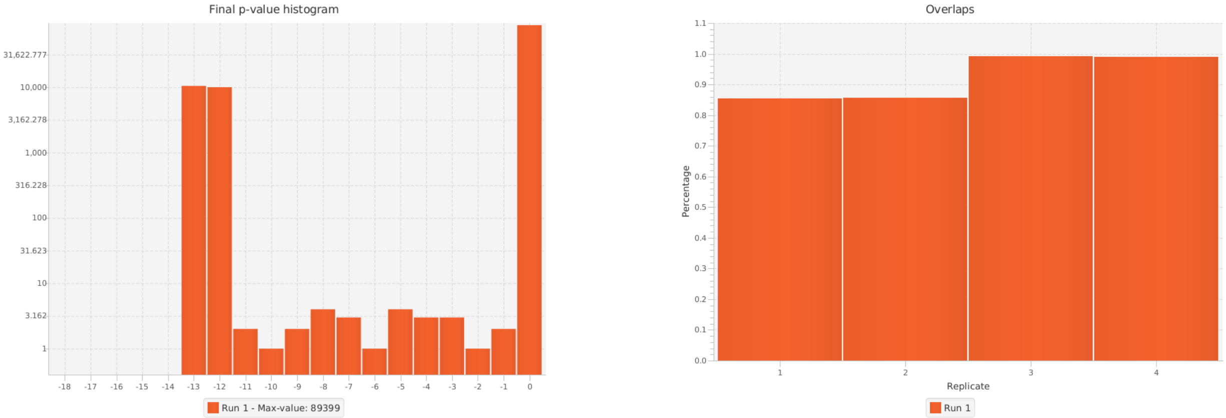

(b) Result charts.

Figure 5.5: Quality measurements for an over-sequenced replicate

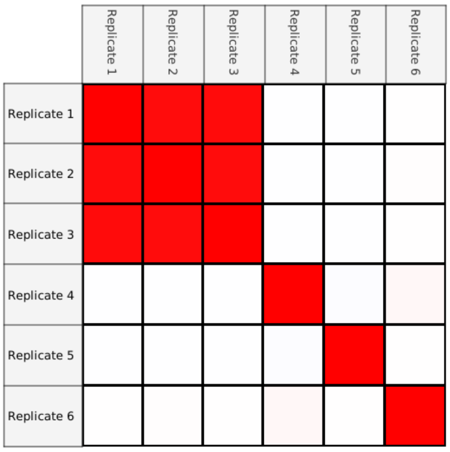

(a) Heatmap.

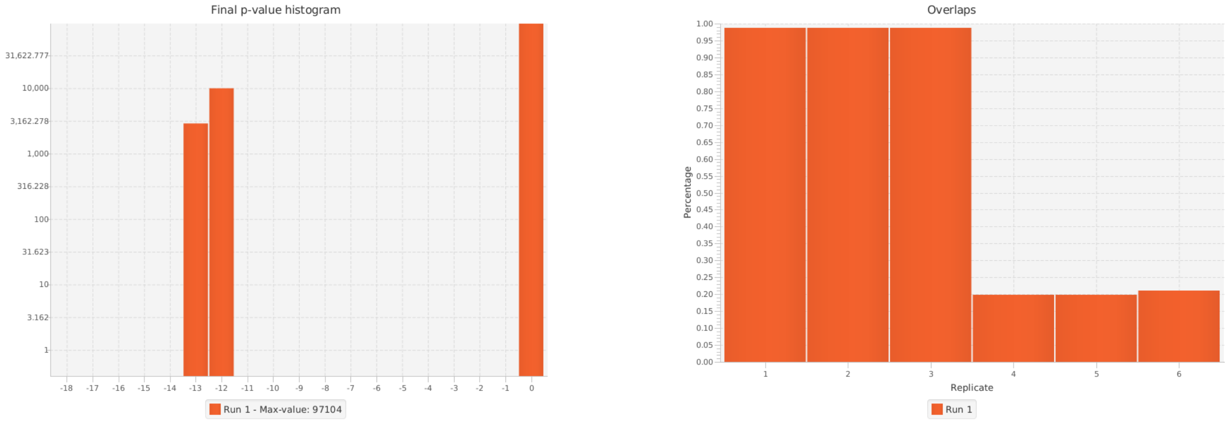

(b) Result charts.

Figure 5.6: Quality measurements for a replicate having the wrong signal

5.4 Parameter Selection

For obtaining optimal results, peak-callers frequently allow to change parameters like window size, window offset, or cut-off value that have a direct impact on the outcome of the peak-calling process. Moreover, most peak-caller provide either default settings or estimates for the parameters provided. We think that default settings are a good starting point for initializing the parameters of a peak-caller. Estimates for the parameters based on the data are helpful for good quality data where such estimates are reliable. We think that an evaluation of different parameter settings enable the user to judge by himself/herself how to choose the parameters. Therefore, we will show the effects of different parameters for the p-value cutoff, the window size, the window offset, and the method for calculating the q-value on the peak-calling process. The three data sets evaluated are the noise-free data set, the data set with with 3 noisy replicates, and a bad quality data set.

5.4.1 How to choose the p-value cutoff

The effect of the p-value cutoff is almost negligible (see Figure 5.7). Using a cut-off value of 1 produces only significant windows. Thus, there is only one peak on each chromosome covering the whole chromosome. Those peaks do not meet our criteria of the reciprocal coverage and thus, result in a recall and PPV of 0 and a FDR of 1. There is also only a very small variation in the recall down to a cut-off value of 10^{-4}. Therefore, we recommend a cutoff of 10^{-5} to be sure to obtain best recall, PPV, and FDR.

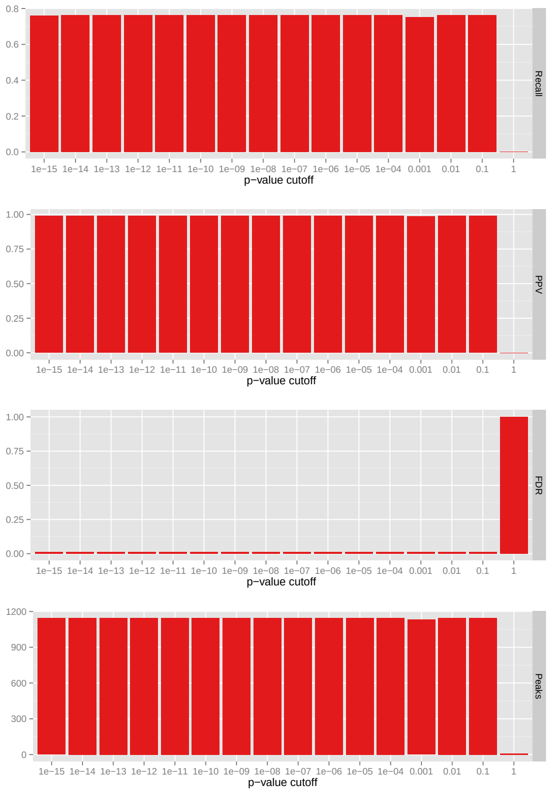

Figure 5.7: Evaluation of the p-value cutoff on the result quality for the noise-free data set.

5.4.2 How to choose the window size

Furthermore, we tested the influence of the window size on recall, PPV, and FDR. Note, that the fragment size of the simulated data is on average 200nt. To assure that the p-value cutoff is independent of the other parameters, we not only tested different window sizes but also varied the p-value cutoff. With respect to the recall (see Figures 5.8a), we observe that window sizes between 100 to 200 nt have the best recalls for good quality data (with 200nt being the very best). For the noise-3 and the bad-2 data set (see Figure 5.8b, 5.8c) larger window sizes yield better recalls. In this cases, the best recall can be achieved with a window size of 300nt. A similar behavior can be observed for the positive predictive value (see Figure 5.9) and the false discovery rate (see Figure 5.10).

We can understand the reasons for the observed FDRs, PPVs, and recalls when analyzing the number of peaks (see Figure 5.11). Small window sizes lead to an overprediction of the number of peaks in the case of the noise-free data set. It seems that some of the small windows are insignificant due to local sequence composition biases and thus lead to a gap in the peak. Such insignificant windows are less likely with large window sizes. In the case of bad quality data, the number of predicted peaks is too low for small window sizes. A reason therefore might be a low signal due to low enrichment of chromatin fragments during the experiment in combination with local sequence composition biases. Given the results, we recommend a window size equal to the average fragment length (200nt in our case). If it is obvious that the data has bad quality, a larger window size should be chosen.

5.4.3 How to choose the window offset

To capture the effect of different offsets in combination with the window size and the p-value cutoff, we evaluated Sierra Platinum for 4 different window offset settings for each window size. Each combination of window size and offset was tested with 5 different p-value cutoffs. As window offset, we choose one quarter, one half, three-quarters, and seven-eighths of the window size. The results are shown in Figures 5.8–5.11.

For small window sizes (50–125 nt), we see that small offsets (one quarter of the window size) give the best results (highest recall and PPV, and lowest FDR). The window offset for the proposed window size of 200nt does not strongly affect PPV, FDR, or recall on the noise-free data set. Nevertheless, small offsets (one quarter or one half) are slightly better than large offsets. For bad quality data, one quarter window offsets always produced the best results with respect to recall, PPV, FDR, and number of predicted peaks. Consequently, our recommendation for the window offset is one quarter of the window size.

5.4.4 Which method for the q-value calculation should be used

We provide different methods to compute the q-value. The most traditional but also most strict method is the Holm-Bonferroni method. Both methods from Storey are less strict and thus more suitable for noisy data where very strict methods may lead to non-significant windows only. Storey proposes both a simple and a bootstrap version. The latter one is more robust. We tested all three methods with window size 200nt, offset 50nt, and two different cutoff values (1e-5 and 1e-10). The results are shown in Figure 5.12.

On very noisy data, the Holm-Bonferroni correction performs best regardless of the cut-off value. Storey's less strict methods lead to high FDRs and low recall and PPV in those cases. On data sets with replicates of bad quality Storey's methods for the calculation of the q-value perform best. The bootstrap method outperforms the simple estimate (as expected). Nevertheless, for very skewed distributions, the bootstrap method might not be able to produce an estimate for the height of the false positives. On noise-free data, the recall is almost equal between the different methods but the FDR is lower for Holm-Bonferroni correction.

5.4.5 Summary

Sierra Platinum has four parameters to fine tune the performance of Sierra Platinum. The nature of these parameters implies that a bad parameter choice can strongly affect the quality of the results. We showed, how to adapt the parameters of Sierra Platinum optimally to the given data sets.

The window size should be about the size of the fragments in the experiment. If the fragment size varies across the replicates or is completely unknown, we suggest shorter windows rather than longer ones except if the data quality is overall very low when larger window sizes perform best. The offset should be one quarter of the window size in any case. The p-value cut-off does not affect the performance strongly. However, we suggest a default p-value of 10^{-5} since for higher p-value cut-offs variation in their performance could still be observed. The last parameter is the method for the q-value calculation. We showed that performance differences between the different methods depend on the type of noise in the data and on the chosen p-value cut-off for significance. Holm-Bonferroni corrections perform well and fast in most cases but can not handle combinations of noise very well. However, for those cases Storey's q-value methods perform well and robust.

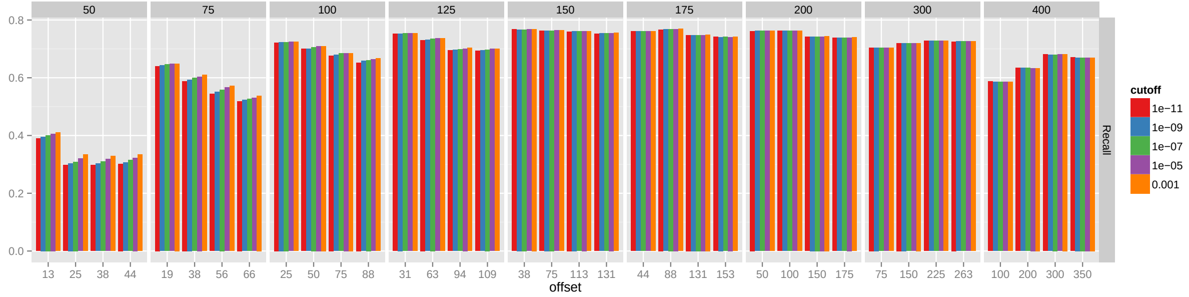

(a) Noise-free data set

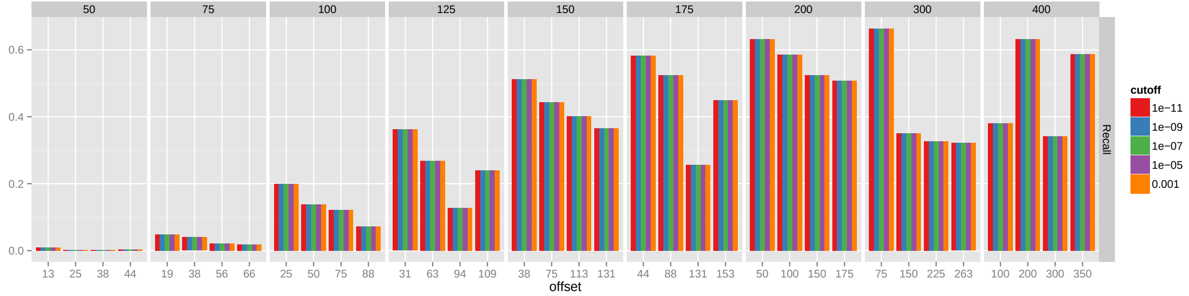

(b) Noise-3 data set

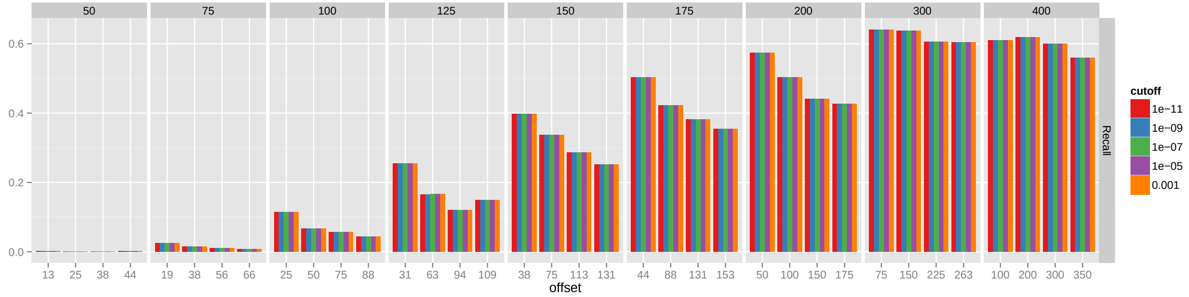

(c) Bad-2 data set

Figure 5.8: Recall of the peak-calls using different combinations of window size, window offset, and cutoff.

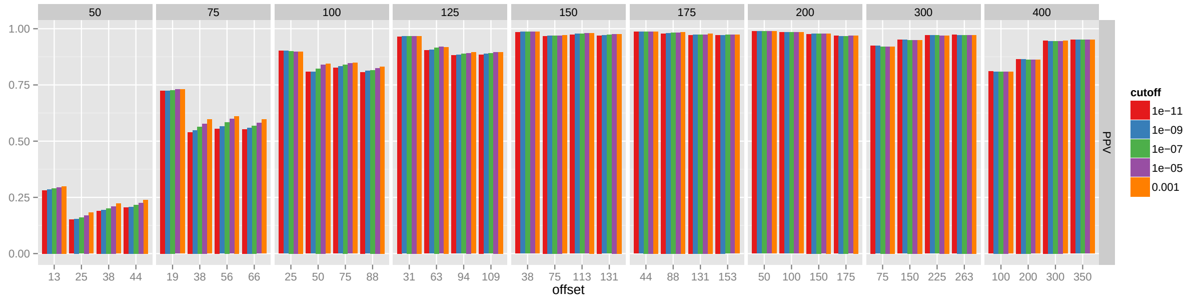

(a) Noise-free data set

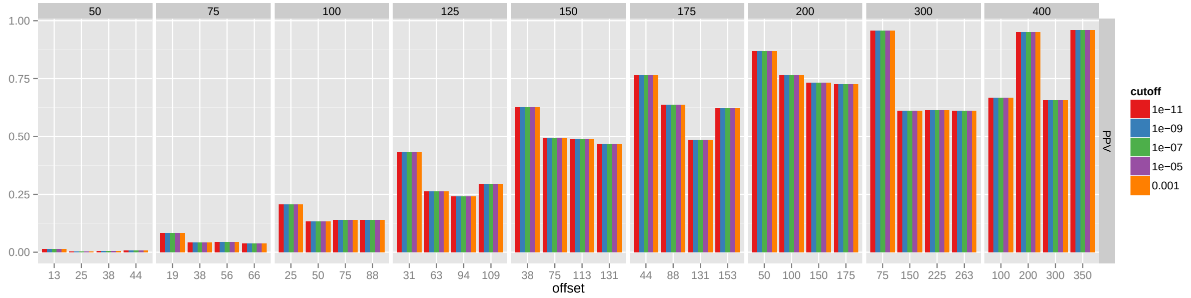

(b) Noise-3 data set

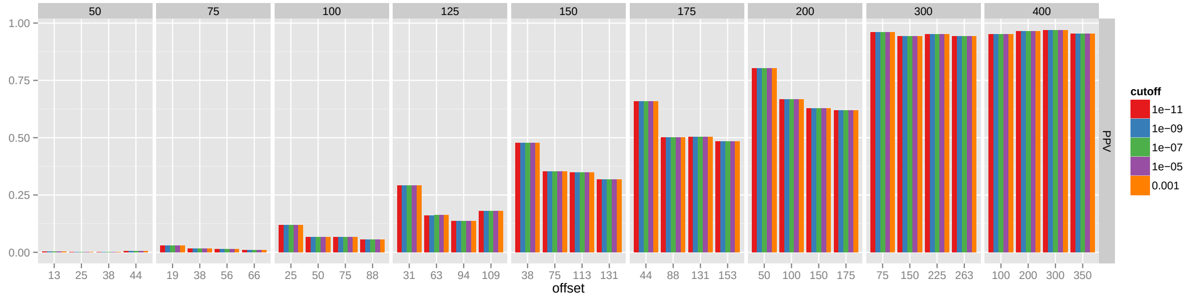

(c) Bad-2 data set

Figure 5.9: Positive predictive value of the peak-calls using different combinations of window size, window offset, and cutoff.

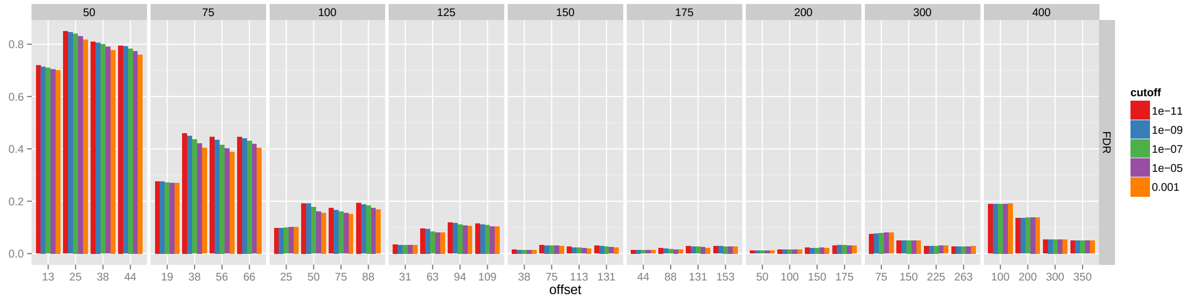

(a) Noise-free data set

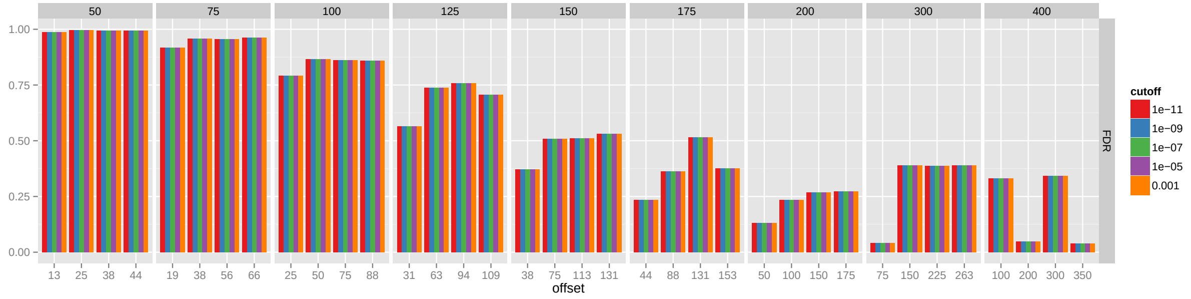

(b) Noise-3 data set

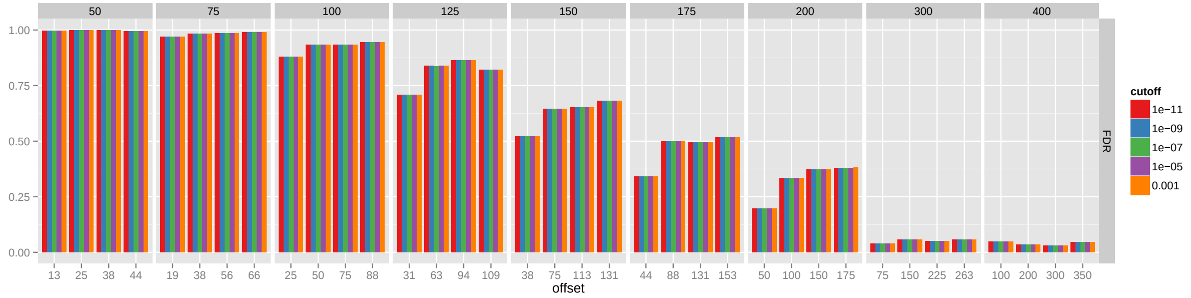

(c) Bad-2 data set

Figure 5.10: False discovery rate of the peak-calls using different combinations of window size, window offset, and cutoff.

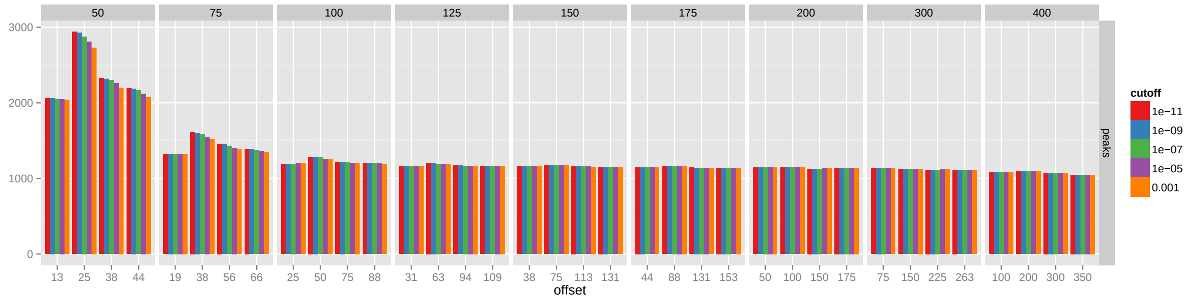

(a) Noise-free data set

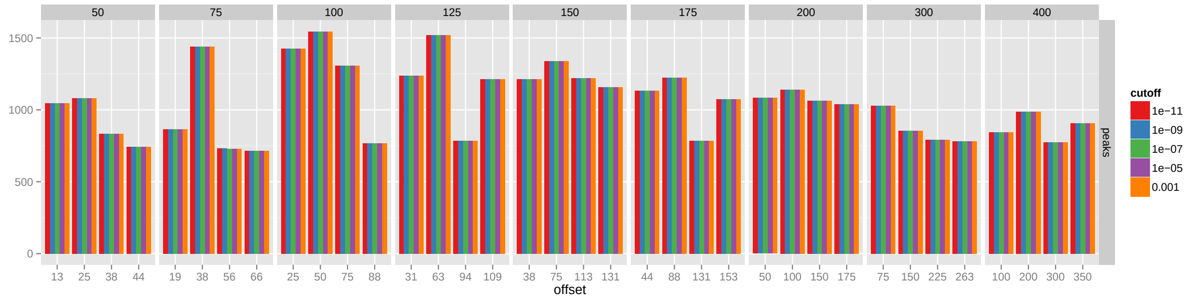

(b) Noise-3 data set

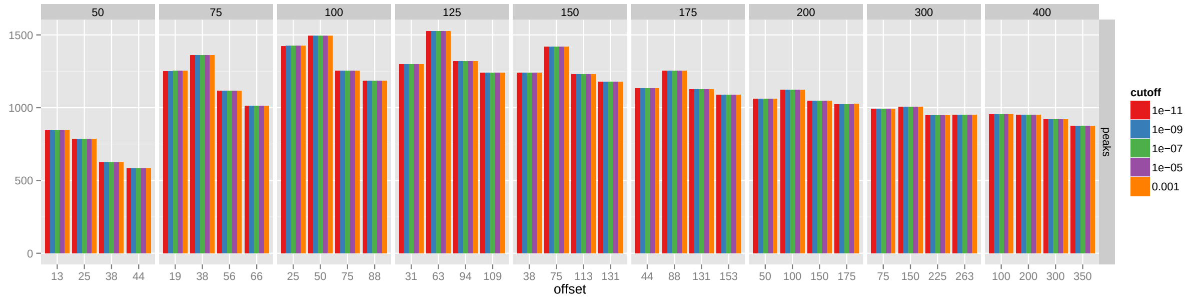

(c) Bad-2 data set

Figure 5.11: Number of peaks found using different combinations of window size, window offset, and cutoff.

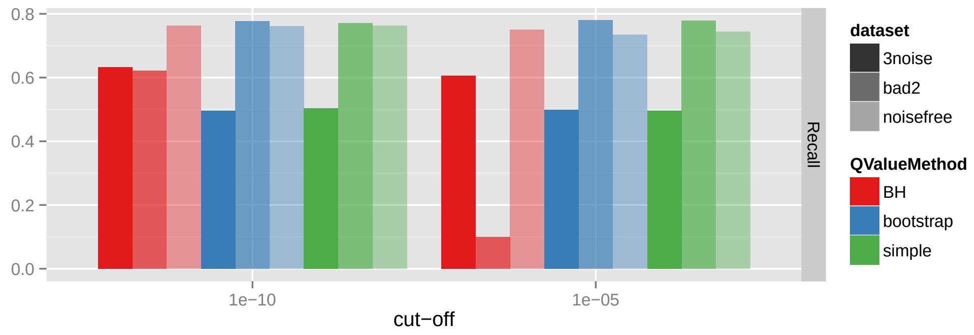

(a) Recall

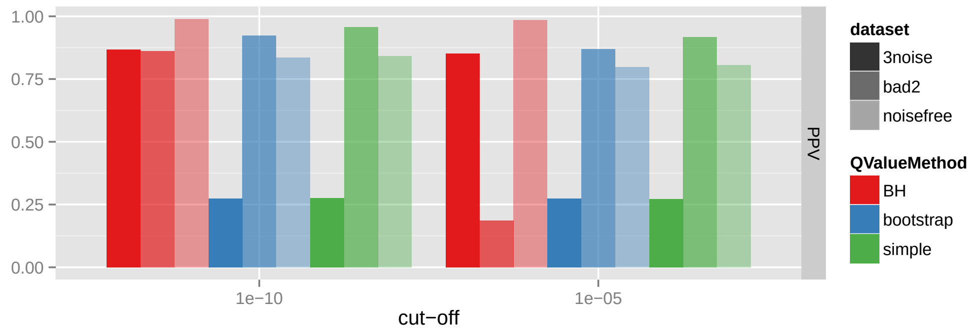

(b) PPV

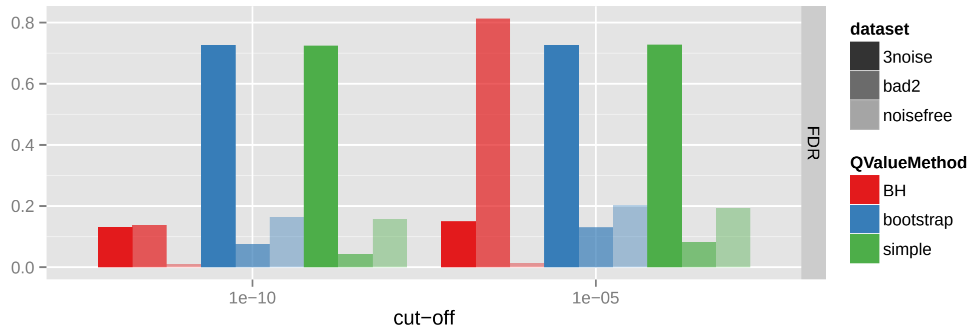

(c) FDR

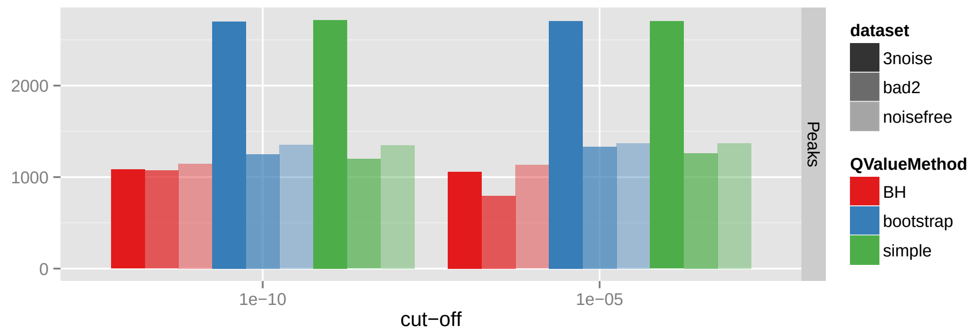

(d) Number of peaks found

Figure 5.12: Evaluation of the q-value methods on noise-free, bad quality replicates (2 noise-free and two under-sequenced replicates with low enrichment and low quality), and 3 noisy data sets (6 replicates in total, 3 are noisy) combined with different cut-off values for the q-value.

Chapter 6

Results

6.1 Data Sets

6.1.1 Reference Data

As reference we use the peak-calls for the epigenome E003 that were generated by the NIH Roadmap Epigenome project [24]. We downloaded the consolidated broad peaks for the embryonic stem cell line H1.

According to the Roadmap Epigenomics Consortium [24], the 'MACS-CR approach' (Section 5.2) was used to obtain the peak calls for E003. In this approach, first all libraries for H1 are down-sampled to 30M reads per sample. Next, libraries measuring the same modification belonging to the input were merged into one file. Afterwards, MACS2 was applied on this data to generate the peak-calls for the different modifications.

6.1.2 Input Data of Sierra Platinum

6.1.2.1 Data Set for H1

As test data set for Sierra Platinum, we use the replicates of the H1 cell line (an embryonic stem cell line) from the NIH Roadmap Epigenomics Project [24]. We downloaded the raw data for five histone modifications—H3K4me3, H3K27me3, H3K9me3, H3K27ac and H3K9ac—and the corresponding ChIP-input as background, if available. Table 6.1a provides the GEO identifications and the sequencing center, which produced the data set. An ID for each replicate is given for further reference to the replicates.

6.1.2.2 Data Set for ESCs

As a second test data set (Table 6.2a), we downloaded also the data for all other embryonic stem cell lines available at the NIH Roadmap Epigenomics Project. For this data set, we downloaded 4 modifications—H3K4me3, H3K27me3, H3K9me3, and H3K27ac—and the corresponding ChIP-Input as background, if available. In any case, we used only those modification data for which a fitting ChIP-input was available. For this data set, we obtain up to 16 replicates for each modification.

We downloaded for each modification and replicate the available sra files from GEO [13, 12]. Then, we converted the sra files into fastq files and clipped the adapters. Afterwards, the reads were mapped using `segemehl` [16] with an accuracy of 80% against the human genome version hg19 [21]. Using SAMtools [22], the results were converted into one bam file for each data set. Since not all embryonic stem cell lines were male, we removed chromosomes X and Y in the data of the second test set. In the cases where the data set consists of multiple fastq files, the mapping results were merged in addition to sorting and indexing them. We used SAMtools to remove PCR duplicates.

6.2 Peak Calls

We used Sierra Platinum to generate peak-calls with a window size of 200nt, a window offset of 50nt, and a p-value cutoff of $1e^{-5}$. Probits were corrected for inter-replicate correlation. The Holm-Bonferroni method is used to calculate the q-value.

6.2.1 Peak Calls for H1

Using the visual quality controls, we analyzed the replicates for H1 according to noise. Given the results, we made the decisions shown in Table 6.1b where the *replicate ID* refers to the ID given in Table 6.1a, the *weight* specifies the weight we used for weighting the replicate, and *off* means that we excluded the replicate.

6.2.2 Peak-calls for ESCs

Using the visual quality controls, we analyzed the replicates for ESCs according to noise. Given the results, we made the decisions shown in Table 6.2b where the *replicate ID* refers to the ID given in Table 6.2a, the *weight* specifies the weight we used for weighting the replicate, and *off* means that we excluded the replicate.

Table 6.1: H1.

(a) Overview of the data sets used. IDs are only used internally to distinguish the different replicates available for H1. For each replicate, we provide the sequencing center, which produced the data, and the GEO identifications for the data sets H3K4me3, H3K27me3, H3K9me3, H3K27ac, H3K9ac, and ChIP-input.

ID	Center	H3K4me3	H3K27me3	H3K9me3	H3K27ac	H3K9ac	Chip-Input
1	UCSD	GSM469971	GSM466734	GSM605325	GSM466732		GSM605333
2	UCSD	GSM605315		GSM605327		GSM605323	GSM605339
3	UCSD			GSM818057			GSM667642
4	UCSD	GSM409308	GSM434776			GSM434785	GSM605334
5	BI	GSM433170	GSM433167	GSM433174		GSM433171	GSM433179
6	BI	GSM537681					GSM537682
7	UCSF-UBC	GSM432392		GSM450266			GSM450270
8	UCSF-UBC			GSM428291		GSM410807	GSM428289
#replicates		6	3	6	1	4	8

(b) Decisions based on visual inspection of the quality of the replicates. *ID*: ID given Table 6.1a, *Weight*: weight used, *off*: replicate excluded, *empty cell*: replicate not available.

ID	H3K4me3	H3K27me3	H3K9me3	H3K27ac	H3K9ac
1	0.1	1	0.05	1	
2	0.1		0.05		1
3			1		
4	1	0.05			0.1
5	0.1	0.1	0.1		1
6	off				
7	1		off		
8			1		off

Table 6.2: ESC.

(a) Overview of the data sets used: IDs are only used internally to distinguish between the different replicates available for embryonic stem cell lines. For each replicate, we provide the sequencing center, which produced the data, and the GEO identifications for the data sets H3K4me3, H3K27me3, H3K9me3, H3K9ac, and the ChIP-input.

Id	Center	H3K4me3	H3K27me3	H3K9me3	H3K9ac	Chip-Input
1	UCSD	GSM409308	GSM434776		GSM434785	GSM605334
2	UCSD	GSM469971	GSM466734	GSM605325		GSM605333
3	UCSD	GSM605315	GSM605308		GSM605323	GSM434785
4	UCSD	GSM616128	GSM706066	GSM667633	GSM616129	GSM667643
5	UCSD			GSM605327		GSM605335
6	BI	GSM433170	GSM433167	GSM433174	GSM433171	GSM433179
7	BI	GSM537681	GSM537683			GSM537682
8	BI	GSM669889	GSM669887	GSM669886	GSM669963	GSM669888
9	BI	GSM669893	GSM669897	GSM669894	GSM669890	GSM669895
10	BI	GSM772978	GSM772977	GSM772856	GSM772980	GSM772913
11	BI		GSM773002		GSM773003	GSM772979
12	BI	GSM669936	GSM669942	GSM772799	GSM670013	GSM772794
13	BI	GSM772797	GSM772766		GSM772796	GSM772755
14	BI	GSM537665	GSM537648	GSM537639	GSM537670	GSM537647
15	UCSF-UBC		GSM428295	GSM428291		GSM428289
16	UCSF-UBC	GSM432392		GSM450266		GSM450270
#replicates		13	14	11	11	16

(b) Decisions based on visual inspection of the quality of the replicates. *ID*: ID given Table 6.2a, *Weight*: weight used, *off*: replicate excluded, *empty cell*: replicate not available.

ID	H3K4me3	H3K27me3	H3K9me3	H3K9ac
1	0.02	1		0.8
2	0.02	0.8	1	
3	1	1		0.8
4	off	0.02	1	0.01
5			1	
6	0.02	0.01	1	off
7	off	off		
8	1	1	1	0.01
9	1	1	1	0.01
10	0.02	1	1	off
11		1		1
12	1	1	1	1
13	1	1		1
14	off	0.02	1	off
15		0.02	1	
16	0.02		1	

6.3 Stem cell markers

In this part, we will have a look at the genes known to be embryonic stem cell marks, i.e., they are active in embryonic stem cells and their activity is crucial for cell identity and function. We will compare the predicted epigenomic states of the promoters of such markers. The epigenomic state is hereby defined as the collection of peaks for the histone modifications analyzed. Three different epigenomic states will be compared. Firstly, the epigenomic state predicted with Sierra Platinum on the H1 data set (see Table 6.1a). Secondly, the epigenomic state predicted with Sierra Platinum on the ESCs data set (see Table 6.2a). Thirdly, the consolidated epigenomic state of the H1 cell lines downloaded from the NIH Roadmap Epigenomics Webportal of the Washington University (epigenome E003, only H3K4me3, H3K27me3, and H3K9me3).

We looked at five stem cell markers for embryonic stem cells: SNF2H, BRG1, SSRP1, OCT4, and SNF5 [23]. The results for SNF5 are presented in the main manuscript and are not repeated, here. Figure 6.1–6.4 show the corresponding genomic locations of the remaining stem cell markers with the peaks annotated. The color coding of the peak tracks in these figures is explained in Table 6.3. The peaks generated by Sierra Platinum for the H1 and the ESC data set largely overlap these regions even though the signal was different. In particular, H3K4me3 (light and middle green) have very similar peaks but also for H3K9ac (light and dark pink) the agreement of H1 and ESCs peaks is large. Comparing the three marks that we downloaded for E003 to H1 and ESCs, we see this agreement only—if at all—in H3K4me3.

6.3.1 SNF2H & BRG1

SNF2H (Figure 6.1) and BRG1 (Figure 6.2) seem to be at least active in E003 (promoter has H3K4me3 mark but no repressive mark). The gene bodies are speckled with H3K27me3 and H3K9me3 peaks suggesting, that there might be repression of transcription in this region. However, according to our predictions for H1 *and* ESCs, there are no repressive marks, neither in the promoter nor in the gene bodies.

Table 6.3: Color coding used in the figures showing the peaks as UCSC tracks at selected genomic positions.

	H1	ESC	E003
H3K4me3			
H3K27me3			
H3K9me3			
H3K27ac			
H3K9ac			

6.3.2 SSRP1

Both promoter regions of SSRP1 are covered with H3K4me3 peaks in all three epigenomic states and H3K9ac as well as H3K27ac also have a peak within the promoter regions (if at all available, Figure 6.3). This is on agreement with the transcriptional activity required for SSRP1 in embryonic stem cells [23]. Again, neither in H1 nor in ESCs (when using Sierra Platinum) we can find H3K9me3 and H3K27me3 marks in the promoter regions or the gene body. Only E003 annotated H3K27me3 in these regions.

6.3.3 OCT4

OCT4 (Figure 6.4), also a marker for embryonic stem cells, is annotated as active (H3K4me3 and H3K9ac marks in the promoter) by the H1 and ESC data sets. It is very unclear whether one would conclude activity or inactivity from the epigenomic state E003. There is a large H3K4me3 peak overlapping the promoter. However, very close to the promoter and along the gene body there are also H3K27me3 marks (repressive). Furthermore, there is also one H3K9me3 mark close to the promoter.

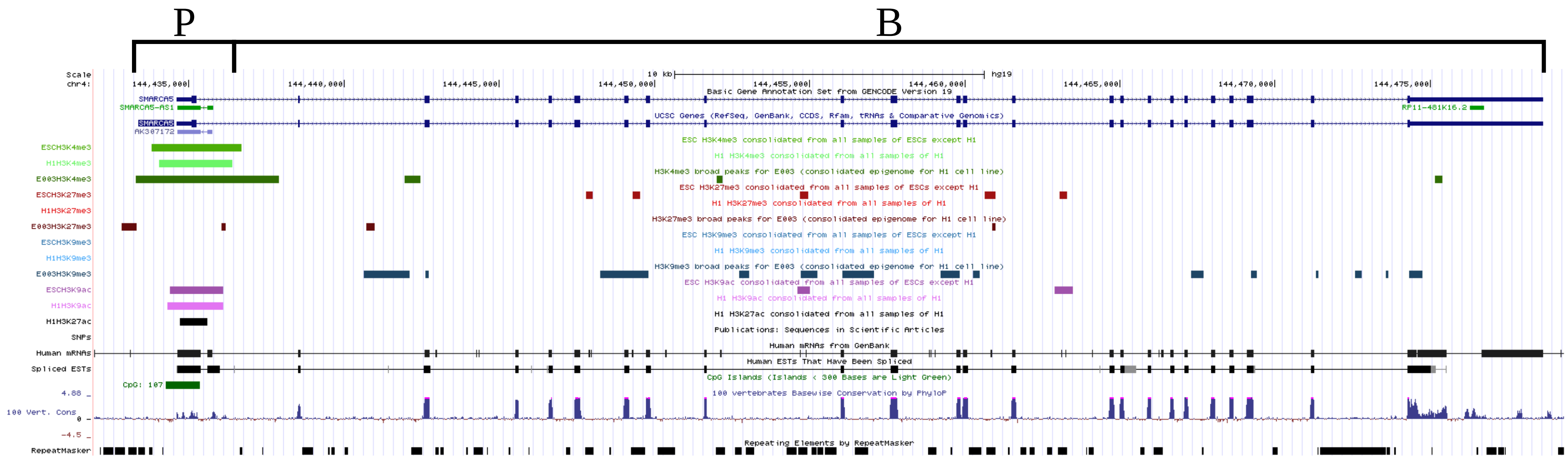

Figure 6.1: Genomic location of SNF2H: The peak-calls are shown below the transcript annotation of SNF2H. Peak-calls for the different modifications and data sets are color coded as described in Table 6.3. The position of the described promoter and gene body are marked with ’P’ and ’B’, respectively.

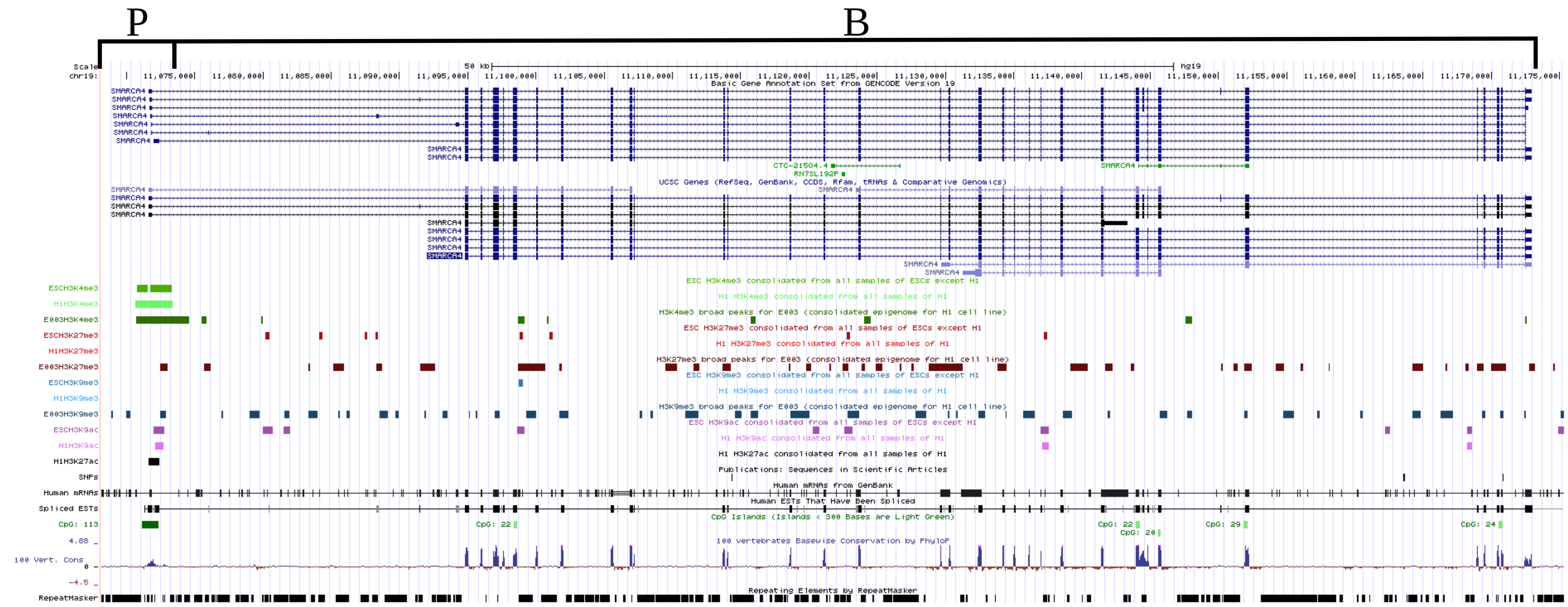

Figure 6.2: Genomic location of BRG1: The peak-calls are shown below the transcript annotation of BRG1. Peak-calls for the different modifications and data sets are color coded as described in Table 6.3. The position of the described promoter and gene body are marked with 'P' and 'B', respectively.

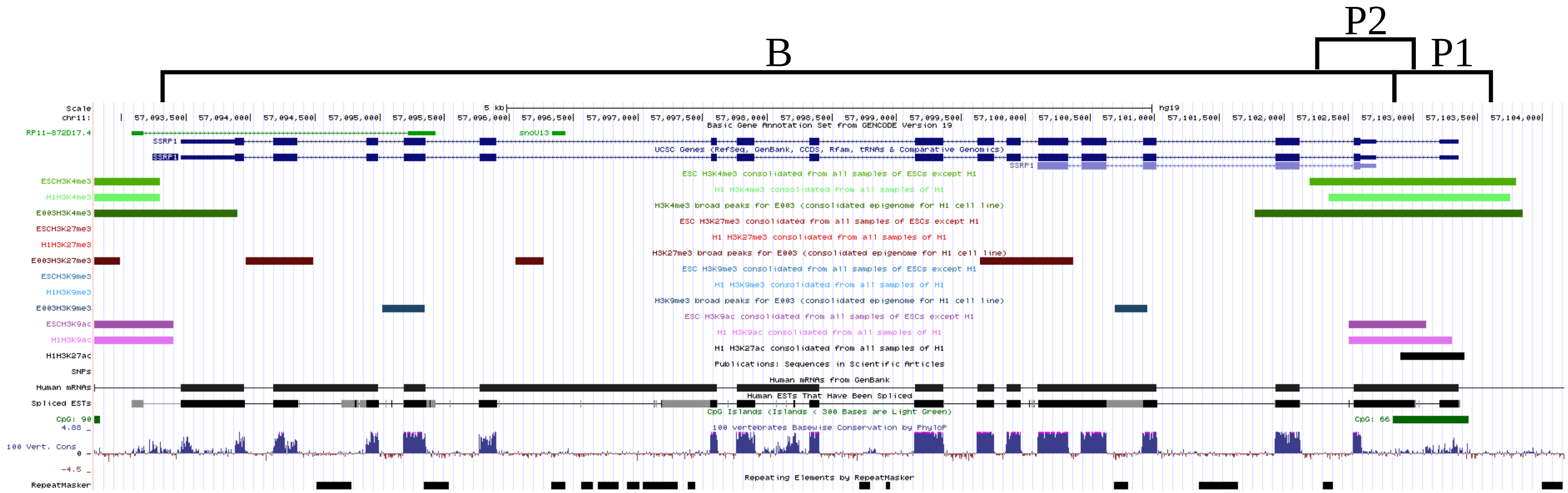

Figure 6.3: Genomic location of SSRP1: The peak-calls are shown below the transcript annotation of SSRP1. Peak-calls for the different modifications and data sets are color coded as described in Table 6.3. The position of the described promoters and gene body are marked with 'P1', 'P2', and 'B', respectively.

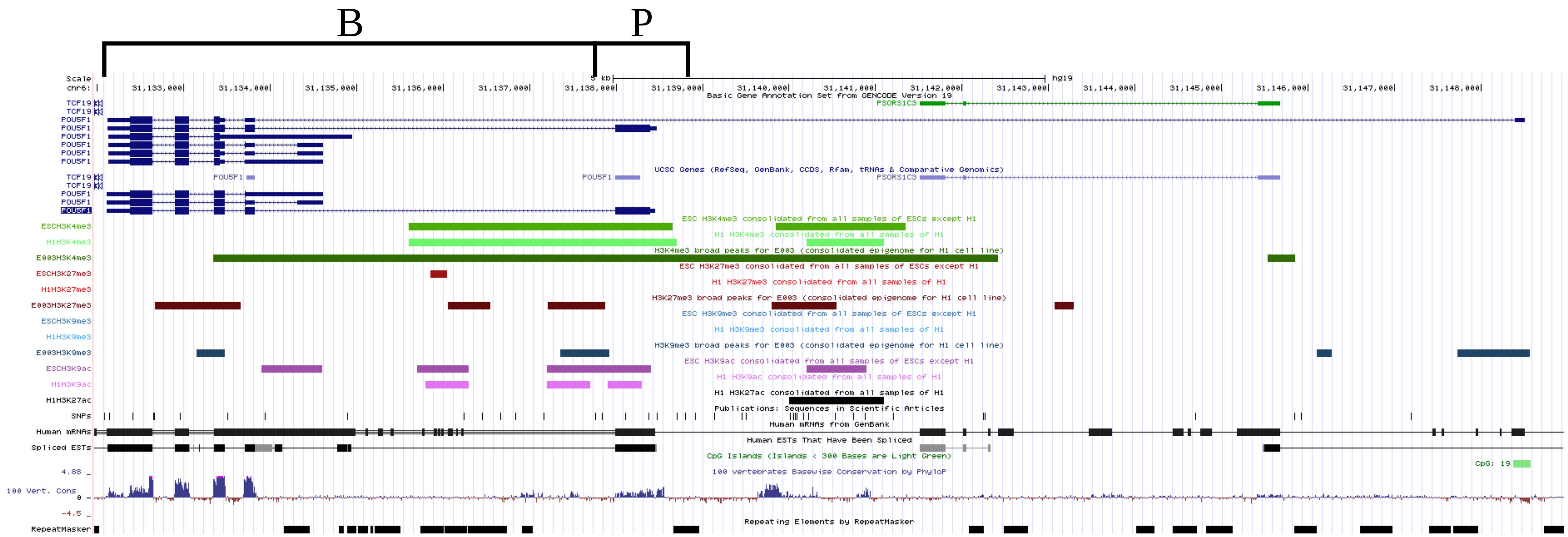

Figure 6.4: Genomic location of OCT4: The peak-calls are shown below the transcript annotation of OCT4. Peak-calls for the different modifications and data sets are color coded as described in Table 6.3. The position of the described promoter and gene body are marked with 'P' and 'B', respectively.

6.4 Overprediction of H3K4me3 and H3K9me3 in the Hox-C and the Hox-D Clusters

The stem cell markers mentioned in the last section are all proteins. In this section, we will have a look at the epigenome state of whole clusters of genes, i.e., the Hox-C and the Hox-D clusters. The Hox clusters (there are also Hox-A and Hox-B) are clusters of transcription factors that are important for embryonal development and differentiation. Therefore, the regulation of these clusters are crucial.

They are conserved in all mammals. However, within the Hox-C cluster, the famous lncRNA HOTAIR is located. HOTAIR was found to drive the regulation of the Hox-D cluster by repressing it. However, it might be that this function is specific to human since it was so far not approved in other mammal and even disproved in mouse.

In Figure 6.5–6.7, we show the HOTAIR locus, the containing Hox-C cluster, and the Hox-D cluster. Given the knowledge about the clusters, one would expect that most of the promoters are inactive (marked with H3K27me3) or poised (marked with H3K4me3). Thus, the genes in the clusters are inactive or are already primed for activity. We would not expect to find H3K9me3 marks in there.

The presence of H3K27me3 at all loci is predicted by all three approaches. However, E003 predicts more H3K4me3 marks. While in the case of HOTAIR this might be correct (even though neither the H1 nor the ESCs based prediction of Sierra Platinum do predict H3K4me3 peaks), the abundance of peaks which do not co-occur with promoter regions is suspicious and may indicate an over-prediction of H3K4me3 marks. Even more suspicious is the massive amount of H3K9me3 marks predicted by the E003 epigenome. This would mean that these regions are strongly repressed which is not found so far for embryonic stem cells. We can furthermore not confirm the presence of H3K9me3 in the clusters using Sierra Platinum on the two embryonic stem cell data sets.

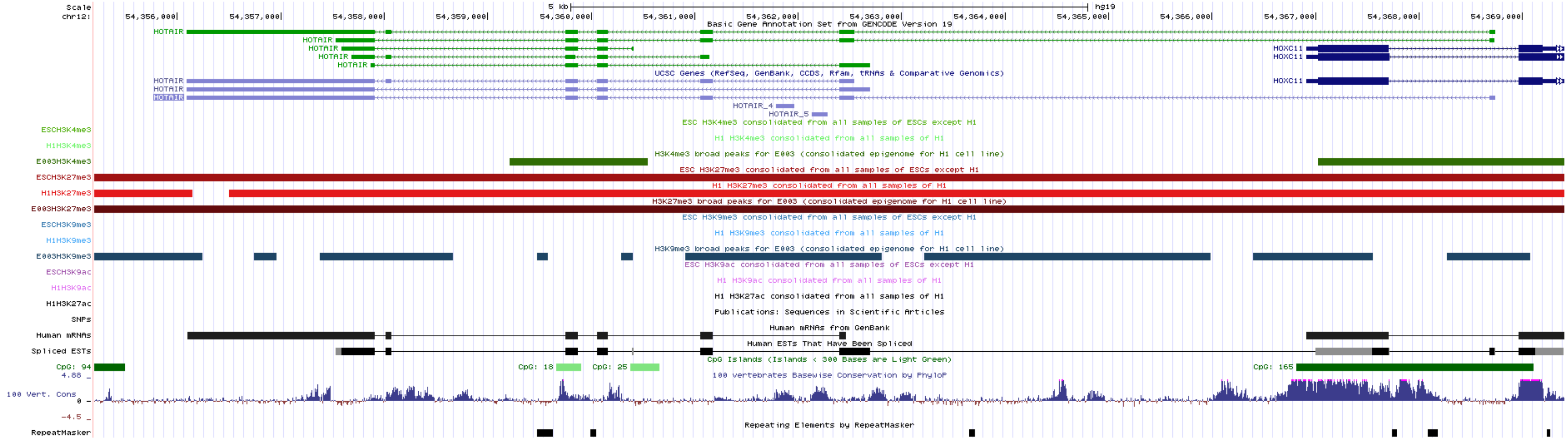

Figure 6.5: Genomic location of HOTAIR locus: The peak-calls are shown below the transcript annotation of HOTAIR. Peak-calls for the different modifications and data sets are color coded as described in Table 6.3.

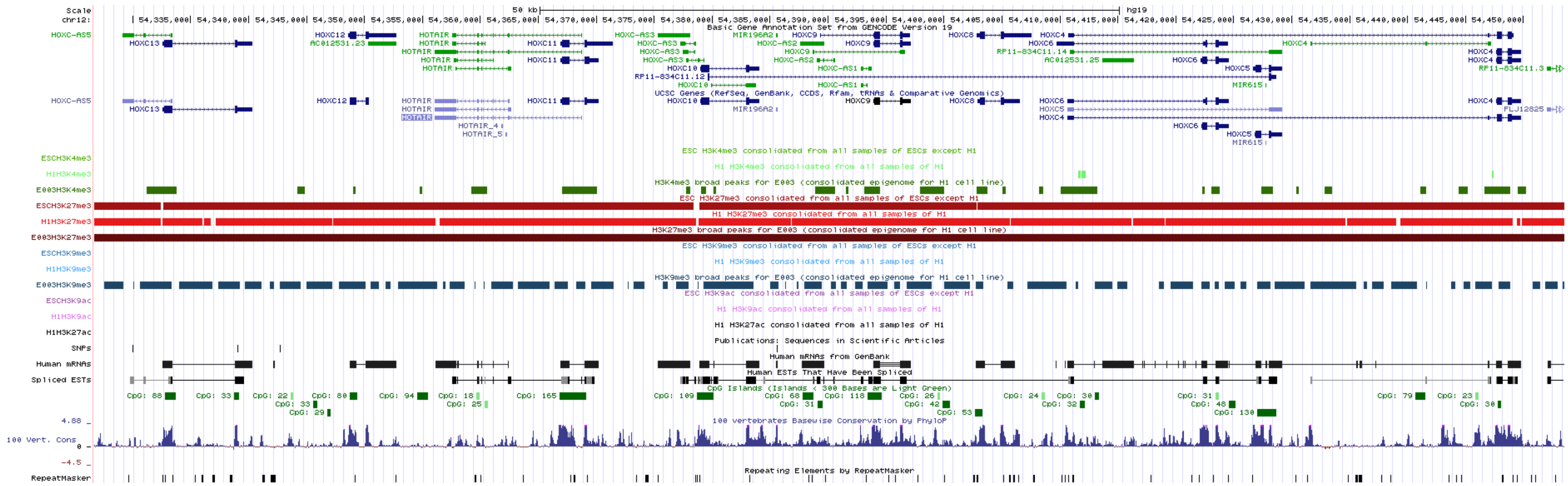

Figure 6.6: Genomic location of Hox-C locus containing the HOTAIR gene: The peak-calls are shown below the transcript annotation of Hox-C. Peak-calls for the different modifications and data sets are color coded as described in Table 6.3.

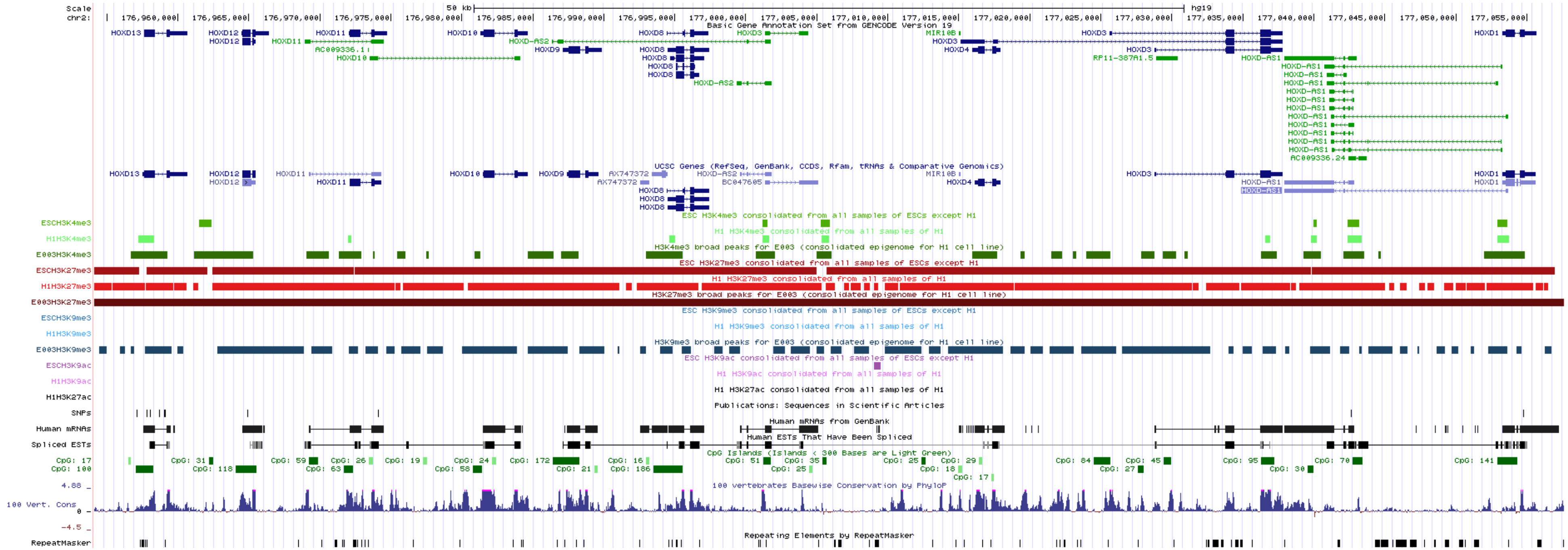

Figure 6.7: Genomic location of Hox-D locus: The peak-calls are shown below the transcript annotation of Hox-D. Peak-calls for the different modifications and data sets are color coded as described in Table 6.3.

6.5 Promoter Analysis

For all three epigenomic states, we have peaks for at least H3K4me3, H3K27me3, and H3K9me3. Those three marks are known to affect the transcriptional outcome of genes when located at their promoter. H3K4me3 is an activating mark and enhances transcription. The other two marks, H3K27me3 and H3K9me3, are associated with repression of genes. Promoters marked with H3K4me3 and H3K27me3 are called poised promoters. While the poised state represses transcription, it can be easily converted into the active state.

Motivated by this functional importance of the chosen marks, we analyzed the promoter states for the three epigenomes. Since all three epigenomic states represent the epigenome of embryonic stem cells and are partially build based on the same data, we expect a high agreement in the epigenomic state of the promoters. We defined the set of promoters based on the gene annotation 'knownGenes' from the UCSC genome browser. For each gene, we defined the promoter region from 2,000nt 5' of the transcription start site to 600nt 3' of the transcription start site. The resulting promoter set is made unique such that each promoter occurs only once in the set.

A promoter is marked with a modification if at least one peak overlaps at least 50% with the promoter. For each promoter and epigenomic state, we thus can define a binary, 3-dimensional vector to indicate with which mark combination the promoter is associated. We will refer to this vector as promoter state derived from an epigenomic state. In total, we obtain 3 promoter states for each promoter.

Agreement on the Promoter State First, we analyze the complete agreement on the promoter state. Thus, we count how often the different epigenomic states predict the same promoter state, i.e., how often the 3-dimensional, binary vectors are equal in all their dimensions. The results are shown in Figure 6.8 (left). All three data sets predict only for 13% of the promoters the same state. Remarkably, the agreement is much stronger between the promoter state of Sierra Platinum of the two different replicates sets (H1 and ESC) than between a Sierra Platinum promoter state and the E003 promoter state. Indeed, almost 80% of the promoter states differ from at least one of the promoter states predicted by H1 and ESC and more than 60% of the promoters have a different state in E003 but the same in H1 and ESC. Only 3% and 6% of the promoter states of ESC and H1, respectively, differ from the promoter state predicted by the corresponding other two prediction.

Now, we will look at the combinations of predicted promoter states. For each data set there are 8 different possible states (3 modifications with 2 possible values, each: $2^3 = 8$). Thus, there are 512 different combinations of these 8 promoter states for the three epigenomic states under consideration ($8^3 = 512$). However, in our data only 317 of the 512 states are present.

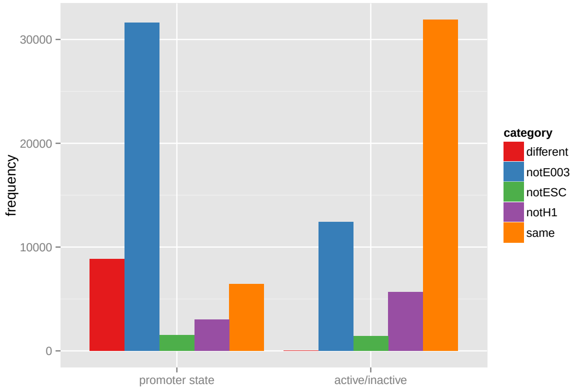

Figure 6.8: Differences and commonalities in the promoter states: (left) Comparison of the promoter state as binary vector. We distinguish 5 classes: red - all data sets predict a different state, blue - H1 and ESC predict the same state but E003 not, green - H1 and E003 predict the same state but not ESC, purple - ESC and E003 predict the same state but not H1, orange - all three predict the same state. (right) Comparison of the promoter using only a classification into 'active' and 'inactive' promoters based on the combination of histone modifications present. Classes are the same as for the left hand side except that the class 'different' (red) cannot exist since we have three data sets but only two possible values.

The 42 most frequent combinations make up 90% of the promoters' states. They are shown in Table 6.4, while Table 6.6 shows the corresponding color coding. While H1 and ESC mostly differ in at most one mark (1 exception only in the top 42), E003 may differ in all three marks and seems to be totally uncorrelated with the predictions of H1 and ESCs. The most frequent combination occurring at more than 11% of the promoters is no modification for H1 and ESCs, and both H3K4me3 and H3K27me3 for E003. Only the second most frequent combination (more than 9% of the promoters) is a combination where all three epigenomic states agree on the promoter state (no modification). For combinations occurring at more than 3% of the promoters H1 and ESC always agree on the promoter state while E003 usually predicts a different state.

Looking only at H1 and ESC (Table 6.5), we find that the majority of the predictions coincide: three combinations with a combined relative frequency of more than 72%. Four combinations differ by at most one modification with a combined relative frequency of more than 17%, and only one combination differs by two modifications with less than 1% relative frequency. Finally, all other combinations have a relative frequency of less than 4‰. Overall, this confirms that H1 and ESC mostly coincide in their predictions.

We furthermore investigated whether at least the predicted transcriptional state of the corresponding gene would be the same or not. Thus, we classified our promoters into active and inactive promoters according to the modification present. Again, we ask how

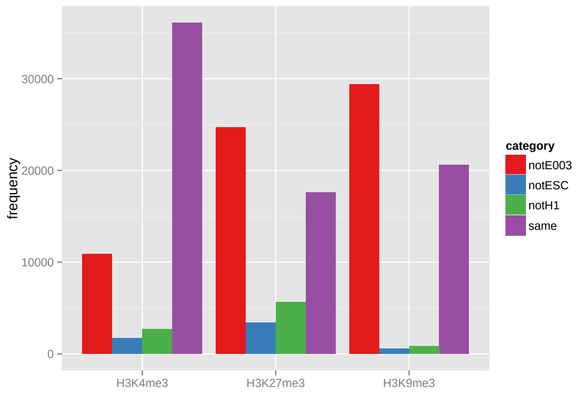

Figure 6.9: Differences and commonalities in the promoter states: (left) Comparison of the H3K4me3 state. (middle) Comparison of the H3K27me3 state. (right) Comparison of the H3K9me3 state. We distinguish 4 classes: blue - H1 and ESC predict the same state but not E003, green - H1 and E003 predict the same state but not ESC, purple - ESC and E003 predict the same state but not H1, orange - all three predict the same state.

strong is the agreement of the three epigenomic states on this promoter classification (see Figure 6.8 (right)). The agreement is much stronger than for the direct promoter state. For more than 60% of the promoters, the same transcriptional outcome is predicted by all three epigenomic states. Still, E003 predicts more often a different transcriptional state (>24%) than the other two (<11% for H1 and <3% for ESC).

The strong agreement in the promoter states, the transcriptional outcome of the epigenomic state predicted by Sierra Platinum, and the evaluation results on the benchmarking data sets indicate that the peaks of E003 may be inaccurate.

Agreement on the Modification For further investigation on the agreement on the promoter states, we now focus on single modifications. Analogously to the promoter state, we ask how often H1, ESC, and E003 agree on the presence/absence state for a specific modification. In other words, how often do all three data sets predict a '1' or a '0' for a promoter. The results for all three modification are shown in Figure 6.9. The agreement on the H3K4me3 state is rather strong: by far the highest bar is purple indicating the same state in all three data sets. Nevertheless, most often E003 predicts a different state than the other two. For the other two modifications, H3K27me3 and H3K9me3, the highest bar is the red bar showing that most often E003 disagrees with the prediction of H1 and ESC.

Table 6.4: Promoter states predicted by H1, ESC, and E003. Equal combinations of promoter states are combined to a single line. The absolute and relative frequencies of each combination are given in the last two columns. Only the combinations in the 90% quantile are shown. Colors represent the promoter state. The corresponding combinations of modifications present are shown in Table 6.6.

H1	ESC	E003	abs-frequency	rel-frequency
red	red	red	5070	9.86688
red	red	blue	4391	8.54546
red	red	green	2907	5.6574
red	red	purple	6034	11.743
red	red	orange	552	1.07426
red	red	yellow	924	1.79823
red	red	brown	905	1.76125
red	red	pink	1815	3.53223
red	green	red	646	1.2572
red	green	blue	830	1.61529
red	green	green	577	1.12292
red	green	purple	1214	2.3626
red	green	yellow	169	0.328896
red	green	pink	365	0.710338
orange	red	red	1136	2.2108
orange	red	green	196	0.655846
orange	red	purple	337	0.381442
orange	red	yellow	170	0.330842
orange	red	pink	248	0.482641
orange	orange	red	588	1.14433
orange	orange	blue	568	1.1054
orange	orange	green	1119	2.17772
orange	orange	purple	1641	3.1936
orange	orange	orange	1183	2.30227
orange	orange	yellow	1316	2.56111
orange	orange	brown	2360	4.59287
orange	orange	pink	3878	7.5471
orange	brown	blue	186	0.36198
orange	brown	green	233	0.453449
orange	brown	purple	325	0.622762
orange	brown	orange	320	0.622762
orange	brown	yellow	434	0.844621
orange	brown	brown	522	1.01588
orange	brown	pink	857	1.66783
brown	red	red	186	0.36198
brown	orange	yellow	198	0.385334
brown	brown	blue	296	0.576055
brown	brown	orange	205	0.398957
brown	brown	yellow	1018	1.98116
brown	brown	pink	327	0.636385

Table 6.5: Promoter states predicted by H1 and ESC. Equal combinations of promoter states are combined to a single line. The absolute and relative frequencies of each combination are given in the last two columns. Only the combinations in the 90% quantile are shown. Colors represent the promoter state. The corresponding combinations of modifications present are shown in Table 6.6.

H1	ESC	abs-frequency	rel-frequency
red	red	22598	43.97871
red	green	3801	7.397244
orange	red	2087	4.061571
orange	orange	12653	24.6244
orange	brown	2877	5.589284
brown	red	186	0.36198
brown	orange	198	0.385334
brown	brown	1846	3.592557

Table 6.6: Color coding used in Tables 6.4 and 6.5. Each color corresponds to one distinct combination of marks presented at the promoter.

Color Encoding	H3K4me3	H3K27me3	H3K9me3
red			
blue	✓		
green		✓	
purple	✓	✓	
orange			✓
yellow	✓		✓
brown		✓	✓
pink	✓	✓	✓

6.6 Peak Coverage Analysis

We analyzed whether the predicted peaks for the combined replicates are supported by the single replicates. Therefore, we calculated the read coverage of each peak by the corresponding replicate. The resulting coverage distributions are simplified by counting only how often peaks are not covered at all, by 1 - 200 reads and by more than 200 reads. For replicates having a high weight, we expect to find a strong support of almost all peaks since those are the replicates having a strong influence on the final p-value. Replicates having a low weight are not expected to fully support the peaks predicted by Sierra Platinum. Those replicates having low weights are noisy or of bad quality and therefore, may not reflect the true epigenome state. Replicates, which are that bad, that we excluded them from peak-calling, are not expected to support the peaks.

Figure 6.10 show the analysis results. In all replicates, most peaks fall into the categories 1-200 reads coverage and more than 200 reads coverage. Thus, in all replicates most peaks have at least a weak support.

Peaks with no support are almost only found when the replicate is strongly down-weighted during peak-calling. For example replicate 16 was down-weighted for peak-calling since the correlation with the other replicates was low and because the tag distribution indicated under-sequencing effects. About 10.000 peaks are unsupported by this replicate. This strongly supports our decision to not rely to much on the replicates and shows that Sierra Platinum overcomes the problem that peaks may not be supported by all replicates. While we still make use of the support of replicate 16 for the remaining 50.000 peaks, the 10.000 unsupported peaks are not strongly affected by the presence of replicate 16.

On the other hand, replicates having a high weight (such as replicate 8 and 9) support most of the peaks. In the case of replicate 8, 9, and 10, most peaks are supported with more than 200 reads. In summary, our peak-calls are supported by the replicates.

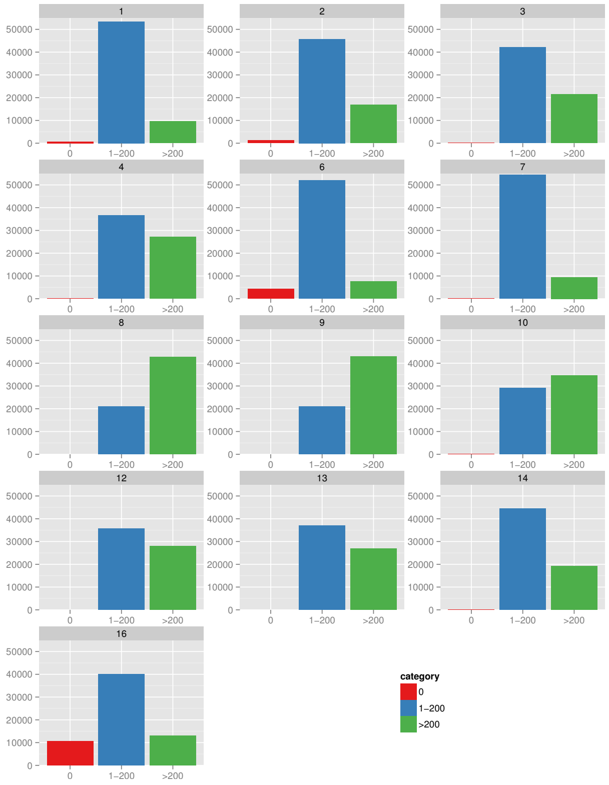

Figure 6.10: Peak coverage for each replicate: For each peak, the number of supporting reads is calculated. Peaks are counted in the categories no supporting reads (red), 1-200 supporting reads (blue), and more than 200 supporting reads (green). The peaks are the H3K4me3 peaks predicted by the data set ESC. Replicate numbers refer to those in Table 6.2a.

Bibliography

[1] A Java API for high-throughput sequencing data (HTS) formats. http://samtools.github.io/htsjdk/.

[2] A set of tools (in Java) for working with next generation sequencing data in the BAM (http://samtools.sourceforge.net) format. http://broadinstitute.github.io/picard/.

[3] Apache Commons IO. https://commons.apache.org/proper/commons-io/.

[4] Apache Commons Logging. https://commons.apache.org/proper/commons-logging/.

[5] Apache Commons Math. https://commons.apache.org/proper/commons-math/.

[6] Apache Commons Net. https://commons.apache.org/proper/commons-net/.

[7] Apache Commons VFS. https://commons.apache.org/proper/commons-vfs/.

[8] Google Gson. https://github.com/google/gson/.

[9] JFreeChart. http://www.jfree.org/jfreechart/.

[10] JSch - Java Secure Channel. http://www.jcraft.com/jsch/.

[11] Simon Andrews. FastQC - A Quality Control tool for High Throughput Sequence Data.

[12] Tanya Barrett, Stephen E. Wilhite, Pierre Ledoux, Carlos Evangelista, Irene F. Kim, Maxim Tomashevsky, Kimberly A. Marshall, Katherine H. Phillippy, Patii M. Sherman, Michelle Holko, Andrey Yefanov, Hyeseung Lee, Naigong Zhang, Cynthia L. Robertson, Nadezhda Serova, Sean Davis, and Alexandra Soboleva. NCBI GEO: archive for functional genomics data sets–update. *Nucleic Acids Res*, 41(Database issue):D991–5, Jan 2013.

[13] Ron Edgar, Michael Domrachev, and Alex E. Lash. Gene Expression Omnibus: NCBI gene expression and hybridization array data repository. *Nucleic Acids Res*, 30(1):207–10, Jan 2002.

[14] Joachim Hartung. A Note on Combining Dependent Tests of Significance. *Biometrical Journal*, 41(7):849–855, 1999.

[15] Laura V. Hedges and Ingram Olkin. *Statistical methods for meta-analysis.* Academic Press, 1985.

[16] Steve Hoffmann, Christian Otto, Stefan Kurtz, Cynthia M. Sharma, Philipp Khaitovich, Jörg Vogel, Peter F. Stadler, and Jörg Hackermüller. Fast mapping of short sequences with mismatches, insertions and deletions using index structures. *PLoS Computational Biology*, 5(9):e1000502, Sep 2009.

[17] Sture Holm. A Simple Sequentially Rejective Multiple Test Procedure. *Scandinavian Journal of Statistics*, 6(2):65–70, 1979.

[18] Peter Humburg. *ChIPsim: Simulation of ChIP-seq experiments*, 2011. R package version 1.18.0.

[19] W. James Kent, Charles W. Sugnet, Terrence S. Furey, Krishna M. Roskin, Tom H. Pringle, Alan M. Zahler, and David Haussler. The human genome browser at UCSC. *Genome research*, 12(6):996–1006, 2002.

[20] Hashem Koohy, Thomas A. Down, Mikhail Spivakov, and Tim Hubbard. A Comparison of Peak Callers Used for DNase-Seq Data. *PLoS ONE*, 9(5):e96303, 05 2014.

[21] Eric S. Lander, Lauren M. Linton, Bruce Birren, and et al. Initial sequencing and analysis of the human genome. *Nature*, 409(6822):860–921, Feb 2001.

[22] Heng Li, Bob Handsaker, Alec Wysoker, Tim Fennell, Jue Ruan, Nils Homer, Gabor Marth, Goncalo Abecasis, Richard Durbin, and 1000 Genome Project Data Processing Subgroup. The Sequence Alignment/Map format and SAMtools. *Bioinformatics*, 25(16):2078–9, Aug 2009.

[23] Eran Meshorer and Tom Misteli. Chromatin in pluripotent embryonic stem cells and differentiation. *Nat Rev Mol Cell Biol*, 7(7):540–6, Jul 2006.

[24] Roadmap Epigenomics Consortium. Integrative analysis of 111 reference human epigenomes. *Nature*, 518(7539):317–30, Feb 2015.

[25] John D. Storey and Robert Tibshirani. Statistical significance for genomewide studies. *Proceedings of the National Academy of Sciences*, 100(16):9440–9445, 2003.

[26] Colin Ware. *Information Visualization: Perception for Design.* Morgan Kaufmann Publishers Inc., San Francisco, CA, USA, 3 edition, 2012.

[27] Elizabeth G. Wilbanks and Marc T. Facciotti. Evaluation of Algorithm Performance in ChIP-Seq Peak Detection. *PLoS ONE*, 5(7):e11471, 07 2010.

[28] Patrick R. Wright, Andreas S. Richter, Kai Papenfort, Martin Mann, Jörg Vogel, Wolfgang R. Hessa, Rolf Backofen, and Jens Georg. Comparative genomics boosts target prediction for bacterial small RNAs. *Proceedings of the National Academy of Science of the United States of America*, 110(37):E3487–96, Sep 2013.

[29] Yanxiao Zhang, Yu-Hsuan Lin, Timothy D. Johnson, Laura S. Rozek, and Maureen A. Sartor. PePr: a peak-calling prioritization pipeline to identify consistent or differential peaks from replicated ChIP-Seq data. *Bioinformatics*, 30(18):2568–75, Sep 2014.

[30] Yong Zhang, Tao Liu, Clifford A. Meyer, Jérôme Eeckhoute, David S. Johnson, Bradley E. Bernstein, Chad Nusbaum, Richard M. Myers, Myles Brown, Wei Li, et al. Model-based analysis of ChIP-Seq (MACS). *Genome biology*, 9(9):R137, 2008.
